# Markovnikov-Type Hydrotrifluoromethylchalcogenation of Unactivated Terminal Alkenes with [Me_4_N][XCF_3_] (X = S, Se) and TfOH

**DOI:** 10.3390/molecules25194535

**Published:** 2020-10-03

**Authors:** Jin Shi, Cheng-Pan Zhang

**Affiliations:** School of Chemistry, Chemical Engineering and Life Science, Wuhan University of Technology, 205 Luoshi Road, Wuhan 430070, China; jshi@whut.edu.cn

**Keywords:** hydrotrifluoromethylselenolation, hydrotrifluoromethylthiolation, Markovnikov-type, terminal alkene, superacid

## Abstract

The first Markovnikov-type hydrotrifluoromethylselenolation of unactivated terminal alkenes with the readily accessible [Me_4_N][SeCF_3_] reagent and the superacid TfOH is reported. The reaction proceeded at room temperature under catalyst- and additive-free conditions to give the branched trifluoromethylselenolated products in good yields. This protocol is also applicable to the Markovnikov-type hydrotrifluoromethylthiolation of unactivated terminal alkenes using [Me_4_N][SCF_3_]/TfOH, but not to the hydrotrifluoromethoxylation with CsOCF_3_/TfOH under the same conditions. The successful hydrotrifluoromethylselenolation and hydrotrifluoromethylthiolation showed simplicity and high regioselectivity, taming the sensitive ^−^XCF_3_ (X = Se, S) anions with TfOH, and offered a convenient method for the straightforward synthesis of branched trifluoromethyl selenoethers and thioethers from unactivated alkenes.

## 1. Introduction

Over the past few decades, fluorinated compounds have attracted great attention in various fields ranging from materials to life sciences because of their unique properties imparted by fluorine [1]. Recently, the special stereoelectronic and physicochemical properties originating from the association of the trifluoromethyl (CF_3_) moiety with chalcogens (e.g., oxygen, sulfur and selenium) have been well documented [2,3,4,5,6,7,8,9,10,11,12,13]. The OCF_3_ and SCF_3_ groups possess strong electronegativity, considerable steric hindrance, and high lipophilicity, which can significantly change the bioactivity of a molecule [2,3,4,5,6,7,8,9,10]. The least-studied SeCF_3_ group represents another important functionality, exhibiting a valuable stereoelectronic nature and lipophilicity, and has immense potential to modulate the potency of agrochemicals and pharmaceuticals [11,12,13]. It is well known that selenium (Se) is an essential trace element in the human body, constituting the crucial parts of antioxidant enzymes that protect cells against the effects of free radicals that are produced during normal oxygen metabolism [14,15,16,17,18]. Although selenium substances have shown very useful biological activities and have been widely utilized in drug design and development [14,15,16,17,18], to our knowledge, the uses of SeCF3-containing compounds have rarely been explored in comparison with those non-fluorinated analogues. This may be attributed to the lack of structural diversity, sufficient synthetic methods, and imperative biological investigation of the trifluoromethylselenolated compounds [2,3,4,5,6,7,8,9,10,11,12,13]. Thus, continuously developing efficient approaches for the construction of different types of SeCF_3_-containing organic scaffolds for future function studies is highly sought-after.

To date, there have been two major strategies for the synthesis of SeCF_3_-substituted molecules: indirect and direct approaches [19,20,21,22,23,24,25,26,27,28,29,30,31,32,33,34,35,36]. The indirect method comprises the trifluoromethylation of selenols, diselenides, and selenocyanates with diverse CF_3_ transfer reagents, including CF_3_SO_2_Na/oxidant, TMSCF_3_/fluoride, HCF_3_/base, CF_3_I/TDAE (tetrakis(dimethylamino)ethylene), and so on. However, these reactions suffer from harsh conditions, low efficiency, poor functional group tolerance, narrow substrate scope, and/or toxicity and pre-functionalization of starting materials, which limit their practical applications [19,20,21,22,23,24,25,26,27]. Notably, the direct trifluoromethylselenolation offers an elegant way to the C-SeCF_3_ bond formation by using elaborate SeCF_3_ reagents, such as CuSeCF_3_, Hg(SeCF_3_)_2_, [(bpy)[Cu(SeCF_3_)]_2_, AgSeCF_3_, [Me_4_N][SeCF_3_], ClSeCF_3_, and TsSeCF_3_ [28,29,30,31,32,33,34,35,36,37,38,39,40,41,42,43,44,45,46,47]. Among these reagents, [Me_4_N][SeCF_3_] is thermally stable, non-volatile, easy to handle, and readily accessible, and has become one of the most important SeCF_3_ sources by far [48]. It has been reported that reactions of [Me_4_N][SeCF_3_] with organic halides, aryl diazonium salts, diaryliodonium triflates, *α*-diazo carbonyls, boronic acids and their esters, terminal alkynes, electron-rich (hetero)arenes, carboxylic acids, 1.3-dicarbonyls and alcohols under transition-metal-catalyzed or -free conditions provide the corresponding trifluoromethyl selenoethers in excellent yields [37,38,39,40,41,42,43,44,45,46,47]. The results have demonstrated a broad prospect of application of [Me_4_N][SeCF_3_] in the direct trifluoromethylselenolation of various structurally diversified molecules.

The carbon–carbon double bond (C = C) is one of the most useful and versatile functional groups in organic chemistry [49,50,51,52,53]. It serves as a central functionality for a wide variety of reactions. Hydrofluoroalkylation of alkenes constitutes a challenging task because of the difficulties of taming the elusive fluoroalkylation reagents with hydrogen sources [54,55,56,57,58,59,60,61,62,63,64,65]. By using CF_3_SH, electrophilic “^+^SCF_3_” reagents, and trifluoromethanesulfonic anhydride (Tf_2_O), the respective hydrotrifluoromethylthiolation of alkenes has been successfully accomplished [66,67,68,69,70]. However, the homologous hydrotrifluoromethylselenolation of unactivated alkenes has not been achieved despite sulfur and selenium being the elements of the same main group [13,71,72,73]. Encouraged by the fact that the readily available [Me_4_N][SeCF_3_] salt is compatible with a superacid in the reactions with *α*-diazo carbonyls [43], we investigated in this article the hydrotrifluoromethylselenolation of alkenes with [Me_4_N][SeCF_3_] in the presence of a strong acid. It was pleasing to find that reactions of unactivated terminal alkenes with [Me_4_N][SeCF_3_] and trifluoromethanesulfonic acid (TfOH) under catalyst- and additive-free conditions formed the secondary alkyl trifluoromethyl selenoethers in good yields.

## 2. Results and Discussions

Initially, 4-(but-3-en-1-yl)-1,1′-biphenyl (**1a**) was chosen as a model substrate to optimize the reaction conditions for hydrotrifluoromethylselenolation with an acid. It was found that reaction of [Me_4_N][SeCF_3_] (1.5 equiv) with a mixture of **1a** and TfOH (1.5 equiv) in dichloromethane at room temperature under a nitrogen atmosphere for 6 h gave (4-([1,1′-biphenyl]-4-yl)butan-2-yl)(trifluoromethyl)selane (**3a**) in 45% yield (Table 1, entry 1). Using the commonly used acids such as CF_3_CO_2_H, concentrated H_2_SO_4_ (98%), anhydrous HCl, 85% H_3_PO_4_ (aq), Et_3_N•3HF, Et_2_O•HBF_4_ (85%), TsOH•H_2_O, and (CF_3_SO_2_)_2_NH instead of TfOH led to no formation of **3a** (Table 1, entries 2–9). These results suggested that TfOH was a better acid than other tested acids for the hydrotrifluoromethylselenolation. The choice of solvent also had an influence on the reaction. Treatment of **1a** and TfOH with [Me_4_N][SeCF_3_] in 1.2-dichloroethane, toluene, chlorobenzene, and 1,1,2-trichloro-1,2,2-trifluoroethane (CFCl_2_CF_2_Cl) under the same conditions provided **3a** with 19–37% yields (Table 1, entries 10–13). If the reaction was run in acetonitrile or (CF_3_)_2_CHOH, no desired product was formed (Table 1, entries 14–15). It seemed that the polar solvents were ineffective for the transformation, as they likely trapped the proton of TfOH and reduced the acidity of TfOH. Moreover, the reaction time could be shortened. Reaction of **1a** and TfOH (1.5 equiv) with [Me_4_N][SeCF_3_] (1.5 equiv) at room temperature for 3 h furnished **3a** in 48% yield, which was comparable to that obtained for 6 h (Table 1, entry 16). In addition, the reactant ratios had an interesting effect on the hydrotrifluoromethylselenolation. Reducing the equivalents of TfOH from 1.5 to 1.0 equiv in the reaction of **1a** and [Me_4_N][SeCF_3_] (1.5 equiv) at room temperature for 3 h produced **3a** in 71% yield (68% isolated yield) (Table 1, entry 17). However, varying the molar ratio of **1a**/[Me_4_N][SeCF_3_]/TfOH from 1:1.5:1 to 1:1.5:2, 1:2:1, 1:1:1, and 1.5:1:1 resulted in 15%, 57%, 39%, and 42% of **3a**, respectively (Table 1, entries 18–21). Remarkably, if **1a** (2 equiv) reacted with TfOH (1.0 equiv) and [Me_4_N][SeCF_3_] (2 equiv) at room temperature under N_2_ for 3 h, **3a** was formed in 83% yield (79% isolated yield) (Table 1, entry 22). Furthermore, the reaction at room temperature was preferable as either elevating or lowering the temperature decreased the yield of **3a** (Table S3 (see Supporting Information)). Addition of gold, copper, and silver salts as Lewis acids to the reaction mixtures of **1a**, [Me_4_N][SeCF_3_] (1.5 equiv), and TfOH (1.0 equiv) did not promote the production of **3a** (see Table S6). It was noteworthy that from the above successful hydrotrifluoromethylselenolations, branched trifluoromethyl selenoether was isolated rather than the linear isomer, showing excellent regioselectivity of the reaction.

**Scheme 1:**
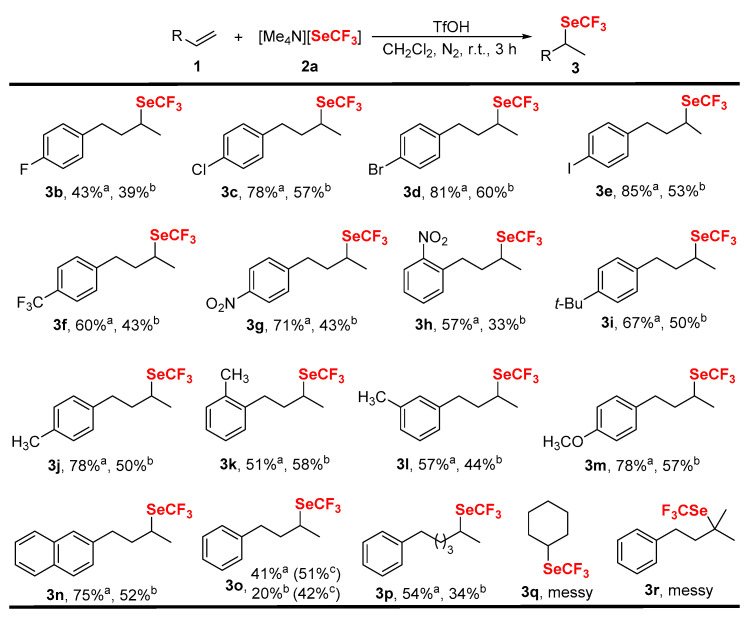
Hydrotrifluoromethylselenolation of unactivated terminal alkenes with [Me_4_N][SeCF_3_] and TfOH. ^a^ Reaction conditions: To a solution of **1** (0.4 mmol) in CH_2_Cl_2_ (1 mL) was added a solution of TfOH (0.2 mmol) in CH_2_Cl_2_ (1 mL), followed by addition of [Me_4_N][SeCF_3_] (0.4 mmol) within 1 min. The mixture was reacted at room temperature under N_2_ for 3 h. Isolated yields. ^b^ The same reaction was run with **1** (0.2 mmol), TfOH (0.2 mmol) and [Me_4_N][SeCF_3_] (0.3 mmol). Isolated yields. ^c 19^F NMR yields.

Next, the combinations of **1** (2 equiv)/TfOH (1.0 equiv)/[Me_4_N][SeCF_3_] (2 equiv)/CH_2_Cl_2_/r.t./3 h and **1** (1.0 equiv)/TfOH (1.0 equiv)/[Me_4_N][SeCF_3_] (1.5 equiv)/CH_2_Cl_2_/r.t./3 h (Table 1, entries 17 and 22) were employed to probe the substrate scope of the reaction (Scheme 1). To our delight, a range of but-3-en-1-ylarenes with functional groups such as F, Cl, Br, I, CF_3_, NO_2_, *t*-Bu, CH_3_, and OCH_3_ on the aryl rings were favorably converted to form the corresponding Markovnikov-type hydrotrifluoromethylselenolated products (**3a**–**m**) under the two standard conditions in good yields. The position of the substituents on the aryl groups of but-3-en-1-ylarenes probably had an influence on the hydrotrifluoromethylselenolation as the reactions of alkenes with either electron-donating or withdrawing *para*-substituents on the aryl functionalities (e.g., **3g** and **3j**) gave higher yields than those of the substrates bearing *ortho*- and/or *meta*-substitution of the same groups on the aryl moieties (e.g., **3h**, **3k**, and **3l**). The reasons for these differences remained unclear. Treatment of 2-(but-3-en-1-yl)naphthalene (**1n**) with [Me_4_N][SeCF_3_] and TfOH under the two standard conditions formed **3n** in 75% and 52% yields, respectively. The non-substituted but-3-en-1-ylbenzene (**1o**) and hex-5-en-1-ylbenzene (**1p**) reacted similarly with [Me_4_N][SeCF_3_] and TfOH to afford the branched trifluoromethyl selenoethers in 20–54% yields. It should be mentioned that the assembly of **1** (2 equiv)/TfOH (1.0 equiv)/[Me_4_N][SeCF_3_] (2 equiv)/CH_2_Cl_2_/r.t./3 h provided higher yields of the desired products than that of **1** (1.0 equiv)/TfOH (1.0 equiv)/[Me_4_N][SeCF_3_] (1.5 equiv)/CH_2_Cl_2_/r.t./3 h in most cases, suggesting that the use of excess alkenes was beneficial for promotion of the reaction. Unfortunately, the activated alkenes (e.g., styrene and 4-vinyl-1,1′-biphenyl), internal alkenes (e.g., cyclohexene (**1q**) and (*E*)-tetradec-7-ene), and geminal disubstituted alkenes (e.g., (3-methylbut-3-en-1-yl)benzene (**1r**) and (3-bromobut-3-en-1-yl)benzene) reacted with [Me_4_N][SeCF_3_] and TfOH under the standard conditions to give complicated mixtures, in which the pure products were not isolated even though the ^19^F NMR analysis of the reaction mixtures showed the formation of the corresponding desired products.

**Scheme 2:**
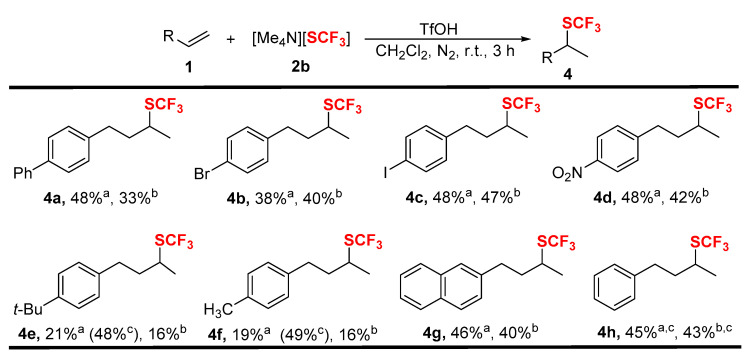
Hydrotrifluoromethylthiolation of unactivated terminal alkenes with [Me_4_N][SCF_3_] and TfOH. ^a^ Reaction conditions: To a solution of **1** (0.4 mmol) in CH_2_Cl_2_ (1 mL) was added a solution of TfOH (0.2 mmol) in CH_2_Cl_2_ (1 mL), followed by addition of [Me_4_N][SCF_3_] (0.4 mmol) within 1 min. The mixture was reacted at room temperature under N_2_ for 3 h. Isolated yields. ^b^ The same reaction was run with **1** (0.2 mmol), TfOH (0.2 mmol) and [Me_4_N][SCF_3_] (0.3 mmol). Isolated yields. ^c 19^F NMR yields.

Since the ^−^SCF_3_ and ^−^OCF_3_ anions have similar reactivities to that of the ^−^SeCF_3_ anion, the analogous hydrotrifluoromethylthiolation and hydrotrifluoromethoxylation of unactivated terminal alkenes with the nucleophilic SCF_3_ and OCF_3_ reagents in the presence of a superacid were also examined. Encouragingly, the straightforward reactions of **1a** and TfOH with [Me_4_N][SCF_3_] under the two standard conditions that were used for the hydrotrifluoromethylselenolation of alkene with [Me_4_N][SeCF_3_] gave the expected branched hydrotrifluoromethylthiolated product (**4a**) in 48% and 33% yield, respectively (Scheme 2). By this method, a series of unactivated terminal alkenes, such as **1d**, **1e**, **1g**, **1i**, **1j**, **1n**, and **1o**, were readily transformed, affording the corresponding products (**4b**–**h**) in 16–49% yields. These yields were generally lower than those of the homologous trifluoromethylselenolated products, which might be caused by the poorer nucleophilicity of the ^−^SCF_3_ anion than the ^−^SeCF_3_ anion in [Me_4_N][XCF_3_] salts. Moreover, because products **4e** and **4f** had very similar polarity to the alkenes and could not be fully separated from the starting alkenes by column chromatography and preparative TLC plate, they were finally obtained with much lower isolated yields (19–21%) than the ^19^F NMR yields (48–49%). Nonetheless, reactions of alkene (**1a**) with CsOCF_3_ and TfOH under the standard or modified conditions did not form the hydrotrifluoromethoxylated product (see Supplementary Materials), which was determined by ^19^F NMR analysis of the reaction mixtures. These observations implied that the ^−^SeCF_3_ anion is a more suitable reagent than the ^−^SCF_3_ and ^−^OCF_3_ anions for the hydrotrifluoromethylchalcogenation of unactivated terminal alkene in the presence of a superacid.

It should be noted that the charging sequence of alkene, [Me_4_N][SeCF_3_], and TfOH significantly affected the hydrotrifluoromethylselenolation (Table 2). Addition of [Me_4_N][SeCF_3_] (2 equiv) to a solution of **1a** (2 equiv) and TfOH in CH_2_Cl_2_ that was pre-mixed at room temperature for 1 min or 1 h provided **3a** in 83% or 65% yield, respectively, under the standard conditions. If a solution of TfOH in CH_2_Cl_2_ was introduced into a mixture of [Me_4_N][SeCF_3_] (2 equiv) and **1a** (2 equiv) that was pre-mixed in CH_2_Cl_2_ at room temperature under N_2_ for 1 min or 1 h, **3a** was obtained in 47% or 40% yield, respectively. Surprisingly, if a solution of **1a** (2 equiv) in CH_2_Cl_2_ was treated with a mixture of [Me_4_N][SeCF_3_] (2 equiv) and TfOH that was pre-mixed in CH_2_Cl_2_ at room temperature under N_2_ for 1 min or 1 h, no desired product was formed. When a combination of **1a** (1.0 equiv)/TfOH (1.0 equiv)/[Me_4_N][SeCF_3_] (1.5 equiv) was used instead of **1a** (2.0 equiv)/TfOH (1.0 equiv)/[Me_4_N][SeCF_3_] (2.0 equiv) for the reactions, similar results were also observed (Table 2). These results demonstrated that pre-activation of **1a** by TfOH was very essential for the hydrotrifluoromethylselenolation of **1a** with [Me_4_N][SeCF_3_], whereas pre-treatment of [Me_4_N][SeCF_3_] with TfOH led to complete failure of the reaction. Since pre-mixing **1a** with TfOH for 1 h gave a lower yield of **3a** (65% or 11%) than that (83% or 71%) for 1 min, the longer pre-treatment of **1a** and TfOH might harm the subsequent hydrotrifluoromethylselenolation, which was likely attributed to the decay of the key reactive intermediates. Furthermore, the ^1^H and ^19^F NMR analysis of a reaction mixture of **1a** and TfOH in the absence of [Me_4_N][SeCF_3_] showed the fast conversion of **1a** and formation of an alkyl triflate (Figures S13–S24 (see Supplementary Materials)), but the similar measurement of a mixture of [Me_4_N][SeCF_3_] and TfOH in the absence of **1a** revealed a very complicated system (Figures S1–S12 (see Supplementary Materials)). These outcomes might explain in part the distinct results of the reactions with different charging sequences under the standard conditions.

**Scheme 3:**
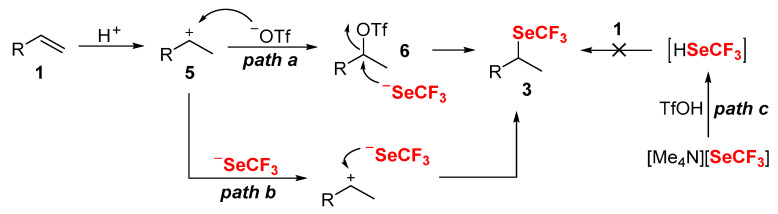
A proposed reaction mechanism for TfOH-mediated hydrotrifluoromethylselenolation of unactivated terminal alkene.

On the basis of the above observations, a plausible reaction mechanism for the TfOH-mediated hydrotrifluoromethylselenolation of unactivated terminal alkene was suggested in Scheme 3. First, Markovnikov-type protonation of the carbon–carbon double bond of alkene at the terminal carbon site forms a relatively stable alkyl cation intermediate (**5**), which is combined with the ^−^OTf anion to yield a secondary alkyl triflate (**6**). Then, nucleophilic attack of **6** by the free ^−^SeCF_3_ anion generates the final product (**3**) (***path* a**). Alternatively, direct trifluoromethylselenolation of the secondary carbon cation (**5**) upon its formation by ^−^SeCF_3_ can also form the desired product (*path* **b**). *Path* **a** could be the most plausible process in the reactions according to the NMR experiments (Figures S13–S24) if alkene was pre-mixed with TfOH before addition of [Me_4_N][SeCF_3_]. Nevertheless, the in situ generation of HSeCF_3_ from metathesis of [Me_4_N][SeCF_3_] and TfOH followed by addition of HSeCF_3_ to the carbon–carbon double bond of alkene could be excluded (*path* **c**) as the reactions of **1a** with mixtures of [Me_4_N][SeCF_3_] and TfOH that were pre-mixed at room temperature for some time gave no hydrotrifluoromethylselenolated product (Table 2).

## 3. Materials and Methods

### 3.1. General Information

All reactions were carried out under a nitrogen atmosphere unless otherwise specified. The NMR spectra were recorded in CDCl_3_ on a 500 (for ^1^H), 471 (for ^19^F), and 126 MHz (for ^13^C) spectrometer. All chemical shifts were reported in ppm relative to TMS (0 ppm) for ^1^H NMR and PhOCF_3_ (58.0 ppm) or PhCF_3_ (63.0 ppm) for ^19^F NMR as an internal or external standard. The coupling constants were reported in Hertz (Hz). The following abbreviations were used to explain the multiplicities: s = singlet, d = doublet, t = triplet, q = quartet, m = multiplet. The HPLC experiments were carried out on a Wufeng LC-100 II instrument (column: Shodex, C18, 5 μm, 4.6 × 250 mm), and the HPLC yields of the product were determined by using the corresponding pure compound as the external standard. MS experiments were performed on a TOF-Q ESI or EI instrument. Reagents [Me_4_N][SeCF_3_] (**2a**), [Me_4_N][SCF_3_] (**2b**), and CsOCF_3_ were synthesized according to the literature [74,75]. Substrates **1a**–**f** [76], **1g**–**1h** [77], **1i**–**1l** [76], **1m**–**1n** [78], and **1p**–**1r [78]** were synthesized according to the literature. Solvents were dried before use according to the literature [79]. Other reagents in the reactions were all purchased from the commercial sources and used without further purification.

### 3.2. General Procedure for Hydrotrifluoromethylselenolation of Unactivated Terminal Alkenes

Under a nitrogen atmosphere, a Schlenk tube was charged with **1** (0.4 or 0.2 mmol) and CH_2_Cl_2_ (1 mL) with stirring. A solution of TfOH (0.2 mmol) in CH_2_Cl_2_ was added, followed by addition of [Me_4_N][SeCF_3_] (**2a**, 0.4 or 0.3 mmol) within 1 min. The mixture was reacted at room temperature under N_2_ for 3 h and concentrated to dryness under reduced pressure. The residue was purified by flash column chromatography on silica gel using petroleum ether or a mixture of petroleum ether and ethyl acetate as eluents to give the trifluoromethylselenolated products (**3**).

(4-([1,1′-Biphenyl]-4-yl)butan-2-yl)(trifluoromethyl)selane (**3a**)

Yellow oil, 59.4 mg (79%, **1a**:[Me_4_N][SeCF_3_]:TfOH = 2:2:1) and 50.8 mg (68%, **1a**:[Me_4_N][SeCF_3_]:TfOH = 1:1.5:1), petroleum ether as eluent for column chromatography. ^1^H NMR (500 MHz, CDCl_3_) δ 7.62 (d, *J* = 8.1 Hz, 2H), 7.57 (d, *J* = 7.8 Hz, 2H), 7.47 (t, *J* = 7.7 Hz, 2H), 7.37 (t, *J* = 7.9 Hz, 1H), 7.30 (d, *J* = 8.0 Hz, 2H), 3.59 (m, 1H), 2.89–2.79 (m, 2H), 2.19–2.03 (m, 2H), 1.68 (d, *J* = 6.9 Hz, 3H). ^19^F NMR (471 MHz, CDCl_3_) δ −31.9 (s, 3F). ^13^C NMR (126 MHz, CDCl_3_) δ 141.0, 140.0, 139.2, 128.9, 128.8, 127.3, 127.2, 127.0, 123.2 (q, *J* = 331.1 Hz), 39.5, 39.2, 33.4, 23.1. IR (KBr): 3085, 3057, 3029, 2958, 2926, 2855, 1601, 1564, 1520, 1487, 1450, 1409, 1383, 1260, 1212, 1098, 1008, 838, 761, 738, 697, 598, 550, 507 cm^−1^. HRMS-ESI (*m*/*z*) calcd. for C_17_H_18_F_3_Se ([M + H]^+^): 359.0520; found: 359.0511.

(4-(4-Fluorophenyl)butan-2-yl)(trifluoromethyl)selane (**3b**)

Yellow oil, 25.8 mg (43%, **1b**:[Me_4_N][SeCF_3_]:TfOH = 2:2:1) and 23.4 mg (39%, **1b**:[Me_4_N][SeCF_3_]:TfOH = 1:1.5:1), petroleum ether as eluent for column chromatography. ^1^H NMR (500 MHz, CDCl_3_) δ 7.42 (d, *J* = 8.3 Hz, 2H), 7.07 (d, *J* = 8.3 Hz, 2H), 3.50 (m, 1H), 2.78–2.68 (m, 2H), 2.09–1.93 (m, 2H), 1.62 (d, *J* = 7.0 Hz, 3H). ^19^F NMR (471 MHz, CDCl_3_) δ −32.0 (s, 3F), −117.2 (m, 1F). ^13^C NMR (126 MHz, CDCl_3_) δ 161.5 (d, *J* = 244.5 Hz), 136.4 (d, *J* = 3.3 Hz), 129.7 (d, *J* = 7.8 Hz), 123.1 (q, *J* = 330.8 Hz), 115.3 (d, *J* = 21.1 Hz), 39.3, 39.2, 32.9, 23.0. IR (KBr): 3041, 2927, 2856, 1602, 1511, 1455, 1383, 1259, 1224, 1157, 1098, 1016, 827, 760, 738, 543 cm^−1^. HRMS-EI (*m*/*z*) calcd. for C_11_H_12_F_4_^74^Se: 294.0100; found: 294.0109.

(4-(4-Chlorophenyl)butan-2-yl)(trifluoromethyl)selane (**3c**)

Yellow oil, 49.3 mg (78%, **1c**:[Me_4_N][SeCF_3_]:TfOH = 2:2:1) and 36.0 mg (57%, **1c**:[Me_4_N][SeCF_3_]:TfOH = 1:1.5:1), petroleum ether as eluent for column chromatography. ^1^H NMR (500 MHz, CDCl_3_) δ 7.26 (d, *J* = 8.4 Hz, 2H), 7.12 (d, *J* = 8.3 Hz, 2H), 3.50 (m, 1H), 2.79–2.69 (m, 2H), 2.09–1.94 (m, 2H), 1.62 (d, *J* = 6.9 Hz, 3H). ^19^F NMR (471 MHz, CDCl_3_) δ −32.0 (s, 3F). ^13^C NMR (126 MHz, CDCl_3_) δ 139.3, 132.0, 129.7, 128.7, 123.1 (q, *J* = 331.3 Hz), 39.2, 39.1, 33.0, 23.0. IR (KBr): 3084, 3028, 2927, 2856, 1895, 1731, 1598, 1493, 1455, 1408, 1383, 1290, 1279, 1260, 1232, 1214, 1096, 1035, 1016, 832, 818, 807, 778, 738, 715, 672, 663, 631, 523 cm^−1^. HRMS-ESI (*m*/*z*) calcd. for C_10_H_12_Cl ([M – SeCF_3_]^+^): 167.0622; found: 167.0624.

(4-(4-Bromophenyl)butan-2-yl)(trifluoromethyl)selane (**3d**)

Yellow oil, 58.3 mg (81%, **1d**:[Me_4_N][SeCF_3_]:TfOH = 2:2:1) and 43.2 mg (60%, **1d**:[Me_4_N][SeCF_3_]:TfOH = 1:1.5:1), petroleum ether as eluent for column chromatography. ^1^H NMR (500 MHz, CDCl_3_) δ 7.42 (d, *J* = 8.3 Hz, 2H), 7.07 (d, *J* = 8.3 Hz, 2H), 3.50 (m, 1H), 2.78-2.68 (m, 2H), 2.09-1.93 (m, 2H), 1.62 (d, *J* = 6.9 Hz, 3H). ^19^F NMR (471 MHz, CDCl_3_) δ −32.0 (s, 3F). ^13^C NMR (126 MHz, CDCl_3_) δ 139.8, 131.6, 130.1, 123.1 (q, *J* = 331.0 Hz), 120.0, 39.2, 39.0, 33.1, 23.0. IR (KBr): 3025, 2961, 2926, 2863, 1489, 1454, 1405, 1383, 1278, 1260, 1214, 1097, 1074, 1012, 960, 897, 828, 813, 802, 770, 738, 711, 654, 634, 605, 515 cm^-1^. HRMS-EI (*m*/*z*) calcd. for C_11_H_12_F_3_Br^74^Se: 353.9299; found: 353.9307.

(4-(4-Iodophenyl)butan-2-yl)(trifluoromethyl)selane (**3e**)

Yellow oil, 69.3 mg (85%, **1e**:[Me_4_N][SeCF_3_]:TfOH = 2:2:1) and 43.2 mg (53%, **1e**:[Me_4_N][SeCF_3_]:TfOH = 1:1.5:1), petroleum ether as eluent for column chromatography. ^1^H NMR (500 MHz, CDCl_3_) δ 7.62 (d, *J* = 8.3 Hz, 2H), 6.95 (d, *J* = 8.3 Hz, 2H), 3.49 (m, 1H), 2.76–2.67 (m, 2H), 2.08–1.93 (m, 2H), 1.62 (d, *J* = 6.9 Hz, 3H). ^19^F NMR (471 MHz, CDCl_3_) δ −32.0 (s, 3F). ^13^C NMR (126 MHz, CDCl_3_) δ 140.5, 137.6, 130.5, 123.1 (q, *J* = 331.2 Hz), 91.3, 39.2, 39.0, 33.2, 23.0. IR (KBr): 3019, 2960, 2925, 2854, 1485, 1454, 1401, 1382, 1291, 1275, 1260, 1230, 1213, 1202, 1097, 1063, 1035, 1007, 897, 826, 799, 766, 738, 709, 512 cm^−1^. HRMS-ESI (*m*/*z*) calcd. for C_9_H_12_ISe ([M – CF_3_]^−^): 337.9196; found: 337.9198.

(Trifluoromethyl)(4-(4-(trifluoromethyl)phenyl)butan-2-yl)selane (**3f**)

Yellow oil, 42.0 mg (60%, **1f**:[Me_4_N][SeCF_3_]:TfOH = 2:2:1) and 30.1 mg (43%, **1f**:[Me_4_N][SeCF_3_]:TfOH = 1:1.5:1), petroleum ether as eluent for column chromatography. ^1^H NMR (500 MHz, CDCl_3_) δ 7.56 (d, *J* = 8.1 Hz, 2H), 7.31 (d, *J* = 8.0 Hz, 2H), 3.51 (m, 1H), 2.89–2.79 (m, 2H), 2.13–1.98 (m, 2H), 1.64 (d, *J* = 6.9 Hz, 3H). ^19^F NMR (471 MHz, CDCl_3_) δ −32.0 (s, 3F), -62.4 (s, 3F). ^13^C NMR (126 MHz, CDCl_3_) δ 145.0, 128.7, 128.6 (q, *J* = 32.4 Hz), 125.5 (q, *J* = 3.8 Hz) 124.3 (q, *J* = 271.5 Hz), 123.1 (q, *J* = 330.8 Hz), 39.2, 38.9, 33.5, 23.0. IR (KBr): 2958, 2929, 2858, 1620, 1456, 1419, 1384, 1327, 1165, 1117, 1095, 1068, 1019, 899, 840, 823, 738, 650, 633, 599 cm^−1^. HRMS-EI (*m*/*z*) calcd. for C_12_H_12_F_6_^74^Se: 344.0068; found: 344.0061.

(4-(4-Nitrophenyl)butan-2-yl)(trifluoromethyl)selane (**3g**)

Yellow oil, 46.4 mg (71%, **1g**:[Me_4_N][SeCF_3_]:TfOH = 2:2:1) and 28.1 mg (43%, **1g**:[Me_4_N][SeCF_3_]:TfOH = 1:1.5:1), a mixture of petroleum ether and ethyl acetate (40:1 (*v/v*)) as eluents for column chromatography. ^1^H NMR (500 MHz, CDCl_3_) δ 8.17 (d, *J* = 8.4 Hz, 2H), 7.36 (d, *J* = 8.4 Hz, 2H), 3.50 (m, 1H), 2.94–2.84 (m, 2H), 2.14–2.00 (m, 2H), 1.64 (d, *J* = 6.9 Hz, 3H). ^19^F NMR (471 MHz, CDCl_3_) δ −32.0 (s, 3F). ^13^C NMR (126 MHz, CDCl_3_) δ 148.7, 146.7, 129.2, 123.9, 123.0 (q, *J* = 331.1 Hz), 39.0, 38.7, 33.6, 23.0. IR (KBr): 3112, 3080, 2950, 2929, 2856, 2453, 2217, 1927, 1727, 1602, 1520, 1495, 1455, 1384, 1347, 1319, 1289, 1236, 1214, 1180, 1098, 1035, 1016, 973, 891, 858, 848, 806, 768, 747, 738, 698, 660, 647, 632, 619, 513 cm^−1^. HRMS-ESI (*m*/*z*) calcd. for C_11_H_13_F_3_NO_2_Se ([M + H]^+^): 328.0058; found: 328.0060.

(4-(2-Nitrophenyl)butan-2-yl)(trifluoromethyl)selane (**3h**)

Yellow oil, 37.3 mg (57%, **1h**:[Me_4_N][SeCF_3_]:TfOH = 2:2:1) and 21.6 mg (33%, **1h**:[Me_4_N][SeCF_3_]:TfOH = 1:1.5:1), a mixture of petroleum ether and ethyl acetate (40:1 (*v/v*)) as eluents for column chromatography. ^1^H NMR (500 MHz, CDCl_3_) δ 7.94 (d, *J* = 8.1 Hz, 1H), 7.55 (t, *J* = 7.6 Hz, 1H), 7.40–7.36 (m, 2H), 3.60 (m, 1H), 3.11–2.98 (m, 2H), 2.14–2.03 (m, 2H), 1.66 (d, *J* = 7.0 Hz, 3H). ^19^F NMR (471 MHz, CDCl_3_) δ −32.1 (s, 3F). ^13^C NMR (126 MHz, CDCl_3_) δ 149.2, 136.1, 133.2, 132.0, 127.5, 125.0, 123.1 (q, *J* = 330.5 Hz), 39.5, 38.5, 31.3, 22.8. IR (KBr): 3069, 2964, 2928, 2856, 1611, 1579, 1527, 1481, 1457, 1383, 1348, 1281, 1231, 1215, 1099, 959, 861, 813, 787, 739, 702, 667 cm^−1^. HRMS-EI (*m*/*z*) calcd. for C_11_H_12_F_3_NO_2_^74^Se: 321.0045; found: 321.0039.

(4-(4-(*Tert*-butyl)phenyl)butan-2-yl)(trifluoromethyl)selane (**3i**)

Yellow oil, 45.3 mg (67%, **1i**:[Me_4_N][SeCF_3_]:TfOH = 2:2:1) and 33.8 mg (50%, **1i**:[Me_4_N][SeCF_3_]:TfOH = 1:1.5:1), petroleum ether as eluent for column chromatography. ^1^H NMR (500 MHz, CDCl_3_) δ 7.33 (d, *J* = 8.1 Hz, 2H), 7.13 (d, *J* = 8.1 Hz, 2H), 3.55 (m, 1H), 2.79–2.69 (m, 2H), 2.12–1.96 (m, 2H), 1.64 (d, *J* = 6.9 Hz, 3H), 1.32 (s, 9H). ^19^F NMR (471 MHz, CDCl_3_) δ −32.0 (s, 3F). ^13^C NMR (126 MHz, CDCl_3_) δ 149.0, 137.8, 128.0, 125.4, 123.2 (q, *J* = 331.2 Hz), 39.5, 39.2, 34.4, 33.1, 31.4, 23.0. IR (KBr): 3056, 3024, 2964, 2868, 1517, 1457, 1412, 1394, 1382, 1364, 1269, 1234, 1215, 1203, 1098, 1035, 1019, 829, 815, 738, 569 cm^−1^. HRMS-EI (*m*/*z*) calcd. for C_15_H_21_F_3_^74^Se: 332.0820; found: 332.0816.

(4-(*p*-Tolyl)butan-2-yl)(trifluoromethyl)selane (**3j**)

Yellow oil, 46.2 mg (78%, **1j**:[Me_4_N][SeCF_3_]:TfOH = 2:2:1) and 29.6 mg (50%, **1j**:[Me_4_N][SeCF_3_]:TfOH = 1:1.5:1), petroleum ether as eluent for column chromatography. ^1^H NMR (500 MHz, CDCl_3_) δ 7.12–7.08 (m, 4H), 3.53 (m, 1H), 2.78–2.68 (m, 2H), 2.33 (s, 3H), 2.10–1.94 (m, 2H), 1.63 (d, *J* = 6.9 Hz, 3H). ^19^F NMR (471 MHz, CDCl_3_) δ −32.0 (s, 3F). ^13^C NMR (126 MHz, CDCl_3_) δ 137.7, 135.7, 129.2, 128.3, 123.2 (q, *J* = 330.5 Hz), 39.4, 39.3, 33.2, 23.0, 21.0. IR (KBr): 3048, 3020, 2925, 2859, 1516, 1454, 1381, 1260, 1214, 1097, 1022, 830, 807, 738, 543 cm^−1^. HRMS-EI (*m*/*z*) calcd. for C_12_H_15_F_3_^74^Se: 290.0351; found: 290.0349.

(4-(*o*-Tolyl)butan-2-yl)(trifluoromethyl)selane (**3k**)

Yellow oil, 30.2 mg (51%, **1k**:[Me_4_N][SeCF_3_]:TfOH = 2:2:1) and 34.3 mg (58%, **1k**:[Me_4_N][SeCF_3_]:TfOH = 1:1.5:1), petroleum ether as eluent for column chromatography. ^1^H NMR (500 MHz, CDCl_3_) δ 7.16–7.14 (m, 4H), 3.60 (m, 1H), 2.82–2.70 (m, 2H), 2.32 (s, 3H), 2.06–1.92 (m, 2H), 1.67 (d, *J* = 6.9 Hz, 3H). ^19^F NMR (471 MHz, CDCl_3_) δ −32.1 (s, 3F). ^13^C NMR (126 MHz, CDCl_3_) δ 139.1, 135.8, 130.4, 128.8, 126.3, 126.1, 123.1 (q, *J* = 330.2 Hz), 39.8, 38.1, 31.2, 23.0, 19.2. IR (KBr): 3066, 3018, 2958, 2928, 2869, 1605, 1493, 1459, 1382, 1265, 1222, 1098, 1012, 754, 739 cm^−1^. HRMS-EI (*m*/*z*) calcd. for C_12_H_15_F_3_^74^Se: 290.0351; found: 290.0345.

(4-(*m*-Tolyl)butan-2-yl)(trifluoromethyl)selane (**3l**)

Yellow oil, 33.7 mg (57%, **1l**:[Me_4_N][SeCF_3_]:TfOH = 2:2:1) and 26.1 mg (44%, **1l**:[Me_4_N][SeCF_3_]:TfOH = 1:1.5:1), petroleum ether as eluent for column chromatography. ^1^H NMR (500 MHz, CDCl_3_) δ 7.20 (t, *J* = 7.5 Hz, 1H), 7.04–6.99 (m, 3H), 3.54 (m, 1H), 2.79–2.69 (m, 2H), 2.35 (s, 3H), 2.12–1.96 (m, 2H), 1.64 (d, *J* = 6.9 Hz, 3H). ^19^F NMR (471 MHz, CDCl_3_) δ −32.0 (s, 3F). ^13^C NMR (126 MHz, CDCl_3_) δ 140.8, 138.1, 129.2, 128.4, 126.9, 125.4, 123.2 (q, *J* = 330.4 Hz), 39.5, 39.2, 33.6, 23.1, 21.4. IR (KBr): 3023, 2966, 2925, 2860, 1610, 1591, 1489, 1455, 1382, 1355, 1213, 1098, 882, 783, 738, 719, 699 cm^−1^. HRMS-EI (*m*/*z*) calcd. for C_12_H_15_F_3_^74^Se: 290.0351; found: 290.0357.

(4-(4-Methoxyphenyl)butan-2-yl)(trifluoromethyl)selane (**3m**)

Yellow oil, 33.7 mg (78%, **1m**:[Me_4_N][SeCF_3_]:TfOH = 2:2:1) and 26.1 mg (57%, **1m**:[Me_4_N][SeCF_3_]:TfOH = 1:1.5:1), a mixture of petroleum ether and ethyl acetate (80:1 (*v/v*)) as eluents for column chromatography. ^1^H NMR (500 MHz, CDCl_3_) δ 7.11 (d, *J* = 8.4 Hz, 2H), 6.85 (d, *J* = 8.5 Hz, 2H), 3.80 (s, 3H), 3.52 (m, 1H), 2.76–2.66 (m, 2H), 2.09–1.93 (m, 2H), 1.63 (d, *J* = 6.9 Hz, 3H). ^19^F NMR (471 MHz, CDCl_3_) δ −32.0 (s, 3F). ^13^C NMR (126 MHz, CDCl_3_) δ 158.1, 132.9, 129.3, 123.1 (q, *J* = 331.0 Hz), 114.0, 55.3, 39.4, 39.4, 32.7, 23.1. IR (KBr): 3032, 2991, 2954, 2928, 2855, 2837, 1613, 1584, 1513, 1456, 1382, 1301, 1248, 1178, 1098, 1038, 827, 809, 750, 738, 552, 519 cm^−1^. HRMS-EI (*m*/*z*) calcd. for C_15_H_21_F_3_^74^Se: 332.0820; found: 332.0816.

(4-(Naphthalen-2-yl)butan-2-yl)(trifluoromethyl)selane (**3n**)

Yellow oil, 49.8 mg (75%, **1n**:[Me_4_N][SeCF_3_]:TfOH = 2:2:1) and 34.5 mg (52%, **1n**:[Me_4_N][SeCF_3_]:TfOH = 1:1.5:1), petroleum ether as eluent for column chromatography. ^1^H NMR (500 MHz, CDCl_3_) δ 7.83–7.79 (m, 3H), 7.64 (s, 1H), 7.49–7.43 (m, 2H), 7.33 (dd, *J* = 8.4 Hz, 1.1 Hz, 1 H), 3.57 (m, 1H), 2.99–2.90 (m, 2H), 2.22–2.06 (m, 2H), 1.66 (d, *J* = 6.9 Hz, 3H). ^19^F NMR (471 MHz, CDCl_3_) δ −31.9 (s, 3F). ^13^C NMR (126 MHz, CDCl_3_) δ 138.3, 133.6, 132.1, 128.1, 127.6, 127.4, 127.0, 126.6, 126.1, 125.4, 123.2 (q, *J* = 330.9 Hz), 39.4, 39.0, 33.8, 23.1. IR (KBr): 3054, 3015, 2958, 2924, 2853, 1633, 1601, 1509, 1454, 1382, 1261, 1242, 1201, 1097, 1019, 960, 888, 853, 817, 746, 738 cm^−1^. HRMS-EI (*m*/*z*) calcd. for C_15_H_15_F_3_^74^Se: 326.0351; found: 326.0352.

(4-Phenylbutan-2-yl)(trifluoromethyl)selane (**3o**). 

Yellow oil, 23.1 mg (41%, **1o**:[Me_4_N][SeCF_3_]:TfOH = 2:2:1) and 11.3 mg (20%, **1o**:[Me_4_N][SeCF_3_]:TfOH = 1:1.5:1), petroleum ether as eluent for column chromatography. ^1^H NMR (500 MHz, CDCl_3_) δ 7.30 (t, *J* = 7.6 Hz, 2H), 7.23–7.19 (m, 3H), 3.53 (m, 1H), 2.82–2.73 (m, 2H), 2.13–1.97 (m, 2H), 1.63 (d, *J* = 6.9 Hz, 3H). ^19^F NMR (471 MHz, CDCl_3_) δ −32.0 (s, 3F). ^13^C NMR (126 MHz, CDCl_3_) δ 140.8, 128.5, 128.4, 126.2, 123.1 (q, *J* = 330.5 Hz), 39.4, 39.2, 33.7, 23.0 [43,46].

(6-Phenylhexan-2-yl)(trifluoromethyl)selane (**3p**)

Yellow oil, 33.5 mg (54%, **1p**:[Me_4_N][SeCF_3_]:TfOH = 2:2:1) and 21.1 mg (34%, **1p**:[Me_4_N][SeCF_3_]:TfOH = 1:1.5:1), petroleum ether as eluent for column chromatography. ^1^H NMR (500 MHz, CDCl_3_) δ 7.30 (t, *J* = 7.60 Hz, 2H), 7.21–7.18 (m, 3H), 3.55 (m, 1H), 2.64 (t, *J* = 7.7 Hz, 2H), 1.84–1.70 (m, 2H), 1.69–1.63 (m, 2H), 1.58 (d, *J* = 6.9 Hz, 3H), 1.52–1.44 (m, 2H). ^19^F NMR (471 MHz, CDCl_3_) δ −32.3 (s, 3F). ^13^C NMR (126 MHz, CDCl_3_) δ 142.3, 128.4, 128.3, 125.8, 123.2 (q, *J* = 330.8 Hz), 39.9, 37.4, 35.7, 31.0, 27.1, 22.9. IR (KBr): 3084, 3064, 3028, 2932, 2859, 1604, 1497, 1454, 1382, 1205, 1099, 1030, 909, 747, 738, 699 cm^−1^. HRMS-EI (*m*/*z*) calcd. for C_13_H_17_F_3_^74^Se: 304.0507; found: 304.0502.

### 3.3. General Procedure for Hydrotrifluoromethylthiolation of Unactivated Terminal alkenes

Under a nitrogen atmosphere, a Schlenk tube was charged with **1** (0.4 or 0.2 mmol) and CH_2_Cl_2_ (1 mL) with stirring. A solution of TfOH (0.2 mmol) in CH_2_Cl_2_ was added, followed by addition of [Me_4_N][SCF_3_] (**2b**, 0.4 or 0.3 mmol) within 1 min. The mixture was reacted at room temperature under N_2_ for 3 h and concentrated to dryness under reduced pressure. The residue was purified by flash column chromatography on silica gel using petroleum ether or a mixture of petroleum ether and ethyl acetate as eluents to give the trifluoromethylthiolated products (**4**).

(4-([1,1′-Biphenyl]-4-yl)butan-2-yl)(trifluoromethyl)sulfane (**4a**)

Yellow oil, 29.8 mg (48%, **1a**:[Me_4_N][SCF_3_]:TfOH = 2:2:1) and 20.5 mg (33%, **1a**:[Me_4_N][SCF_3_]:TfOH = 1:1.5:1), petroleum ether as eluent for column chromatography. ^1^H NMR (500 MHz, CDCl_3_) δ 7.59 (d, *J* = 7.9 Hz, 2H), 7.54 (d, *J* = 8.1 Hz, 2H), 7.44 (t, *J* = 7.5 Hz, 2H), 7.34 (t, *J* = 7.5 Hz, 1H), 7.27 (d, *J* = 7.5 Hz, 2H), 3.35 (m, 1H), 2.86–2.77 (m, 2H), 2.05–1.93 (m, 2H), 1.50 (d, *J* = 6.9 Hz, 3H). ^19^F NMR (471 MHz, CDCl_3_) δ −38.8 (s, 3F). ^13^C NMR (126 MHz, CDCl_3_) δ 140.9, 140.0, 139.2, 131.2 (q, *J* = 305.9 Hz), 128.8, 128.8, 127.3, 127.1, 127.0, 40.7, 38.5, 32.5, 22.5. IR (KBr): 3081, 3057, 3029, 2966, 2928, 2859, 1602, 1520, 1487, 1451, 1409, 1383, 1298, 1264, 1247, 1146, 1116, 1040, 1008, 965, 912, 838, 761, 732, 697, 642, 586, 553, 510 cm^−1^. HRMS-EI (*m*/*z*) calcd. for C_17_H_17_F_3_S: 310.1003; found: 310.1006.

(4-(4-Bromophenyl)butan-2-yl)(trifluoromethyl)sulfane (**4b**)

Yellow oil, 23.7 mg (38%, **1d**:[Me_4_N][SCF_3_]:TfOH = 2:2:1) and 25.0 mg (40%, **1d**:[Me_4_N][SCF_3_]:TfOH = 1:1.5:1), petroleum ether as eluent for column chromatography. ^1^H NMR (500 MHz, CDCl_3_) δ 7.42 (d, *J* = 8.3 Hz, 2H), 7.07 (d, *J* = 8.3 Hz, 2H), 3.29 (m, 1H), 2.77–2.68 (m, 2H), 1.98–1.86 (m, 2H), 1.46 (d, *J* = 6.9 Hz, 3H). ^19^F NMR (471 MHz, CDCl_3_) δ −38.9 (s, 3F). ^13^C NMR (126 MHz, CDCl_3_) δ 139.8, 131.6, 131.1 (q, *J* = 305.9 Hz), 130.1, 120.0, 40.5, 38.3, 32.2, 22.5. IR (KBr): 3026, 2966, 2929, 2856, 1897, 1732, 1646, 1592, 1489, 1456, 1405, 1383, 1296, 1246, 1156, 1116, 1073, 1042, 1012, 898, 829, 803, 771, 756, 711, 639, 521 cm^−1^. HRMS-EI (*m*/*z*) calcd. for C_11_H_12_F_3_BrS: 311.9795; found: 311.9796.

(4-(4-Iodophenyl)butan-2-yl)(trifluoromethyl)sulfane (**4c**)

Yellow oil, 34.6 mg (48%, **1e**:[Me_4_N][SCF_3_]:TfOH = 2:2:1) and 33.8 mg (47%, **1e**:[Me_4_N][SCF_3_]:TfOH = 1:1.5:1), petroleum ether as eluent for column chromatography. ^1^H NMR (500 MHz, CDCl_3_) δ 7.62 (d, *J* = 8.1 Hz, 2H), 6.95 (d, *J* = 8.1 Hz, 2H), 3.29 (m, 1H), 2.76–2.67 (m, 1H), 1.98–1.86 (m, 2H), 1.46 (d, *J* = 6.9 Hz, 3H). ^19^F NMR (471 MHz, CDCl_3_) δ −38.9 (s, 3F). ^13^C NMR (126 MHz, CDCl_3_) δ 140.5, 137.6, 131.1 (q, *J* = 306.0 Hz), 130.5, 91.2, 40.5, 38.3, 32.3, 22.5. IR (KBr): 3068, 3021, 2965, 2928, 2857, 1898, 1640, 1588, 1486, 1455, 1402, 1383, 1354, 1297, 1280, 1235, 1148, 1115, 1062, 1041, 1007, 961, 898, 826, 801, 756, 710, 631, 519 cm^−1^. HRMS-EI (*m*/*z*) calcd. for C_11_H_12_F_3_IS: 359.9656; found: 359.9660.

(4-(4-Nitrophenyl)butan-2-yl)(trifluoromethyl)sulfane (**4d**)

Yellow oil, (26.8 mg (**1g**:[Me_4_N][SCF_3_]:TfOH = 2:2:1), 48%; 23.4 mg (**1g**:[Me_4_N][SCF_3_]:TfOH = 1:1.5:1), 42%), a mixture of petroleum ether and ethyl acetate (40:1 (*v/v*)) as eluents for column chromatography. ^1^H NMR (500 MHz, CDCl_3_) δ 8.17 (d, *J* = 8.6 Hz, 2H), 7.35 (d, *J* = 8.5 Hz, 2H), 3.30 (m, 1H), 2.94–2.83 (m, 2H), 2.01–1.94 (m, 2H), 1.49 (d, *J* = 6.9 Hz, 3H). ^19^F NMR (471 MHz, CDCl_3_) δ −38.9 (s, 3F). ^13^C NMR (126 MHz, CDCl_3_) δ 148.6, 146.7, 131.0 (q, *J* = 306.5 Hz), 129.2, 123.9, 40.5, 37.9, 32.8, 22.5. IR (KBr): 3080, 2928, 2855, 2447, 1916, 1687, 1601, 1520, 1495, 1456, 1384, 1347, 1295, 1112, 1041, 1016, 901, 858, 848, 806, 756, 748, 698, 633, 519 cm^−1^. HRMS-EI (*m*/*z*) calcd. for C_11_H_12_F_3_NO_2_S: 279.0541; found: 279.0538.

(4-(4-(*Tert*-butyl)phenyl)butan-2-yl)(trifluoromethyl)sulfane (**4e**)

Yellow oil, 12.2 mg (21%, **1i**:[Me_4_N][SCF_3_]:TfOH = 2:2:1) and 9.3 mg (16%, **1i**:[Me_4_N][SCF_3_]:TfOH = 1:1.5:1), petroleum ether as eluent for column chromatography. ^1^H NMR (500 MHz, CDCl_3_) δ 7.32 (d, *J* = 8.2 Hz, 2H), 7.13 (d, *J* = 8.2 Hz, 2H), 3.33 (m, 1H), 2.78–2.69 (m, 2H), 1.99–1.88 (m, 2H), 1.47 (d, *J* = 6.9 Hz, 3H), 1.32 (s, 9H). ^19^F NMR (471 MHz, CDCl_3_) δ −38.9 (s, 3F). ^13^C NMR (126 MHz, CDCl_3_) δ 149.0, 137.8, 131.2 (q, *J* = 306.3 Hz), 128.0, 125.4, 40.8, 38.5, 34.4, 32.3, 31.4, 22.4. IR (KBr): 3057, 3025, 2965, 2929, 2858, 1645, 1512, 1461, 1415, 1395, 1382, 1364, 1269, 1150, 1116, 1043, 1020, 900, 829, 814, 756, 645, 570 cm^−1^. HRMS-ESI (*m*/*z*) calcd. for C_15_H_22_F_3_S ([M + H]^+^): 291.1380; found: 291.1389.

(4-(*p*-Tolyl)butan-2-yl)(trifluoromethyl)sulfane (**4f**)

Yellow oil, (9.4 mg (**1j**:[Me_4_N][SCF_3_]:TfOH = 2:2:1), 19%; 7.9 mg (**1j**:[Me_4_N][SCF_3_]:TfOH = 1:1.5:1), 16%), petroleum ether as eluents for column chromatography. ^1^H NMR (500 MHz, CDCl_3_) δ 7.11 (d, *J* = 8.0 Hz, 2H), 7.08 (d, *J* = 8.0 Hz, 2H), 3.31 (m, 1H), 2.77–2.68 (m, 2H), 2.33 (s, 3H), 1.99–1.86 (m, 2H), 1.46 (d, *J* = 6.9 Hz, 3H). ^19^F NMR (471 MHz, CDCl_3_) δ −38.9 (s, 3F). ^13^C NMR (126 MHz, CDCl_3_) δ 137.7, 135.7, 131.2 (q, *J* = 306.5 Hz), 129.2, 128.3, 40.6, 38.6, 32.4, 22.5, 21.0. IR (KBr): 3191, 2961, 2924, 2853, 1739, 1660, 1632, 1516, 1464, 1411, 1378, 1261, 1100, 1020, 865, 800, 756, 703 cm^−1^. HRMS-EI (*m*/*z*) calcd. for C_12_H_15_F_3_S: 248.0847; found: 248.0841.

(4-(Naphthalen-2-yl)butan-2-yl)(trifluoromethyl)sulfane (**4g**)

Yellow oil, 26.1 mg (46%, **1n**:[Me_4_N][SCF_3_]:TfOH = 2:2:1) and 22.7 mg (40%, **1n**:[Me_4_N][SCF_3_]:TfOH = 1:1.5:1), petroleum ether as eluents for column chromatography. ^1^H NMR (500 MHz, CDCl_3_) δ 7.83–7.79 (m, 3H), 7.64 (s, 1H), 7.49–7.43 (m, 2H), 7.34 (dd, *J* = 8.4, 1.6 Hz, 1H), 3.35 (m, 1H), 2.99–2.89 (m, 2H), 2.11–1.98 (m, 2H), 1.50 (d, *J* = 6.9 Hz, 3H). ^19^F NMR (471 MHz, CDCl_3_) δ −38.8 (s, 3F). ^13^C NMR (126 MHz, CDCl_3_) δ 138.3, 133.6, 132.1, 131.2 (q, *J* = 306.5 Hz), 128.2, 127.7, 127.5, 127.0, 126.6, 126.1, 125.4, 40.7, 38.4, 33.0, 22.5. IR (KBr): 3055, 3021, 2927, 2855, 1635, 1601, 1509, 1455, 1382, 1351, 1298, 1271, 1247, 1146, 1117, 1044, 1019, 961, 910, 889, 853, 817, 747 cm^−1^. HRMS-EI (*m*/*z*) calcd. for C_12_H_15_F_3_S: 284.0847; found: 284.0841.

## 4. Conclusions

In summary, we have developed a convenient method for Markovnikov-type hydrotrifluoromethylselenolation of aliphatic terminal alkenes with [Me_4_N][SeCF_3_] and TfOH. The reaction proceeded smoothly at room temperature and furnished the branched trifluoromethylselenolated products in good yields. This protocol was also applicable to the Markovnikov-type hydrotrifluoromethylthiolation of unactivated terminal alkenes using [Me_4_N][SCF_3_] and TfOH, which exhibited relatively poorer reactivity than that of [Me_4_N][SeCF_3_] under the two standard conditions. Nevertheless, reactions of alkene with CsOCF_3_ and TfOH under the same conditions did not form the hydrotrifluoromethoxylated product. The successful hydrotrifluoromethylselenolation and hydrotrifluoromethylthiolation reactions featured simplicity, convenience, and high regioselectivity, taming the sensitive ^−^XCF_3_ (X = Se, S) anions with TfOH, and represented the first synthesis of Markovnikov-type alkyl trifluoromethyl selenoethers and thioethers from unactivated terminal alkenes. Application of [Me_4_N][SeCF_3_] as a viable SeCF_3_ source for bifunctionalization of simple alkenes is currently under way in our laboratory.

## Figures and Tables

**Table 1:**
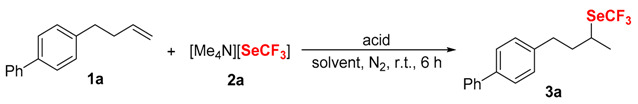
Hydrotrifluoromethylselenolation of **1a** with [Me_4_N][SeCF_3_] and an acid.

Entry ^a^	Solvent	Acid	1a:2a:Acid	Time (h)	Yields (3a, %) ^b^
1	CH_2_Cl_2_	TfOH	1:1.5:1.5	6	45
2	CH_2_Cl_2_	CF_3_COOH	1:1.5:1.5	6	0
3	CH_2_Cl_2_	conc. H_2_SO_4_	1:1.5:1.5	6	0
4	CH_2_Cl_2_	anhydrous HCl	1:1.5:1	3	0
5	CH_2_Cl_2_	H_3_PO_4_ (85%)	1:1.5:1.5	6	0
6	CH_2_Cl_2_	Et_3_N•3HF	1:1.5:1.5	6	0
7	CH_2_Cl_2_	Et_2_O•HBF_4_ (85%)	1:1.5:1.5	6	0
8	CH_2_Cl_2_	TsOH•H_2_O	1:1.5:1.5	6	0
9	CH_2_Cl_2_	(CF_3_SO_2_)_2_NH	1:1.5:1.5	6	0
10	ClCH_2_CH_2_Cl	TfOH	1:1.5:1.5	6	33
11	toluene	TfOH	1:1.5:1.5	6	19
12	PhCl	TfOH	1:1.5:1.5	6	37
13	CF_2_ClCFCl_2_	TfOH	1:1.5:1.5	6	24
14	MeCN	TfOH	1:1.5:1.5	6	0
15	(CF_3_)_2_CHOH	TfOH	1:1.5:1.5	6	0
16	CH_2_Cl_2_	TfOH	1:1.5:1.5	3	48
17	CH_2_Cl_2_	TfOH	1:1.5:1	3	71 (68)
18	CH_2_Cl_2_	TfOH	1:1.5:2	6	15
19	CH_2_Cl_2_	TfOH	1:2:1	3	57
20	CH_2_Cl_2_	TfOH	1:1:1	3	39
21	CH_2_Cl_2_	TfOH	1.5:1:1	3	42
22	CH_2_Cl_2_	TfOH	2:2:1	3	83 (79)

^a^ Reaction conditions: To a solution of **1a** (0.2, 0.3, or 0.4 mmol) in CH_2_Cl_2_ (1 mL) was added a solution of TfOH (0.2, 0.3, or 0.4 mmol) in CH_2_Cl_2_ (1 mL), followed by addition of [Me_4_N][SeCF_3_] (0.2, 0.3, or 0.4 mmol) within 1 min, at room temperature under N_2_. ^b^ The yields were determined by HPLC using pure (4-([1,1′-biphenyl]-4-yl)butan-2-yl)(trifluoromethyl)selane (**3a**) as an external standard (t_R_ = 11.85 min, λ_max_ = 253 nm, water/methanol (*v/v* = 10:90)). Isolated yield is depicted in the parenthesis.

**Table 2:**
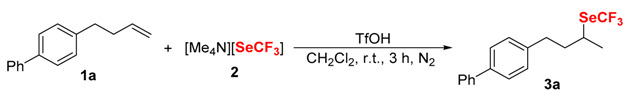
The effects of charging sequence on the reaction.

	Method A	Method B	Method C
Yield (**3a**, %) ^a^	83 ^c^, 65 ^d^	47 ^c^, 40 ^d^	0 ^c^, 0 ^d^
Yield (**3a**, %) ^b^	71 ^c^, 11 ^d^	45 ^c^, 42 ^d^	0 ^c^, 0 ^d^

^a^ Reaction conditions: **1a** (0.4 mmol), [Me_4_N][SeCF_3_] (0.4 mmol), TfOH (0.2 mmol), CH_2_Cl_2_ (2 mL), rt, 3 h, N_2_. The yields were determined by HPLC using **3a** as an external standard (t_R_ = 11.85 min, λ_max_ = 253 nm, water/methanol (*v/v* = 10:90)). ^b^ Reaction conditions: **1a** (0.2 mmol), [Me_4_N][SeCF_3_] (0.3 mmol), TfOH (0.2 mmol), CH_2_Cl_2_ (2 mL), rt, 3 h, N_2_. The yields were determined by HPLC using **3a** as an external standard (t_R_ = 11.85 min, λ_max_ = 253 nm, water/methanol (*v/v* = 10:90)). Method **A**: To a solution of **1a** and TfOH in CH_2_Cl_2_ (2 mL) was added [Me_4_N][SeCF_3_]. Method **B**: To a mixture of **1a** and [Me_4_N][SeCF_3_] in CH_2_Cl_2_ (1 mL) was added a solution of TfOH in CH_2_Cl_2_ (1 mL). Method **C**: To a mixture of [Me_4_N][SeCF_3_] and TfOH in CH_2_Cl_2_ (1 mL) was added a solution of **1a** in CH_2_Cl_2_ (1 mL). ^c^ The two reactants were premixed for 1 min before addition of the third one. ^d^ The two reactants were premixed for 1 h before addition of the third one.

## Supplementary Materials

The brief description of screening the optimal reaction conditions, general procedures for hydrotrifluoromethylchalcogenation of unactivated terminal alkenes with [Me_4_N][XCF_3_] (X = S, Se), and TfOH, control experiments for mechanistic insights, characterization data, and NMR spectra of the products (PDF) are available online.
