# Supplementary material for: Markovnikov-Type Hydrotrifluoromethylchalcogenation of Unactivated Terminal Alkenes with [Me4N][XCF3] (X = S, Se) and TfOH
Source: Molecules. 2020 Oct 3;25(19):4535. doi: 10.3390/molecules25194535 (PMC7582815; doi:10.3390/molecules25194535)
Supplement: Supplementary file 1 [file molecules-25-04535-s001.pdf]

Markovnikov-Type Hydrotrifluoromethylchalcogenation of Unactivated Alkenes with [Me_4N][XCF_3] (X = S, Se) and TfOH

Jin Shi, Cheng-Pan Zhang*

School of Chemistry, Chemical Engineering and Life Science, Wuhan University of Technology, 205 Luoshi Road, Wuhan 430070, China.

E-mail: cpzhang@whut.edu.cn, zhangchengpan1982@hotmail.com.

Table of Content

1. General Information……………………………………………………...………..S2

2. Screening the optimal reaction conditions for hydrotrifluoromethylselenolation of alkenes with [Me_4N][$SeCF_3$] and TfOH….……………………………………….........S2

3. General procedure for hydrotrifluoromethylselenolation of alkenes……...............S6

4. General procedure for hydrotrifluoromethylthiolation of alkenes………............S13

5. Hydrotrifluoromethoxylation of alkenes with $CsOCF_3$/TfOH…………………...S16

6. The control experiments for mechanistic insights……………………………….S17

7. NMR spectra of the products..……………………………….………..........S33

1. General Information

All reactions were carried out under a nitrogen atmosphere unless otherwise specified. The NMR spectra were recorded in $CDCl_3$ on a 500 MHz (for ^{1}H), 471 (for ^{19}F), and 126 (for ^{13}C) spectrometer. All chemical shifts were reported in ppm relative to TMS (0 ppm) for ^{1}H NMR and $PhOCF_3$ (58.0 ppm) or $PhCF_3$ (63.0 ppm) for ^{19}F NMR as an internal or external standard. The coupling constants were reported in Hertz (Hz). The following abbreviations were used to explain the multiplicities: s = singlet, d = doublet, t = triplet, q = quartet, m = multiplet. The HPLC experiments were carried out on a Wufeng LC-100 II instrument (column: Shodex, C18, 5 µm, 4.6 × 250 mm), and the HPLC yields of the product were determined by using the corresponding pure compound as the external standard. MS experiments were performed on a TOF-Q ESI or EI instrument. Reagents $[Me_4N][SeCF_3]$ (**2a**), $[Me_4N][SCF_3]$ (**2b**), and $CsOCF_3$ were synthesized according to the literatures.[1] Substrates **1a-f**,[2] **1g-1h**,[3] **1i-1l**,[2] **1m-1n**,[4] and **1p-1r**[4] were synthesized according to the literatures. Solvents were dried before use according to the literature.[5] Other reagents in the reactions were all purchased from the commercial sources and used without further purification.

2. Screening the optimal reaction conditions for hydrotrifluoromethylselenolation of alkenes with $[Me_4N][SeCF_3]$ and TfOH

Table S1. Reactions of **1a** and **2a** in the presence of different acids.[a]

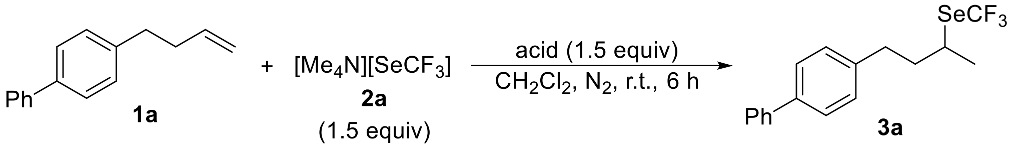

Entry	Acid	Yield (**3a**, %) [b]
1	CH_3COOH	0, 0 [c]
2	CF_3COOH	0, 0 [c]
3	conc. H_2SO_4	0
4	conc. HCl	0
5	anhydrous HCl in 1,4-dioxane	0 [d]
6	aq. H_3PO_4 (85%)	0, 0 [c]
7	$Et_3N·3HF$	0, 0 [c]
8	HBF_4 (85%) in diethyl ether	0, 0 [c]
9	$TsOH·H_2O$	0, 0 [c]

10	$(CF_3SO_2)_2NH$	0, 0 [c]
11	(1*R*)-(-)-10-camphorsulfonic acid	0
12	TfOH	45

[a] Reaction conditions: To a solution of **1a** (0.2 mmol) in CH_2Cl_2 (1 mL) was added a solution of TfOH (0.3 mmol) in CH_2Cl_2 (1 mL) followed by addition of $[Me_4N][SeCF_3]$ (0.3 mmol) within 1 minute. The mixture was reacted at room temperature under N_2 for 6 h. [b] The yields were determined by HPLC using pure (4-([1,1'-biphenyl]-4-yl)butan-2-yl)(trifluoromethyl)selane (**3a**) as an external standard (t_R = 11.85 min, λ_{max} = 253 nm, water/methanol (v/v = 10:90)). [c] 40 ^oC. [d] **1a** (0.2 mmol), acid (0.2 mmol), $[Me_4N][SeCF_3]$ (0.3 mmol), 3 h.

Table S2. Reactions of **1a** and **2a** with TfOH in different solvents.[a]

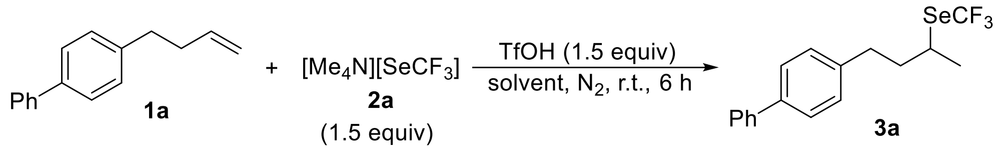

Entry	Solvent	Yield (**3a**, %) [b]
1	$CHCl_3$	0
2	$ClCH_2CH_2Cl$	33
3	CH_2Cl_2	45
4	MeCN	0
5	toluene	19
6	PhCl	37
7	$(CF_3)_2CH_2OH$	0
8	$CF_2ClCFCl_2$	24

[a] Reaction conditions: To a solution of **1a** (0.2 mmol) in solvent (1 mL) was added a solution of TfOH (0.3 mmol) in solvent (1 mL) followed by addition of $[Me_4N][SeCF_3]$ (0.3 mmol) within 1 minute. The mixture was reacted at room temperature under N_2 for 6 h. [b] The yields were determined by HPLC using pure (4-([1,1'-biphenyl]-4-yl)butan-2-yl)(trifluoromethyl)selane (**3a**) as an external standard (t_R = 11.85 min, λ_{max} = 253 nm, water/methanol (v/v = 10:90)).

Table S3. Reactions of **1a** and **2a** with TfOH at different reaction temperatures.[a]

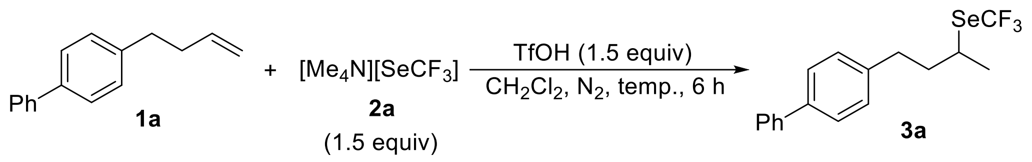

Entry	Temp (°C)	Yield (**3a**, %) [b]
1	0	43
2	r.t.	45
3	40	50
4	60	26

[a] Reaction conditions: To a solution of **1a** (0.2 mmol) in CH_2Cl_2 (1 mL) was added a solution of TfOH (0.3 mmol) in CH_2Cl_2 (1 mL) followed by addition of $[Me_4N][SeCF_3]$ (0.3 mmol) within 1 minute. The mixture was reacted under N_2 for 6 h. [b] The yields were determined by HPLC using pure (4-([1,1'-biphenyl]-4-yl)butan-2-yl)(trifluoromethyl)selane (**3a**) as an external standard (t_R = 11.85 min, λ_{max} = 253 nm, water/methanol (v/v = 10:90)).

Table S4. Reactions of **1a**, **2a** and TfOH with different reaction times.[a]

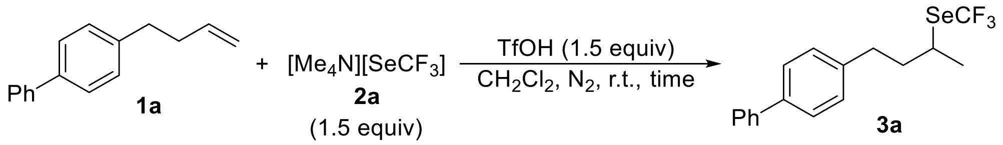

Entry	Time (h)	Yield (**3a**, %) [b]
1	3	48
2	6	45
3	12	48

[a] Reaction conditions: To a solution of **1a** (0.2 mmol) in CH_2Cl_2 (1 mL) was added a solution of TfOH (0.3 mmol) in CH_2Cl_2 (1 mL) followed by addition of $[Me_4N][SeCF_3]$ (0.3 mmol) within 1 minute. The mixture was reacted at room temperature under N_2 atmosphere. [b] The yields were determined by HPLC using pure (4-([1,1'-biphenyl]-4-yl)butan-2-yl)(trifluoromethyl)selane (**3a**) as an external standard (t_R = 11.85 min, λ_{max} = 253 nm, water/methanol (v/v = 10:90)).

Table S5. Hydrotrifluoromethylselenolation of **1a** by **2a** and TfOH with different reactant ratios.[a]

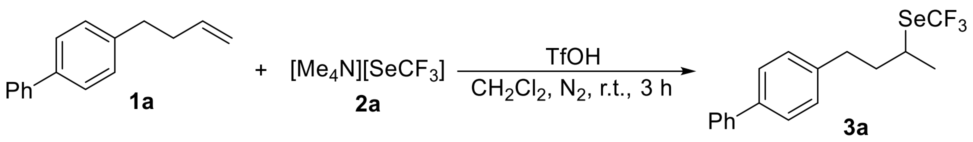

Entry	**1a** : **2** : TfOH	Yield (**3a**, %) [b]
1	1 : 1 : 1	39
2	1 : 1.5 : 1	71 (68)
3	1 : 2 : 1	57
4	1 : 2.5 : 1	66
5	1 : 1.5 : 1.5	48
6	1 : 1.5 : 2	15 [c]
7	1 : 2 : 1.5	52
8	1.5 : 1 : 1	42
9	2 : 1 : 1	62
10	2 : 1.5 : 1	70
11	2 : 2 : 1	83 (79)
12	2 : 2.5 : 1	81
13	3 : 1 : 1	65
14	3 : 1 : 1.5	57
15	3 : 1.5 : 1	73

[a] Reaction conditions: To a solution of **1a** (0.2, 0.3, 0.4 or 0.6 mmol) in CH_2Cl_2 (1 mL) was added a solution of TfOH (0.2, 0.3 or 0.4 mmol) in CH_2Cl_2 (1 mL) followed by addition of $[Me_4N][SeCF_3]$ (0.2, 0.3, 0.4 or 0.5 mmol) within 1 minute. The mixture was reacted at room temperature under N_2 for 3 h. [b] The yields were determined by HPLC using pure (4-([1,1'-biphenyl]-4-yl)butan-2-yl)(trifluoromethyl)selane (**3a**) as an external standard (t_R = 11.85 min, λ_{max} = 253 nm, water/methanol (v/v = 10:90)). Isolated yields are depicted in the parentheses. [c] 6 h.

Table S6. Hydrotrifluoromethylselenolation of **1a** by **2a** and TfOH with different metal additives. [a]

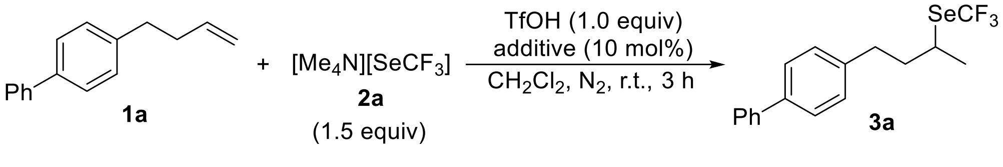

Entry	Additive	Yield (**3a**, %) [b]

1	$(Me_2S)AuCl$	51
2	CuTc	41
3	CuOTf	51
4	CuOAc	29
5	$Cu(OAc)_2$	59
6	$CuBr_2$	73, 74 [c], 38 [d]
7	$AgNO_3$	67
8	Ag_2CO_3	30
9	AgTFA	47
10	$AgPF_6$	55
11	$AgSbF_6$	51
12	AgOTf	63
13	Ag_2O	21

[a] Reaction conditions: To a mixture of additive (10 mol%) and **1a** (0.2 mmol) in CH_2Cl_2 (1 mL) was added a solution of TfOH (0.2 mmol) in CH_2Cl_2 (1 mL) followed by addition of $[Me_4N][SeCF_3]$ (0.3 mmol) within 1 minute. The mixture was reacted at room temperature under N_2 for 3 h. [b] The yields were determined by HPLC using pure (4-([1,1'-biphenyl]-4-yl)butan-2-yl)(trifluoromethyl)selane (**3a**) as an external standard (t_R = 11.85 min, λ_{max} = 253 nm, water/methanol (v/v = 10:90)). [c] $CuBr_2$ (5 mol%). [d] $CuBr_2$ (20 mol%).

3. General procedure for hydrotrifluoromethylselenolation of alkenes

Under a nitrogen atmosphere, a Schlenk tube was charged with **1** (0.4 or 0.2 mmol) and CH_2Cl_2 (1 mL) with stirring. A solution of TfOH (0.2 mmol) in CH_2Cl_2 was added, followed by addition of $[Me_4N][SeCF_3]$ (**2a**, 0.4 or 0.3 mmol) within 1 minute. The mixture was reacted at room temperature under N_2 for 3 h and concentrated to dryness under reduced pressure. The residue was purified by flash column chromatography on silica gel using petroleum ether or a mixture of petroleum ether and ethyl acetate as eluents to give the trifluoromethylselenolated products (**3**).

(4-([1,1'-Biphenyl]-4-yl)butan-2-yl)(trifluoromethyl)selane (**3a)**

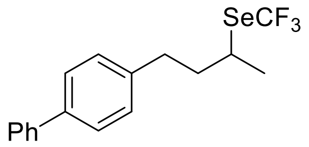

Yellow oil, 59.4 mg (79%, **1a** : [Me_4N][$SeCF_3$] : TfOH = 2 : 2 : 1) and 50.8 mg (68%, **1a** : [Me_4N][$SeCF_3$] : TfOH = 1 : 1.5 : 1), petroleum ether as eluent for column chromatography. ^{1}H NMR (500 MHz, $CDCl_3$) δ 7.62 (d, *J* = 8.1 Hz, 2H), 7.57 (d, *J* = 7.8 Hz, 2H), 7.47 (t, *J* = 7.7 Hz, 2H), 7.37 (t, *J* = 7.9 Hz, 1H), 7.30 (d, *J* = 8.0 Hz, 2H), 3.59 (m, 1H), 2.89-2.79 (m, 2H), 2.19-2.03 (m, 2H), 1.68 (d, *J* = 6.9 Hz, 3H). ^{19}F NMR (471 MHz, $CDCl_3$) δ -31.9 (s, 3F). ^{13}C NMR (126 MHz, $CDCl_3$) δ 141.0, 140.0, 139.2, 128.9, 128.8, 127.3, 127.2, 127.0, 123.2 (q, *J* = 331.1 Hz), 39.5, 39.2, 33.4, 23.1. IR (KBr): 3085, 3057, 3029, 2958, 2926, 2855, 1601, 1564, 1520, 1487, 1450, 1409, 1383, 1260, 1212, 1098, 1008, 838, 761, 738, 697, 598, 550, 507 cm^{-1}. HRMS-ESI (m/z) calcd. for $C_{17}H_{18}F_3Se$ ([M + H]$^+$): 359.0520; found: 359.0511.

(4-(4-Fluorophenyl)butan-2-yl)(trifluoromethyl)selane (**3b**)

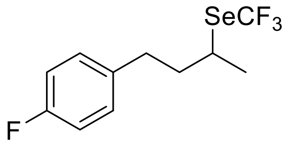

Yellow oil, 25.8 mg (43%, **1b** : [Me_4N][$SeCF_3$] : TfOH = 2 : 2 : 1) and 23.4 mg (39%, **1b** : [Me_4N][$SeCF_3$] : TfOH = 1 : 1.5 : 1), petroleum ether as eluent for column chromatography. ^{1}H NMR (500 MHz, $CDCl_3$) δ 7.42 (d, *J* = 8.3 Hz, 2H), 7.07 (d, *J* = 8.3 Hz, 2H), 3.50 (m, 1H), 2.78-2.68 (m, 2H), 2.09-1.93 (m, 2H), 1.62 (d, *J* = 7.0 Hz, 3H). ^{19}F NMR (471 MHz, $CDCl_3$) δ -32.0 (s, 3F), -117.2 (m, 1F). ^{13}C NMR (126 MHz, $CDCl_3$) δ 161.5 (d, *J* = 244.5 Hz), 136.4 (d, *J* = 3.3 Hz), 129.7 (d, *J* = 7.8 Hz), 123.1 (q, *J* = 330.8 Hz), 115.3 (d, *J* = 21.1 Hz), 39.3, 39.2, 32.9, 23.0. IR (KBr): 3041, 2927, 2856, 1602, 1511, 1455, 1383, 1259, 1224, 1157, 1098, 1016, 827, 760, 738, 543 cm^{-1}. HRMS-EI (m/z) calcd. for $C_{11}H_{12}F_4{}^{74}Se$: 294.0100; found: 294.0109.

(4-(4-Chlorophenyl)butan-2-yl)(trifluoromethyl)selane (**3c**)

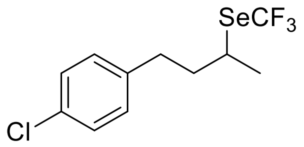

Yellow oil, 49.3 mg (78%, **1c** : [Me_4N][$SeCF_3$] : TfOH = 2 : 2 : 1) and 36.0 mg (57%,

1c : [Me_4N][$SeCF_3$] : TfOH = 1 : 1.5 : 1), petroleum ether as eluent for column chromatography. ^{1}H NMR (500 MHz, $CDCl_3$) δ 7.26 (d, *J* = 8.4 Hz, 2H), 7.12 (d, *J* = 8.3 Hz, 2H), 3.50 (m, 1H), 2.79-2.69 (m, 2H), 2.09-1.94 (m, 2H), 1.62 (d, *J* = 6.9 Hz, 3H). ^{19}F NMR (471 MHz, $CDCl_3$) δ -32.0 (s, 3F). ^{13}C NMR (126 MHz, $CDCl_3$) δ 139.3, 132.0, 129.7, 128.7, 123.1 (q, *J* = 331.3 Hz), 39.2, 39.1, 33.0, 23.0. IR (KBr): 3084, 3028, 2927, 2856, 1895, 1731, 1598, 1493, 1455, 1408, 1383, 1290, 1279, 1260, 1232, 1214, 1096, 1035, 1016, 832, 818, 807, 778, 738, 715, 672, 663, 631, 523 cm^{-1}. HRMS-ESI (m/z) calcd. for $C_{10}H_{12}Cl$ ([M – $SeCF_3$]$^+$): 167.0622; found: 167.0624.

(4-(4-Bromophenyl)butan-2-yl)(trifluoromethyl)selane (**3d**)

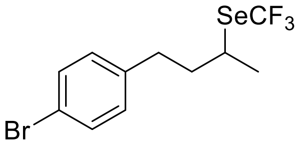

Yellow oil, 58.3 mg (81%, **1d** : [Me_4N][$SeCF_3$] : TfOH = 2 : 2 : 1) and 43.2 mg (60%, **1d** : [Me_4N][$SeCF_3$] : TfOH = 1 : 1.5 : 1), petroleum ether as eluent for column chromatography. ^{1}H NMR (500 MHz, $CDCl_3$) δ 7.42 (d, *J* = 8.3 Hz, 2H), 7.07 (d, *J* = 8.3 Hz, 2H), 3.50 (m, 1H), 2.78-2.68 (m, 2H), 2.09-1.93 (m, 2H), 1.62 (d, *J* = 6.9 Hz, 3H). ^{19}F NMR (471 MHz, $CDCl_3$) δ -32.0 (s, 3F). ^{13}C NMR (126 MHz, $CDCl_3$) δ 139.8, 131.6, 130.1, 123.1 (q, *J* = 331.0 Hz), 120.0, 39.2, 39.0, 33.1, 23.0. IR (KBr): 3025, 2961, 2926, 2863, 1489, 1454, 1405, 1383, 1278, 1260, 1214, 1097, 1074, 1012, 960, 897, 828, 813, 802, 770, 738, 711, 654, 634, 605, 515 cm^{-1}. HRMS-EI (m/z) calcd. for $C_{11}H_{12}F_3Br^{74}Se$: 353.9299; found: 353.9307.

(4-(4-Iodophenyl)butan-2-yl)(trifluoromethyl)selane (**3e**)

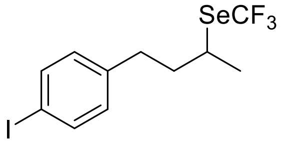

Yellow oil, 69.3 mg (85%, **1e** : [Me_4N][$SeCF_3$] : TfOH = 2 : 2 : 1) and 43.2 mg (53%, **1e** : [Me_4N][$SeCF_3$] : TfOH = 1 : 1.5 : 1), petroleum ether as eluent for column chromatography. ^{1}H NMR (500 MHz, $CDCl_3$) δ 7.62 (d, *J* = 8.3 Hz, 2H), 6.95 (d, *J* = 8.3 Hz, 2H), 3.49 (m, 1H), 2.76-2.67 (m, 2H), 2.08-1.93 (m, 2H), 1.62 (d, *J* = 6.9 Hz, 3H). ^{19}F NMR (471 MHz, $CDCl_3$) δ -32.0 (s, 3F). ^{13}C NMR (126 MHz, $CDCl_3$) δ 140.5, 137.6, 130.5, 123.1 (q, *J* = 331.2 Hz), 91.3, 39.2, 39.0, 33.2, 23.0. IR (KBr): 3019, 2960, 2925, 2854, 1485, 1454, 1401, 1382, 1291, 1275, 1260, 1230, 1213, 1202,

1097, 1063, 1035, 1007, 897, 826, 799, 766, 738, 709, 512 cm^{-1}. HRMS-ESI (m/z) calcd. for $C_9H_{12}ISe$ ([M – CF_3]$^-$): 337.9196; found: 337.9198.

(Trifluoromethyl)(4-(4-(trifluoromethyl)phenyl)butan-2-yl)selane (**3f**)

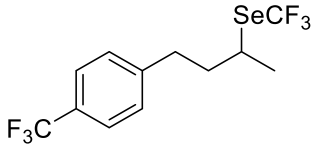

Yellow oil, 42.0 mg (60%, **1f** : [Me_4N][$SeCF_3$] : TfOH = 2 : 2 : 1) and 30.1 mg (43%, **1f** : [Me_4N][$SeCF_3$] : TfOH = 1 : 1.5 : 1), petroleum ether as eluent for column chromatography. ^{1}H NMR (500 MHz, $CDCl_3$) δ 7.56 (d, *J* = 8.1 Hz, 2H), 7.31 (d, *J* = 8.0 Hz, 2H), 3.51 (m, 1H), 2.89-2.79 (m, 2H), 2.13-1.98 (m, 2H), 1.64 (d, *J* = 6.9 Hz, 3H). ^{19}F NMR (471 MHz, $CDCl_3$) δ -32.0 (s, 3F), -62.4 (s, 3F). ^{13}C NMR (126 MHz, $CDCl_3$) δ 145.0, 128.7, 128.6 (q, *J* = 32.4 Hz), 125.5 (q, *J* = 3.8 Hz) 124.3 (q, *J* = 271.5 Hz), 123.1 (q, *J* = 330.8 Hz), 39.2, 38.9, 33.5, 23.0. IR (KBr): 2958, 2929, 2858, 1620, 1456, 1419, 1384, 1327, 1165, 1117, 1095, 1068, 1019, 899, 840, 823, 738, 650, 633, 599 cm^{-1}. HRMS-EI (m/z) calcd. for $C_{12}H_{12}F_6{}^{74}Se$: 344.0068; found: 344.0061.

(4-(4-Nitrophenyl)butan-2-yl)(trifluoromethyl)selane (**3g**)

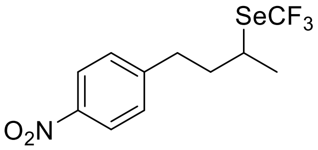

Yellow oil, 46.4 mg (71%, **1g** : [Me_4N][$SeCF_3$] : TfOH = 2 : 2 : 1) and 28.1 mg (43%, **1g** : [Me_4N][$SeCF_3$] : TfOH = 1 : 1.5 : 1), a mixture of petroleum ether and ethyl acetate (40 : 1 (v/v)) as eluents for column chromatography. ^{1}H NMR (500 MHz, $CDCl_3$) δ 8.17 (d, *J* = 8.4 Hz, 2H), 7.36 (d, *J* = 8.4 Hz, 2H), 3.50 (m, 1H), 2.94-2.84 (m, 2H), 2.14-2.00 (m, 2H), 1.64 (d, *J* = 6.9 Hz, 3H). ^{19}F NMR (471 MHz, $CDCl_3$) δ -32.0 (s, 3F). ^{13}C NMR (126 MHz, $CDCl_3$) δ 148.7, 146.7, 129.2, 123.9, 123.0 (q, *J* = 331.1 Hz), 39.0, 38.7, 33.6, 23.0. IR (KBr): 3112, 3080, 2950, 2929, 2856, 2453, 2217, 1927, 1727, 1602, 1520, 1495, 1455, 1384, 1347, 1319, 1289, 1236, 1214, 1180, 1098, 1035, 1016, 973, 891, 858, 848, 806, 768, 747, 738, 698, 660, 647, 632, 619, 513 cm^{-1}. HRMS-ESI (m/z) calcd. for $C_{11}H_{13}F_3NO_2Se$ ([M + H]$^+$): 328.0058; found: 328.0060.

(4-(2-Nitrophenyl)butan-2-yl)(trifluoromethyl)selane (**3h**)

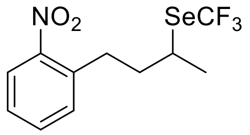

Yellow oil, 37.3 mg (57%, **1h** : [Me_4N][$SeCF_3$] : TfOH = 2 : 2 : 1) and 21.6 mg (33%, **1h** : [Me_4N][$SeCF_3$] : TfOH = 1 : 1.5 : 1), a mixture of petroleum ether and ethyl acetate (40 : 1 (v/v)) as eluents for column chromatography. ^{1}H NMR (500 MHz, $CDCl_3$) δ 7.94 (d, *J* = 8.1 Hz, 1H), 7.55 (t, *J* = 7.6 Hz, 1H), 7.40-7.36 (m, 2H), 3.60 (m, 1H), 3.11-2.98 (m, 2H), 2.14-2.03 (m, 2H), 1.66 (d, *J* = 7.0 Hz, 3H). ^{19}F NMR (471 MHz, $CDCl_3$) δ -32.1 (s, 3F). ^{13}C NMR (126 MHz, $CDCl_3$) δ 149.2, 136.1, 133.2, 132.0, 127.5, 125.0, 123.1 (q, *J* = 330.5 Hz), 39.5, 38.5, 31.3, 22.8. IR (KBr): 3069, 2964, 2928, 2856, 1611, 1579, 1527, 1481, 1457, 1383, 1348, 1281, 1231, 1215, 1099, 959, 861, 813, 787, 739, 702, 667 cm^{-1}. HRMS-EI (m/z) calcd. for $C_{11}H_{12}F_3NO_2{}^{74}Se$: 321.0045; found: 321.0039.

(4-(4-(*Tert*-butyl)phenyl)butan-2-yl)(trifluoromethyl)selane (**3i**)

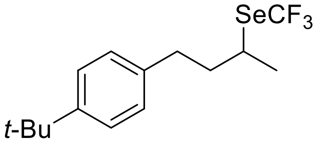

Yellow oil, 45.3 mg (67%, **1i** : [Me_4N][$SeCF_3$] : TfOH = 2 : 2 : 1) and 33.8 mg (50%, **1i** : [Me_4N][$SeCF_3$] : TfOH = 1 : 1.5 : 1), petroleum ether as eluent for column chromatography. ^{1}H NMR (500 MHz, $CDCl_3$) δ 7.33 (d, *J* = 8.1 Hz, 2H), 7.13 (d, *J* = 8.1 Hz, 2H), 3.55 (m, 1H), 2.79-2.69 (m, 2H), 2.12-1.96 (m, 2H), 1.64 (d, *J* = 6.9 Hz, 3H), 1.32 (s, 9H). ^{19}F NMR (471 MHz, $CDCl_3$) δ -32.0 (s, 3F). ^{13}C NMR (126 MHz, $CDCl_3$) δ 149.0, 137.8, 128.0, 125.4, 123.2 (q, *J* = 331.2 Hz), 39.5, 39.2, 34.4, 33.1, 31.4, 23.0. IR (KBr): 3056, 3024, 2964, 2868, 1517, 1457, 1412, 1394, 1382, 1364, 1269, 1234, 1215, 1203, 1098, 1035, 1019, 829, 815, 738, 569 cm^{-1}. HRMS-EI (m/z) calcd. for $C_{15}H_{21}F_3{}^{74}Se$: 332.0820; found: 332.0816.

(4-(*p*-Tolyl)butan-2-yl)(trifluoromethyl)selane (**3j**)

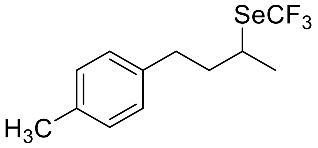

Yellow oil, 46.2 mg (78%, **1j** : [Me_4N][$SeCF_3$] : TfOH = 2 : 2 : 1) and 29.6 mg (50%, **1j** : [Me_4N][$SeCF_3$] : TfOH = 1 : 1.5 : 1), petroleum ether as eluent for column

chromatography. ^{1}H NMR (500 MHz, $CDCl_3$) δ 7.12-7.08 (m, 4H), 3.53 (m, 1H), 2.78-2.68 (m, 2H), 2.33 (s, 3H), 2.10-1.94 (m, 2H), 1.63 (d, *J* = 6.9 Hz, 3H). ^{19}F NMR (471 MHz, $CDCl_3$) δ -32.0 (s, 3F). ^{13}C NMR (126 MHz, $CDCl_3$) δ 137.7, 135.7, 129.2, 128.3, 123.2 (q, *J* = 330.5 Hz), 39.4, 39.3, 33.2, 23.0, 21.0. IR (KBr): 3048, 3020, 2925, 2859, 1516, 1454, 1381, 1260, 1214, 1097, 1022, 830, 807, 738, 543 cm^{-1}. HRMS-EI (m/z) calcd. for $C_{12}H_{15}F_3{}^{74}Se$: 290.0351; found: 290.0349.

(4-(*o*-Tolyl)butan-2-yl)(trifluoromethyl)selane (**3k**)

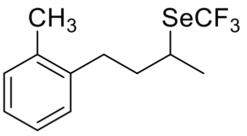

Yellow oil, 30.2 mg (51%, **1k** : [Me_4N][$SeCF_3$] : TfOH = 2 : 2 : 1) and 34.3 mg (58%, **1k** : [Me_4N][$SeCF_3$] : TfOH = 1 : 1.5 : 1), petroleum ether as eluent for column chromatography. ^{1}H NMR (500 MHz, $CDCl_3$) δ 7.16-7.14 (m, 4H), 3.60 (m, 1H), 2.82-2.70 (m, 2H), 2.32 (s, 3H), 2.06-1.92 (m, 2H), 1.67 (d, *J* = 6.9 Hz, 3H). ^{19}F NMR (471 MHz, $CDCl_3$) δ -32.1 (s, 3F). ^{13}C NMR (126 MHz, $CDCl_3$) δ 139.1, 135.8, 130.4, 128.8, 126.3, 126.1, 123.1 (q, *J* = 330.2 Hz), 39.8, 38.1, 31.2, 23.0, 19.2. IR (KBr): 3066, 3018, 2958, 2928, 2869, 1605, 1493, 1459, 1382, 1265, 1222, 1098, 1012, 754, 739 cm^{-1}. HRMS-EI (m/z) calcd. for $C_{12}H_{15}F_3{}^{74}Se$: 290.0351; found: 290.0345.

(4-(*m*-Tolyl)butan-2-yl)(trifluoromethyl)selane (**3l**)

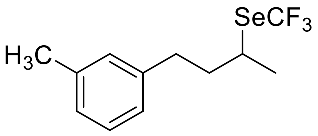

Yellow oil, 33.7 mg (57%, **1l** : [Me_4N][$SeCF_3$] : TfOH = 2 : 2 : 1) and 26.1 mg (44%, **1l** : [Me_4N][$SeCF_3$] : TfOH = 1 : 1.5 : 1), petroleum ether as eluent for column chromatography. ^{1}H NMR (500 MHz, $CDCl_3$) δ 7.20 (t, *J* = 7.5 Hz, 1H), 7.04-6.99 (m, 3H), 3.54 (m, 1H), 2.79-2.69 (m, 2H), 2.35 (s, 3H), 2.12-1.96 (m, 2H), 1.64 (d, *J* = 6.9 Hz, 3H). ^{19}F NMR (471 MHz, $CDCl_3$) δ -32.0 (s, 3F). ^{13}C NMR (126 MHz, $CDCl_3$) δ 140.8, 138.1, 129.2, 128.4, 126.9, 125.4, 123.2 (q, *J* = 330.4 Hz), 39.5, 39.2, 33.6, 23.1, 21.4. IR (KBr): 3023, 2966, 2925, 2860, 1610, 1591, 1489, 1455, 1382, 1355, 1213, 1098, 882, 783, 738, 719, 699 cm^{-1}. HRMS-EI (m/z) calcd. for $C_{12}H_{15}F_3{}^{74}Se$: 290.0351; found: 290.0357.

(4-(4-Methoxyphenyl)butan-2-yl)(trifluoromethyl)selane (**3m**)

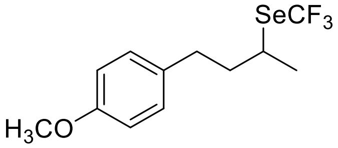

Yellow oil, 33.7 mg (78%, **1m** : [Me_4N][$SeCF_3$] : TfOH = 2 : 2 : 1) and 26.1 mg (57%, **1m** : [Me_4N][$SeCF_3$] : TfOH = 1 : 1.5 : 1), a mixture of petroleum ether and ethyl acetate (80 : 1 (v/v)) as eluents for column chromatography. ^{1}H NMR (500 MHz, $CDCl_3$) δ 7.11 (d, *J* = 8.4 Hz, 2H), 6.85 (d, *J* = 8.5 Hz, 2H), 3.80 (s, 3H), 3.52 (m, 1H), 2.76-2.66 (m, 2H), 2.09-1.93 (m, 2H), 1.63 (d, *J* = 6.9 Hz, 3H). ^{19}F NMR (471 MHz, $CDCl_3$) δ -32.0 (s, 3F). ^{13}C NMR (126 MHz, $CDCl_3$) δ 158.1, 132.9, 129.3, 123.1 (q, *J* = 331.0 Hz), 114.0, 55.3, 39.4, 39.4, 32.7, 23.1. IR (KBr): 3032, 2991, 2954, 2928, 2855, 2837, 1613, 1584, 1513, 1456, 1382, 1301, 1248, 1178, 1098, 1038, 827, 809, 750, 738, 552, 519 cm^{-1}. HRMS-EI (m/z) calcd. for $C_{15}H_{21}F_3{}^{74}Se$: 332.0820; found: 332.0816.

(4-(Naphthalen-2-yl)butan-2-yl)(trifluoromethyl)selane (**3n**)

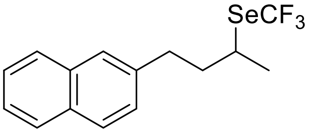

Yellow oil, 49.8 mg (75%, **1n** : [Me_4N][$SeCF_3$] : TfOH = 2 : 2 : 1) and 34.5 mg (52%, **1n** : [Me_4N][$SeCF_3$] : TfOH = 1 : 1.5 : 1), petroleum ether as eluent for column chromatography. ^{1}H NMR (500 MHz, $CDCl_3$) δ 7.83-7.79 (m, 3H), 7.64 (s, 1H), 7.49-7.43 (m, 2H), 7.33 (dd, *J* = 8.4 Hz, 1.1 Hz, 1 H), 3.57 (m, 1H), 2.99-2.90 (m, 2H), 2.22-2.06 (m, 2H), 1.66 (d, *J* = 6.9 Hz, 3H). ^{19}F NMR (471 MHz, $CDCl_3$) δ -31.9 (s, 3F). ^{13}C NMR (126 MHz, $CDCl_3$) δ 138.3, 133.6, 132.1, 128.1, 127.6, 127.4, 127.0, 126.6, 126.1, 125.4, 123.2 (q, *J* = 330.9 Hz), 39.4, 39.0, 33.8, 23.1. IR (KBr): 3054, 3015, 2958, 2924, 2853, 1633, 1601, 1509, 1454, 1382, 1261, 1242, 1201, 1097, 1019, 960, 888, 853, 817, 746, 738 cm^{-1}. HRMS-EI (m/z) calcd. for $C_{15}H_{15}F_3{}^{74}Se$: 326.0351; found: 326.0352.

(4-Phenylbutan-2-yl)(trifluoromethyl)selane (**3o**)[6]

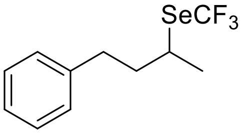

Yellow oil, 23.1 mg (41%, **1o** : $[Me_4N][SeCF_3]$: TfOH = 2 : 2 : 1) and 11.3 mg (20%, **1o** : $[Me_4N][SeCF_3]$: TfOH = 1 : 1.5 : 1), petroleum ether as eluent for column chromatography. ^{1}H NMR (500 MHz, $CDCl_3$) δ 7.30 (t, *J* = 7.6 Hz, 2H), 7.23-7.19 (m, 3H), 3.53 (m, 1H), 2.82-2.73 (m, 2H), 2.13-1.97 (m, 2H), 1.63 (d, *J* = 6.9 Hz, 3H). ^{19}F NMR (471 MHz, $CDCl_3$) δ -32.0 (s, 3F). ^{13}C NMR (126 MHz, $CDCl_3$) δ 140.8, 128.5, 128.4, 126.2, 123.1 (q, *J* = 330.5 Hz), 39.4, 39.2, 33.7, 23.0.

(6-Phenylhexan-2-yl)(trifluoromethyl)selane (**3p**)

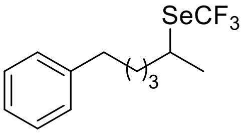

Yellow oil, 33.5 mg (54%, **1p** : $[Me_4N][SeCF_3]$: TfOH = 2 : 2 : 1) and 21.1 mg (34%, **1p** : $[Me_4N][SeCF_3]$: TfOH = 1 : 1.5 : 1), petroleum ether as eluent for column chromatography. ^{1}H NMR (500 MHz, $CDCl_3$) δ 7.30 (t, *J* = 7.60 Hz, 2H), 7.21-7.18 (m, 3H), 3.55 (m, 1H), 2.64 (t, *J* = 7.7 Hz, 2H), 1.84-1.70 (m, 2H), 1.69-1.63 (m, 2H), 1.58 (d, *J* = 6.9 Hz, 3H), 1.52-1.44 (m, 2H). ^{19}F NMR (471 MHz, $CDCl_3$) δ -32.3 (s, 3F). ^{13}C NMR (126 MHz, $CDCl_3$) δ 142.3, 128.4, 128.3, 125.8, 123.2 (q, *J* = 330.8 Hz), 39.9, 37.4, 35.7, 31.0, 27.1, 22.9. IR (KBr): 3084, 3064, 3028, 2932, 2859, 1604, 1497, 1454, 1382, 1205, 1099, 1030, 909, 747, 738, 699 cm^{-1}. HRMS-EI (m/z) calcd. for $C_{13}H_{17}F_3{}^{74}Se$: 304.0507; found: 304.0502.

4. General procedure for hydrotrifluoromethylthiolation of alkenes

Under a nitrogen atmosphere, a Schlenk tube was charged with **1** (0.4 or 0.2 mmol) and CH_2Cl_2 (1 mL) with stirring. A solution of TfOH (0.2 mmol) in CH_2Cl_2 was added, followed by addition of $[Me_4N][SCF_3]$ (**2b**, 0.4 or 0.3 mmol) within 1 minute. The mixture was reacted at room temperature under N_2 for 3 h and concentrated to dryness under reduced pressure. The residue was purified by flash column chromatography on silica gel using petroleum ether or a mixture of petroleum ether and ethyl acetate as eluents to give the trifluoromethylthiolated products (**4**).

(4-([1,1'-Biphenyl]-4-yl)butan-2-yl)(trifluoromethyl)sulfane (**4a**)

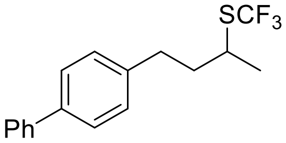

Yellow oil, 29.8 mg (48%, **1a** : [Me_4N][SCF_3] : TfOH = 2 : 2 : 1) and 20.5 mg (33%, **1a** : [Me_4N][SCF_3] : TfOH = 1 : 1.5 : 1), petroleum ether as eluent for column chromatography. ^{1}H NMR (500 MHz, $CDCl_3$) δ 7.59 (d, *J* = 7.9 Hz, 2H), 7.54 (d, *J* = 8.1 Hz, 2H), 7.44 (t, *J* = 7.5 Hz, 2H), 7.34 (t, *J* = 7.5 Hz, 1H), 7.27 (d, *J* = 7.5 Hz, 2H), 3.35 (m, 1H), 2.86-2.77 (m, 2H), 2.05-1.93 (m, 2H), 1.50 (d, *J* = 6.9 Hz, 3H). ^{19}F NMR (471 MHz, $CDCl_3$) δ -38.8 (s, 3F). ^{13}C NMR (126 MHz, $CDCl_3$) δ 140.9, 140.0, 139.2, 131.2 (q, *J* = 305.9 Hz), 128.8, 128.8, 127.3, 127.1, 127.0, 40.7, 38.5, 32.5, 22.5. IR (KBr): 3081, 3057, 3029, 2966, 2928, 2859, 1602, 1520, 1487, 1451, 1409, 1383, 1298, 1264, 1247, 1146, 1116, 1040, 1008, 965, 912, 838, 761, 732, 697, 642, 586, 553, 510 cm^{-1}. HRMS-EI (m/z) calcd. for $C_{17}H_{17}F_3S$: 310.1003; found: 310.1006.

(4-(4-Bromophenyl)butan-2-yl)(trifluoromethyl)sulfane (**4b**)

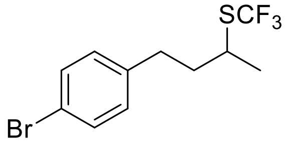

Yellow oil, 23.7 mg (38%, **1d** : [Me_4N][SCF_3] : TfOH = 2 : 2 : 1) and 25.0 mg (40%, **1d** : [Me_4N][SCF_3] : TfOH = 1 : 1.5 : 1), petroleum ether as eluent for column chromatography. ^{1}H NMR (500 MHz, $CDCl_3$) δ 7.42 (d, *J* = 8.3 Hz, 2H), 7.07 (d, *J* = 8.3 Hz, 2H), 3.29 (m, 1H), 2.77-2.68 (m, 2H), 1.98-1.86 (m, 2H), 1.46 (d, *J* = 6.9 Hz, 3H). ^{19}F NMR (471 MHz, $CDCl_3$) δ -38.9 (s, 3F). ^{13}C NMR (126 MHz, $CDCl_3$) δ 139.8, 131.6, 131.1 (q, *J* = 305.9 Hz), 130.1, 120.0, 40.5, 38.3, 32.2, 22.5. IR (KBr): 3026, 2966, 2929, 2856, 1897, 1732, 1646, 1592, 1489, 1456, 1405, 1383, 1296, 1246, 1156, 1116, 1073, 1042, 1012, 898, 829, 803, 771, 756, 711, 639, 521 cm^{-1}. HRMS-EI (m/z) calcd. for $C_{11}H_{12}F_3BrS$: 311.9795; found: 311.9796.

(4-(4-Iodophenyl)butan-2-yl)(trifluoromethyl)sulfane (**4c**)

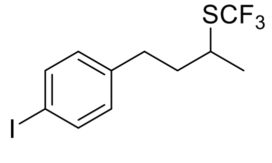

Yellow oil, 34.6 mg (48%, **1e** : [Me_4N][SCF_3] : TfOH = 2 : 2 : 1) and 33.8 mg (47%, **1e** : [Me_4N][SCF_3] : TfOH = 1 : 1.5 : 1), petroleum ether as eluent for column chromatography. ^{1}H NMR (500 MHz, $CDCl_3$) δ 7.62 (d, *J* = 8.1 Hz, 2H), 6.95 (d, *J* = 8.1 Hz, 2H), 3.29 (m, 1H), 2.76-2.67 (m, 1H), 1.98-1.86 (m, 2H), 1.46 (d, *J* = 6.9 Hz,

3H). ^{19}F NMR (471 MHz, $CDCl_3$) δ -38.9 (s, 3F). ^{13}C NMR (126 MHz, $CDCl_3$) δ 140.5, 137.6, 131.1 (q, J = 306.0 Hz), 130.5, 91.2, 40.5, 38.3, 32.3, 22.5. IR (KBr): 3068, 3021, 2965, 2928, 2857, 1898, 1640, 1588, 1486, 1455, 1402, 1383, 1354, 1297, 1280, 1235, 1148, 1115, 1062, 1041, 1007, 961, 898, 826, 801, 756, 710, 631, 519 cm^{-1}. HRMS-EI (m/z) calcd. for $C_{11}H_{12}F_3IS$: 359.9656; found: 359.9660.

(4-(4-Nitrophenyl)butan-2-yl)(trifluoromethyl)sulfane (**4d**)

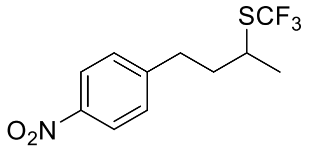

Yellow oil, (26.8 mg (**1g** : [Me_4N][SCF_3] : TfOH = 2 : 2 : 1), 48%; 23.4 mg (**1g** : [Me_4N][SCF_3] : TfOH = 1 : 1.5 : 1), 42%), a mixture of petroleum ether and ethyl acetate (40 : 1 (v/v)) as eluents for column chromatography. ^{1}H NMR (500 MHz, $CDCl_3$) δ 8.17 (d, J = 8.6 Hz, 2H), 7.35 (d, J = 8.5 Hz, 2H), 3.30 (m, 1H), 2.94-2.83 (m, 2H), 2.01-1.94 (m, 2H), 1.49 (d, J = 6.9 Hz, 3H). ^{19}F NMR (471 MHz, $CDCl_3$) δ -38.9 (s, 3F). ^{13}C NMR (126 MHz, $CDCl_3$) δ 148.6, 146.7, 131.0 (q, J = 306.5 Hz), 129.2, 123.9, 40.5, 37.9, 32.8, 22.5. IR (KBr): 3080, 2928, 2855, 2447, 1916, 1687, 1601, 1520, 1495, 1456, 1384, 1347, 1295, 1112, 1041, 1016, 901, 858, 848, 806, 756, 748, 698, 633, 519 cm^{-1}. HRMS-EI (m/z) calcd. for $C_{11}H_{12}F_3NO_2S$: 279.0541; found: 279.0538.

(4-(4-(*Tert*-butyl)phenyl)butan-2-yl)(trifluoromethyl)sulfane (**4e**)

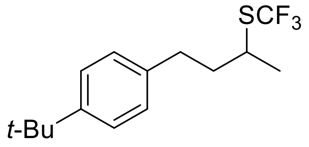

Yellow oil, 12.2 mg (21%, **1i** : [Me_4N][SCF_3] : TfOH = 2 : 2 : 1) and 9.3 mg (16%, **1i** : [Me_4N][SCF_3] : TfOH = 1 : 1.5 : 1), petroleum ether as eluent for column chromatography. ^{1}H NMR (500 MHz, $CDCl_3$) δ 7.32 (d, J = 8.2 Hz, 2H), 7.13 (d, J = 8.2 Hz, 2H), 3.33 (m, 1H), 2.78-2.69 (m, 2H), 1.99-1.88 (m, 2H), 1.47 (d, J = 6.9 Hz, 3H), 1.32 (s, 9H). ^{19}F NMR (471 MHz, $CDCl_3$) δ -38.9 (s, 3F). ^{13}C NMR (126 MHz, $CDCl_3$) δ 149.0, 137.8, 131.2 (q, J = 306.3 Hz), 128.0, 125.4, 40.8, 38.5, 34.4, 32.3, 31.4, 22.4. IR (KBr): 3057, 3025, 2965, 2929, 2858, 1645, 1512, 1461, 1415, 1395, 1382, 1364, 1269, 1150, 1116, 1043, 1020, 900, 829, 814, 756, 645, 570 cm^{-1}.

HRMS-ESI (m/z) calcd. for $C_{15}H_{22}F_3S$ ($[M + H]^+$): 291.1380; found: 291.1389.

(4-(*p*-Tolyl)butan-2-yl)(trifluoromethyl)sulfane (**4f**)

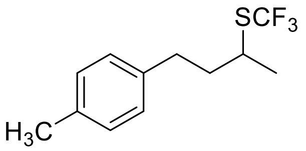

Yellow oil, (9.4 mg (**1j** : [Me_4N][SCF_3] : TfOH = 2 : 2 : 1), 19%; 7.9 mg (**1j** : [Me_4N][SCF_3] : TfOH = 1 : 1.5 : 1), 16%), petroleum ether as eluents for column chromatography. ^1H NMR (500 MHz, $CDCl_3$) δ 7.11 (d, *J* = 8.0 Hz, 2H), 7.08 (d, *J* = 8.0 Hz, 2H), 3.31 (m, 1H), 2.77-2.68 (m, 2H), 2.33 (s, 3H), 1.99-1.86 (m, 2H), 1.46 (d, *J* = 6.9 Hz, 3H). ^{19}F NMR (471 MHz, $CDCl_3$) δ -38.9 (s, 3F). ^{13}C NMR (126 MHz, $CDCl_3$) δ 137.7, 135.7, 131.2 (q, *J* = 306.5 Hz), 129.2, 128.3, 40.6, 38.6, 32.4, 22.5, 21.0. IR (KBr): 3191, 2961, 2924, 2853, 1739, 1660, 1632, 1516, 1464, 1411, 1378, 1261, 1100, 1020, 865, 800, 756, 703 cm^{-1}. HRMS-EI (m/z) calcd. for $C_{12}H_{15}F_3S$: 248.0847; found: 248.0841.

(4-(Naphthalen-2-yl)butan-2-yl)(trifluoromethyl)sulfane (**4g**)

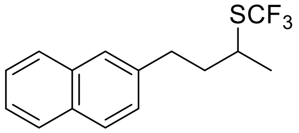

Yellow oil, 26.1 mg (46%, **1n** : [Me_4N][SCF_3] : TfOH = 2 : 2 : 1) and 22.7 mg (40%, **1n** : [Me_4N][SCF_3] : TfOH = 1 : 1.5 : 1) , petroleum ether as eluents for column chromatography. ^1H NMR (500 MHz, $CDCl_3$) δ 7.83-7.79 (m, 3H), 7.64 (s, 1H), 7.49-7.43 (m, 2H), 7.34 (dd, *J* = 8.4, 1.6 Hz, 1H), 3.35 (m, 1H), 2.99-2.89 (m, 2H), 2.11-1.98 (m, 2H), 1.50 (d, *J* = 6.9 Hz, 3H). ^{19}F NMR (471 MHz, $CDCl_3$) δ -38.8 (s, 3F). ^{13}C NMR (126 MHz, $CDCl_3$) δ 138.3, 133.6, 132.1, 131.2 (q, *J* = 306.5 Hz), 128.2, 127.7, 127.5, 127.0, 126.6, 126.1, 125.4, 40.7, 38.4, 33.0, 22.5. IR (KBr): 3055, 3021, 2927, 2855, 1635, 1601, 1509, 1455, 1382, 1351, 1298, 1271, 1247, 1146, 1117, 1044, 1019, 961, 910, 889, 853, 817, 747 cm^{-1}. HRMS-EI (m/z) calcd. for $C_{12}H_{15}F_3S$: 284.0847; found: 284.0841.

5. Hydrotrifluoromethoxylation of alkenes with $CsOCF_3$/TfOH

Procedure: Under a nitrogen atmosphere, a Schlenk tube was charged with **1a** (0.4 mmol) and CH_2Cl_2 (1 mL) with stirring. A solution of TfOH (0.2 mmol) in CH_2Cl_2

was added, followed by addition of $CsOCF_3$ (0.4 mmol) within 1 minute. The mixture was reacted at room temperature under N_2 for 3 h.

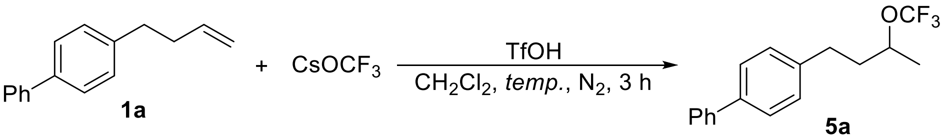

Entry [a]	Temp. (^{o}C)	Yield (6**a**, %)
1	r.t.	0, 0 [b]
2	40	0, 0 [b]
3	60	0, 0 [b]

[a] Reaction conditions: To a solution of **1a** (0.4 mmol) in CH_2Cl_2 (1 mL) was added a solution of TfOH (0.2 mmol) in CH_2Cl_2 (1 mL), followed by addition of $CsOCF_3$ (0.4 mmol) within 1 minute. The mixture was reacted at room temperature under N_2 for 3 h. The yields were determined by ^{19}F NMR using $PhCF_3$ as an internal standard. [b] The same reaction was run with **1a** (0.2 mmol), TfOH (0.2 mmol) and $CsOCF_3$ (0.3 mmol).

6. The control experiments for mechanistic insights

Experiment 1:

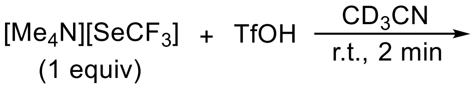

Under a nitrogen atmosphere, a sealed tube was charged with $[Me_4N][SeCF_3]$ (11.1 mg, 0.05 mmol) and a solution of TfOH (0.05 mmol) in CD_3CN (1 mL) with stirring. The mixture was maintained at room temperature for 2 minutes and analyzed by ^{19}F and ^{1}H NMR using $PhOCF_3$ (31.9 mg, 0.2 mmol) as an internal standard.

Figure S1. ^{19}F NMR spectrum of the above reaction mixture.

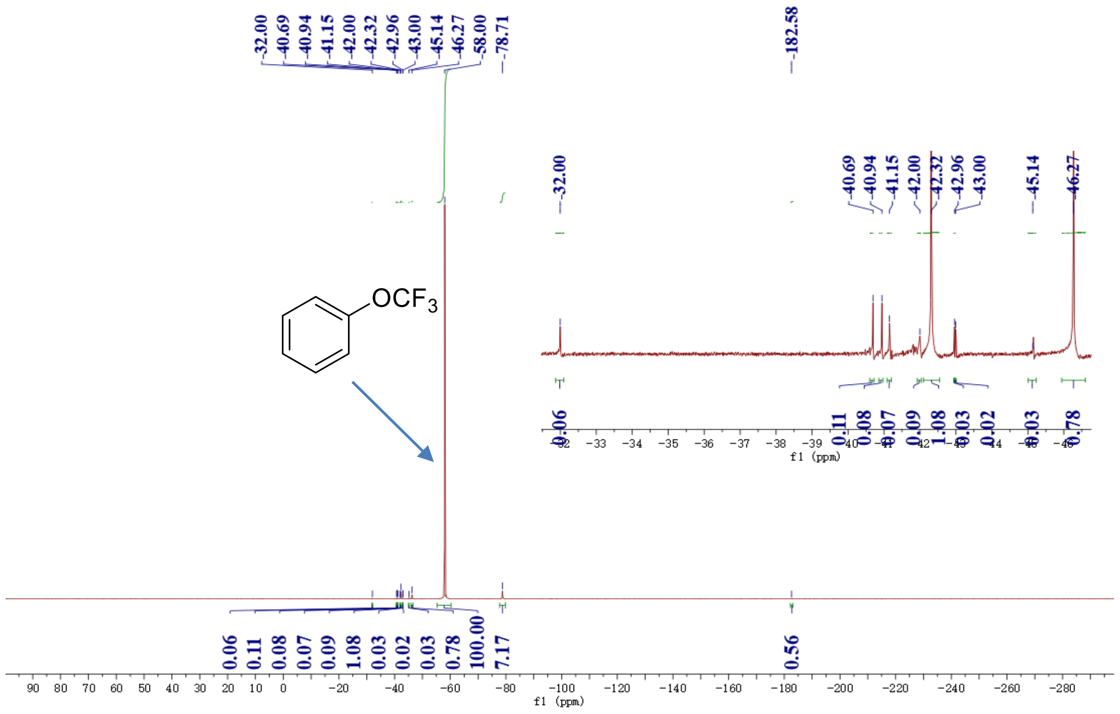

Figure S2. ^{1}H NMR spectrum of the above reaction mixture.

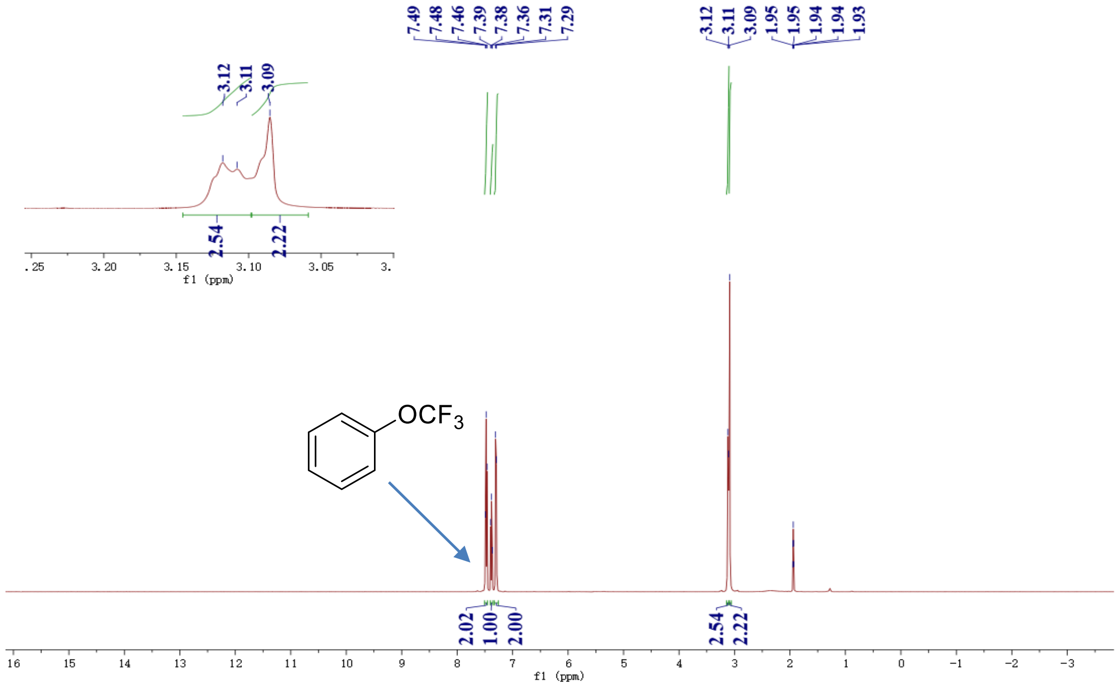

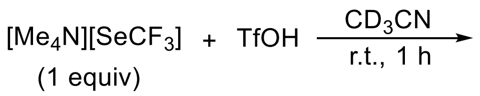

Experiment 2:

Under a nitrogen atmosphere, a sealed tube was charged with $[Me_4N][SeCF_3]$ (11.1 mg, 0.05 mmol) and a solution of TfOH (0.05 mmol) in CD_3CN (1 mL) with stirring.

The mixture was maintained at room temperature for 1 hour and analyzed by ^{19}F and ^{1}H NMR using $PhOCF_3$ (31.9 mg, 0.2 mmol) as an internal standard.

Figure S3. ^{19}F NMR spectrum of the above reaction mixture.

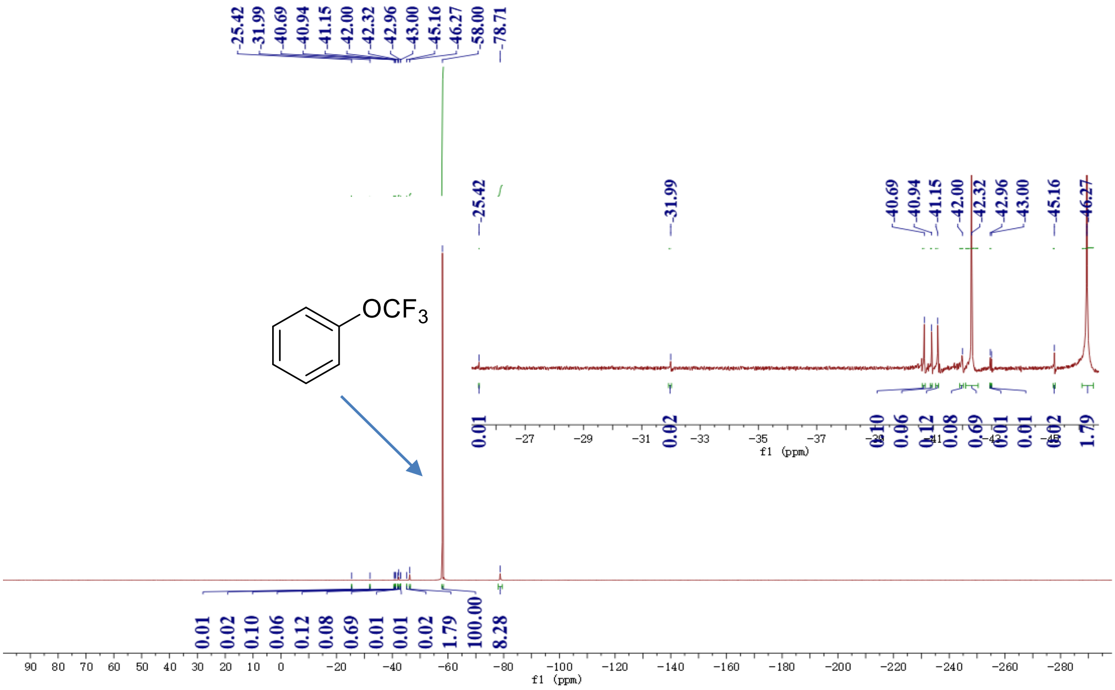

Figure S4. ^{1}H NMR spectrum of the above reaction mixture.

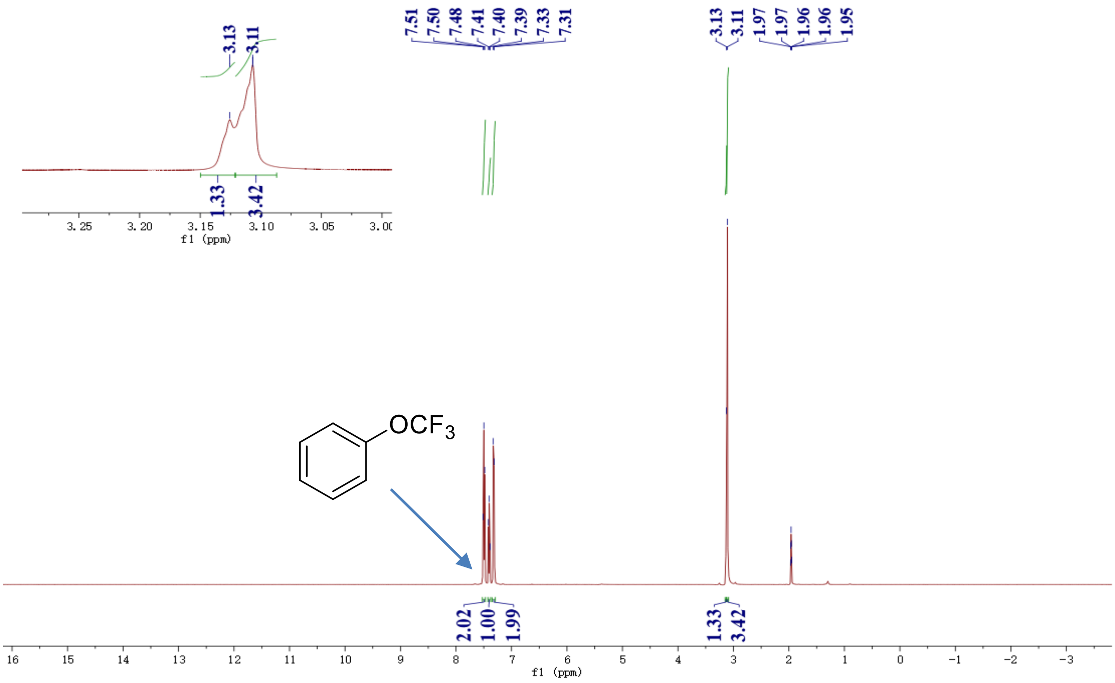

Figure S5. The combination of Figures 1 and 3

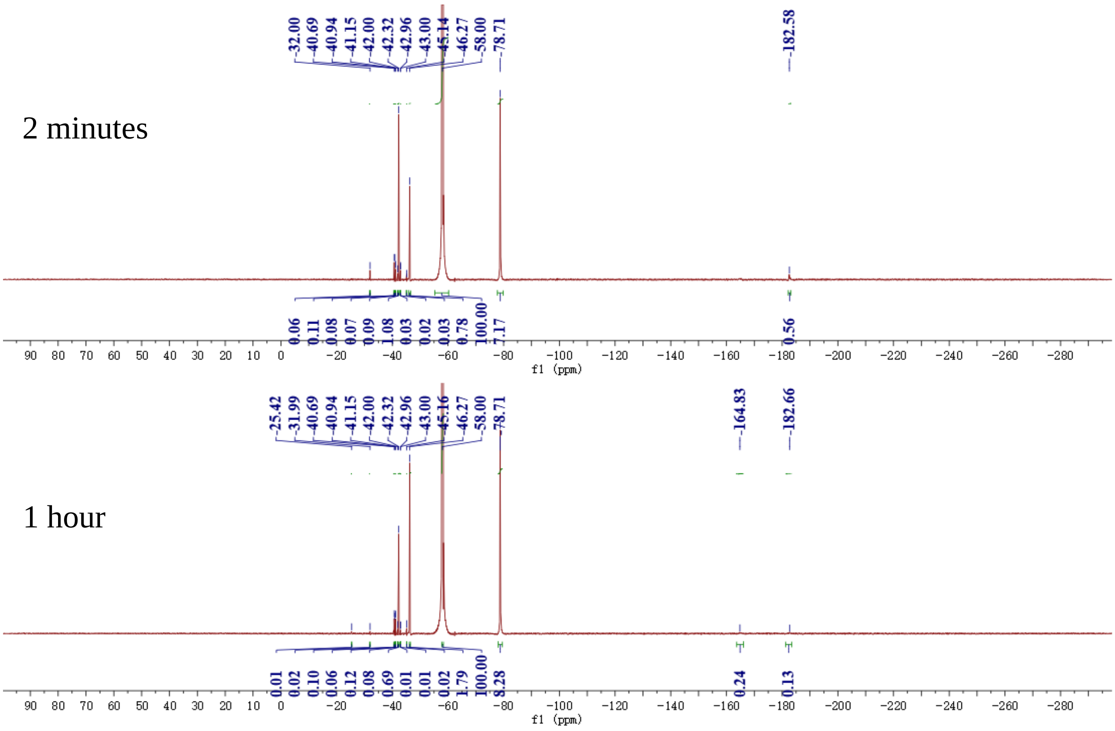

Figure S6. The combination of Figures 2 and 4

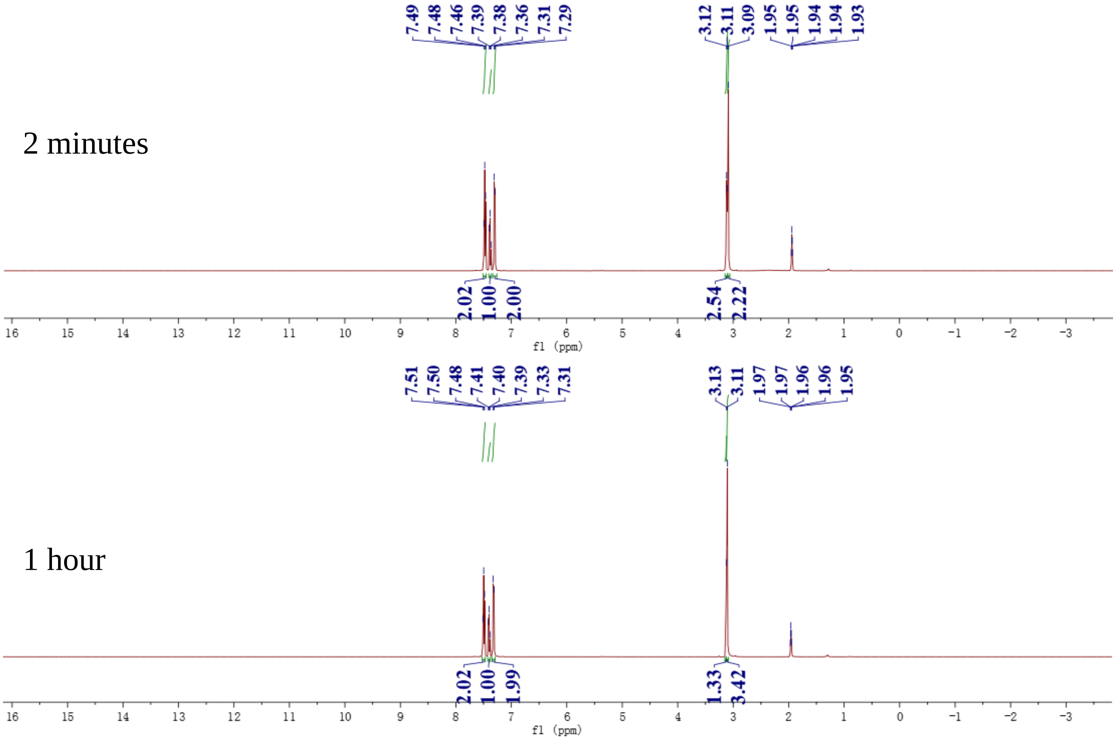

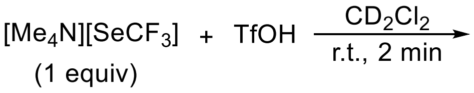

Experiment 3:

Under a nitrogen atmosphere, a sealed tube was charged with $[Me_4N][SeCF_3]$ (11.1

mg, 0.05 mmol) and a solution of TfOH (7.5 mg, 0.05 mmol) in CD_2Cl_2 (1 mL) with stirring. The mixture was maintained at room temperature for 2 minutes and analyzed by ^{19}F and ^{1}H NMR using $PhOCF_3$ (28.2 mg, 0.18 mmol) as an internal standard.

Figure S7. ^{19}F NMR spectrum of the above reaction mixture.

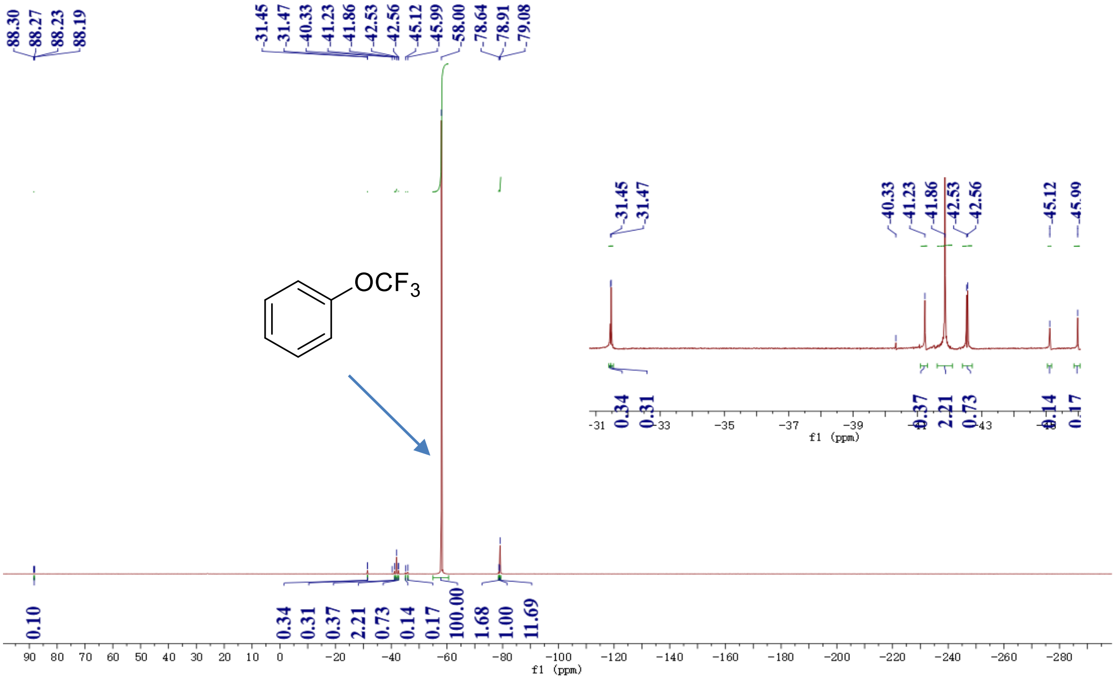

Figure S8. ^{1}H NMR spectrum of the above reaction mixture

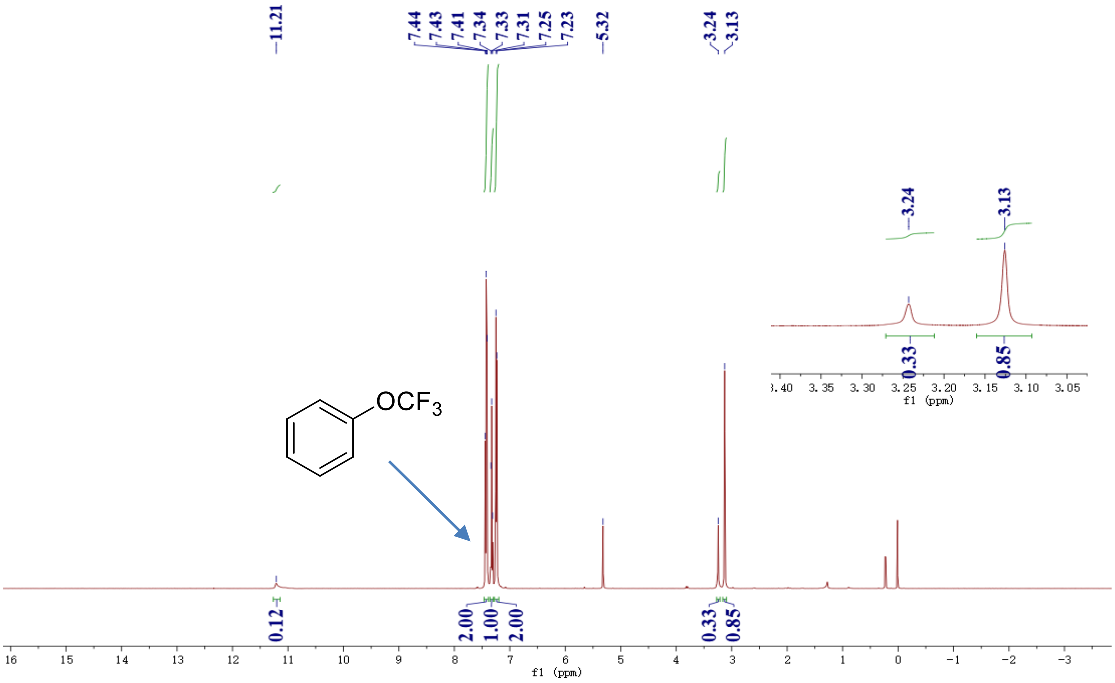

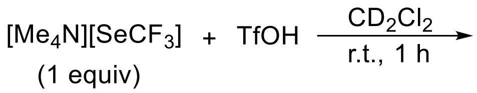

Experiment 4:

Under a nitrogen atmosphere, a sealed tube was charged with $[Me_4N][SeCF_3]$ (11.1 mg, 0.05 mmol) and a solution of TfOH (7.5 mg, 0.05 mmol) in CD_2Cl_2 (1 mL) with stirring. The mixture was maintained at room temperature for 1 hour and analyzed by ^{19}F and ^{1}H NMR using $PhOCF_3$ (28.2 mg, 0.18 mmol) as an internal standard.

Figure S9. ^{19}F NMR spectrum of the above reaction mixture.

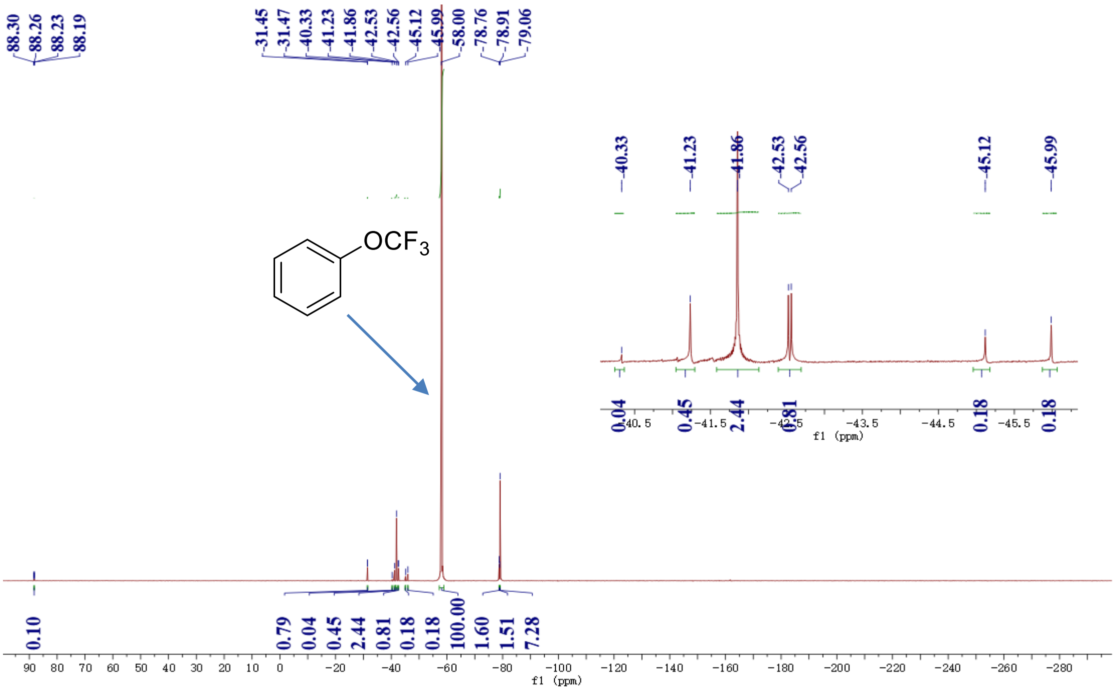

Figure S10. ^{1}H NMR spectrum of the above reaction mixture.

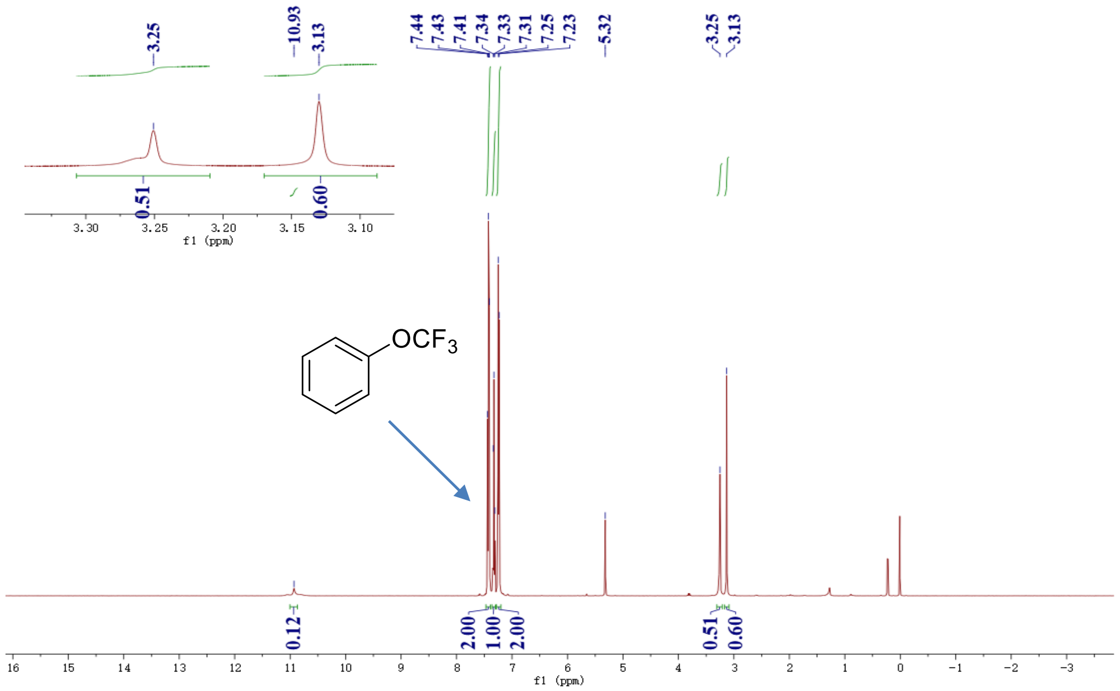

Figure S11. The combination of Figures 7 and 9

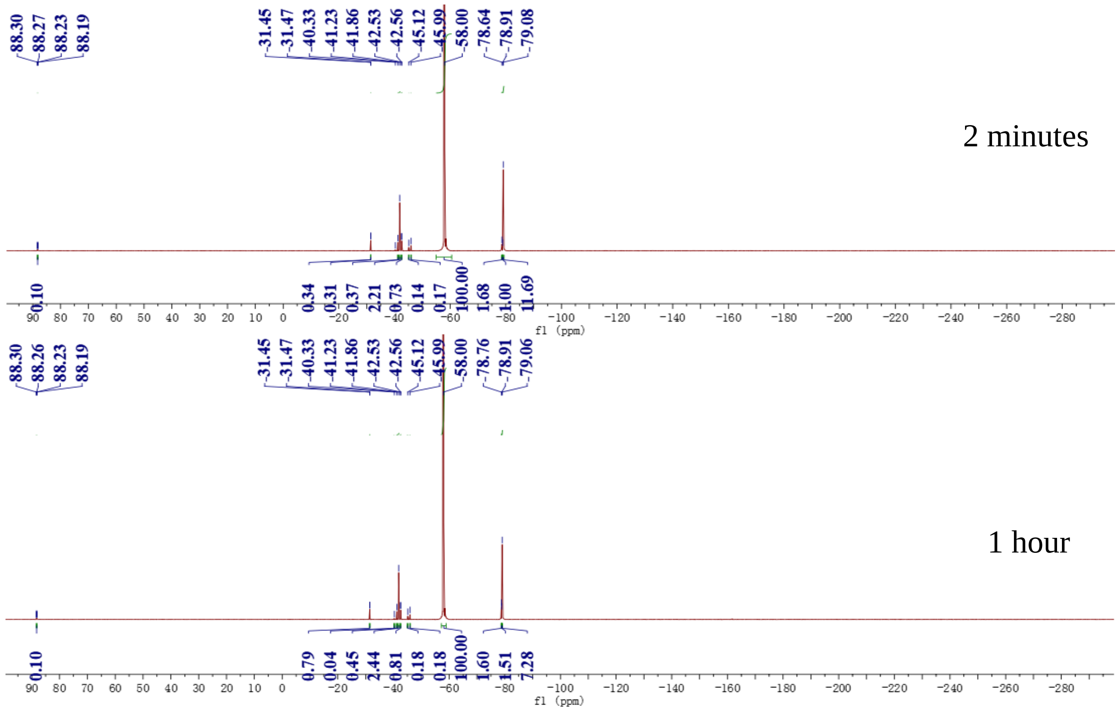

Figure S12. The combination of Figures 8 and 10

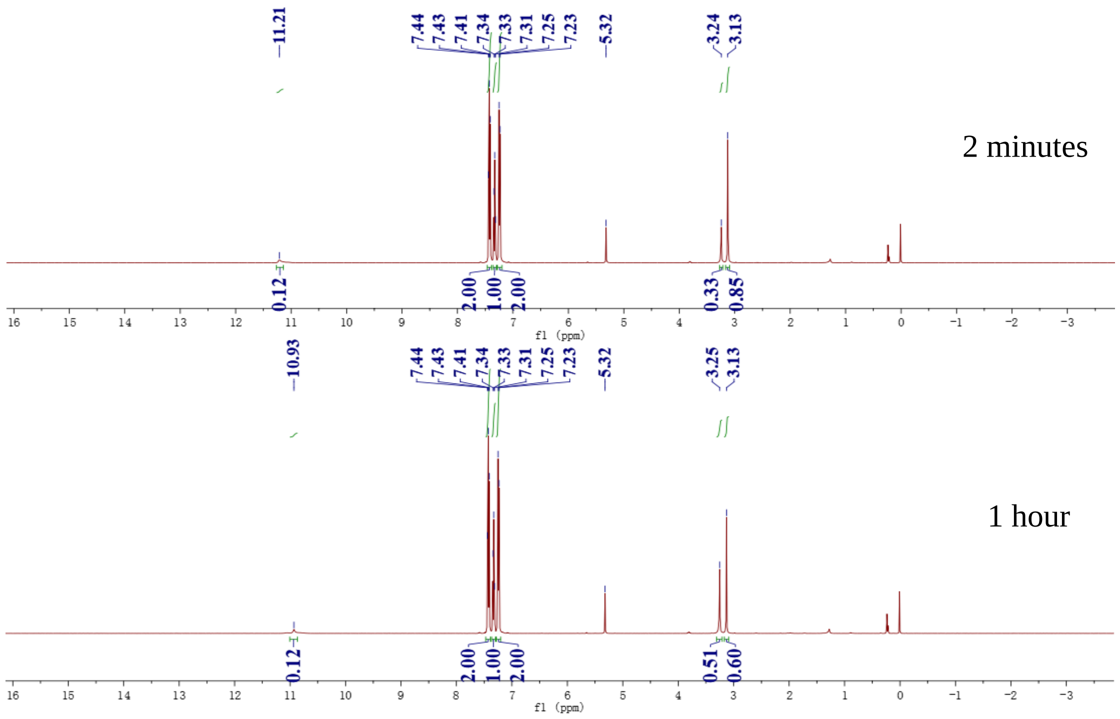

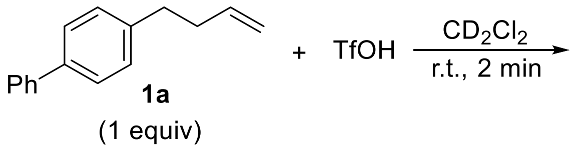

Experiment 5:

Under a nitrogen atmosphere, a sealed tube was charged with **1a** (10.4 mg, 0.05 mmol) and a solution of TfOH (7.5 mg, 0.05 mmol) in CD_2Cl_2 (1 mL) with stirring. The mixture was maintained at room temperature for 2 minutes and analyzed by ^{19}F and ^{1}H NMR using $PhOCF_3$ (28.0 mg, 0.18 mmol) as an internal standard.

Figure S13. ^{19}F NMR spectrum of the above reaction mixture.

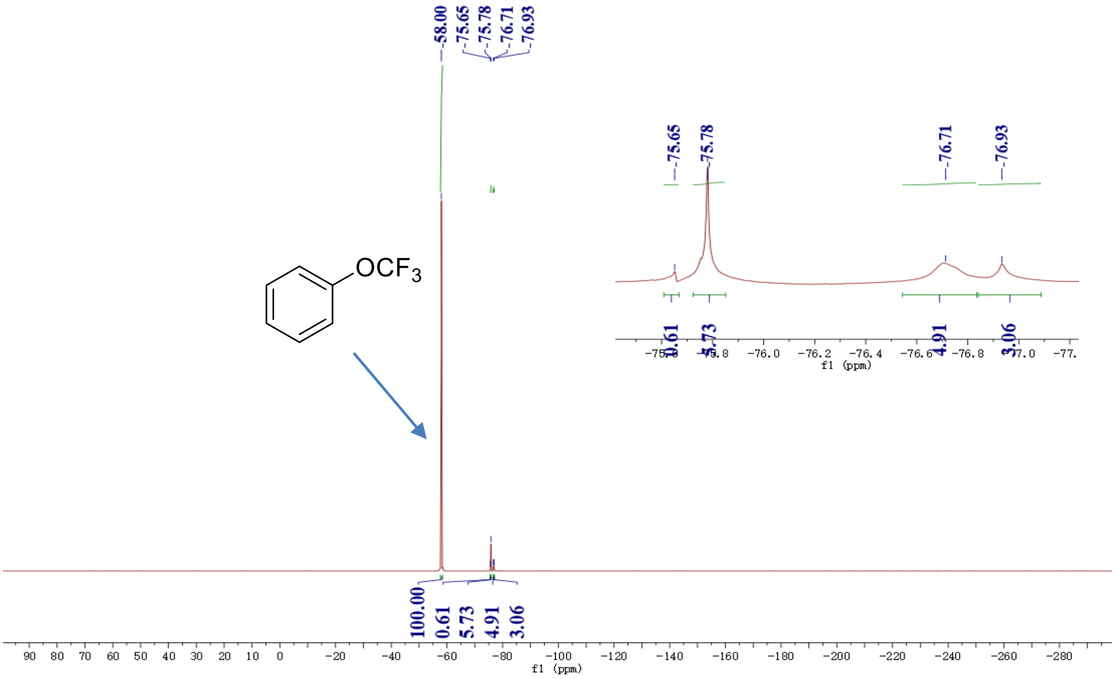

Figure S14. ¹H NMR spectrum of the above reaction mixture.

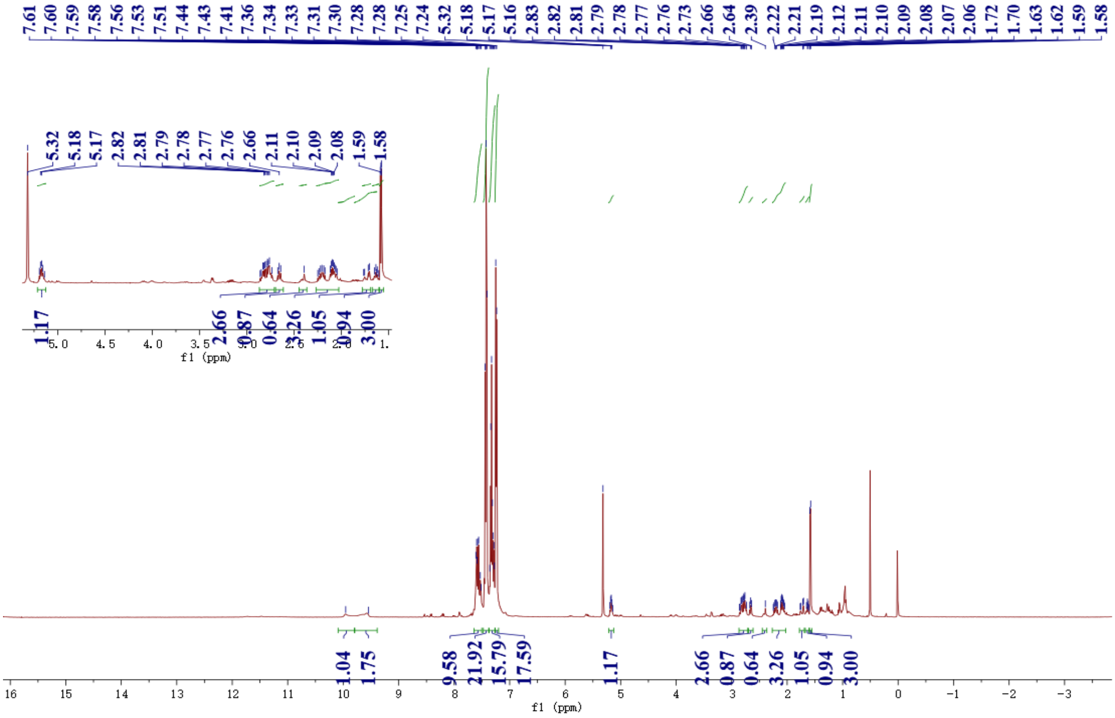

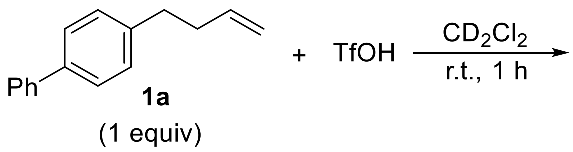

Experiment 6:

Under a nitrogen atmosphere, a sealed tube was charged with **1a** (10.4 mg, 0.05 mmol) and a solution of TfOH (7.5 mg, 0.05 mmol) in CD_2Cl_2 (1 mL) with stirring. The

mixture was maintained at room temperature for 1 hour and analyzed by ^{19}F and ^{1}H NMR using $PhOCF_3$ (28.0 mg, 0.18 mmol) as an internal standard.

Figure S15. ^{19}F NMR spectrum of the above reaction mixture.

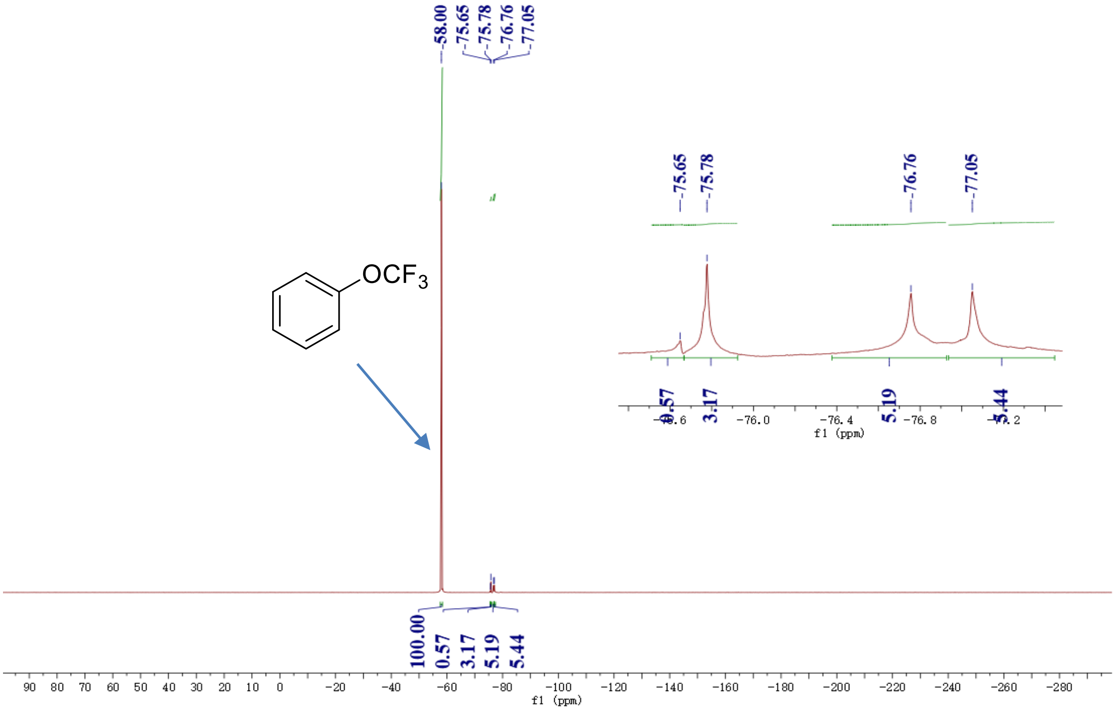

Figure S16. ^{1}H NMR spectrum of the above reaction mixture.

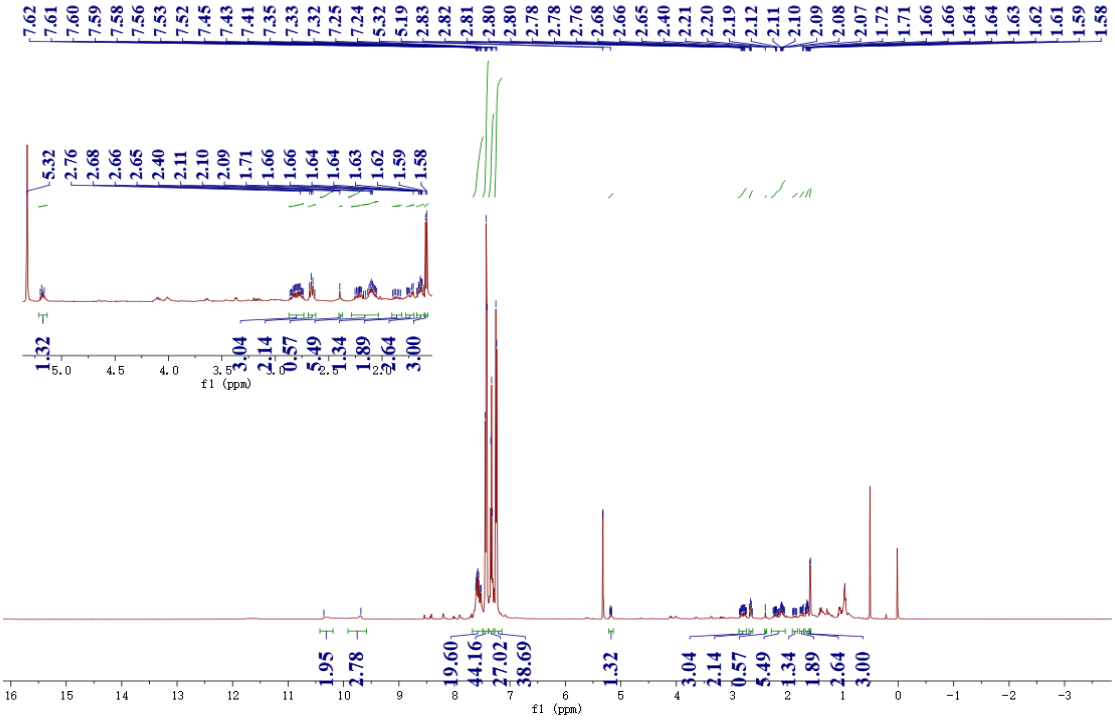

Figure S17. The combination of Figures 13 and 15

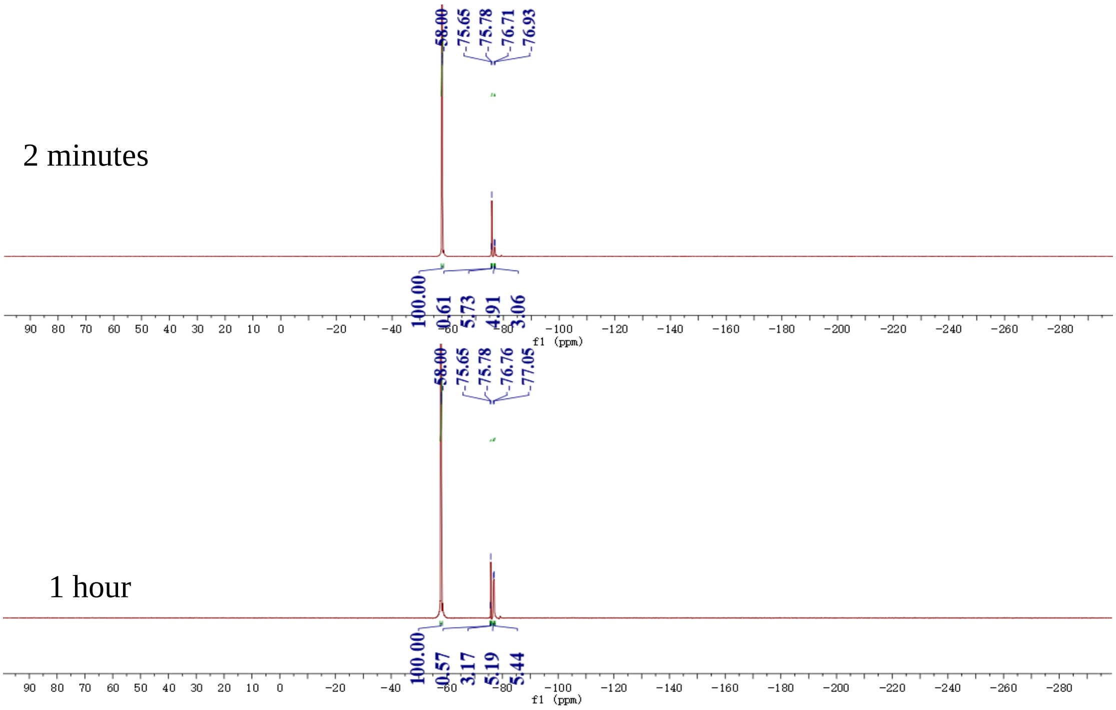

Figure S18. The combination of Figures 14 and 16

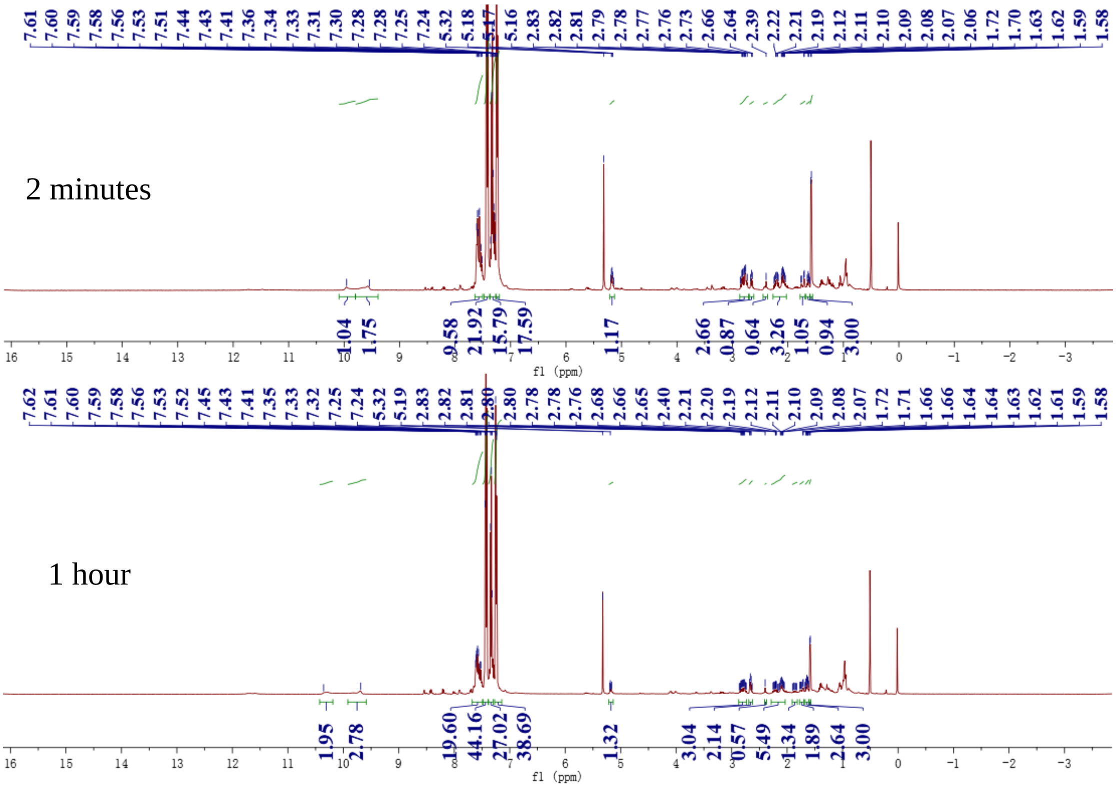

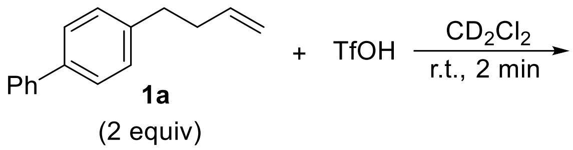

Experiment 7:

Under a nitrogen atmosphere, a sealed tube was charged with **1a** (20.8 mg, 0.10 mmol) and a solution of TfOH (7.5 mg, 0.05 mmol) in CD_2Cl_2 (1 mL) with stirring. The mixture was maintained at room temperature for 2 minutes and analyzed by ^{19}F and ^{1}H NMR using $PhOCF_3$ (26.5 mg, 0.17 mmol) as an internal standard.

Figure S19. ^{19}F NMR spectrum of the above reaction mixture.

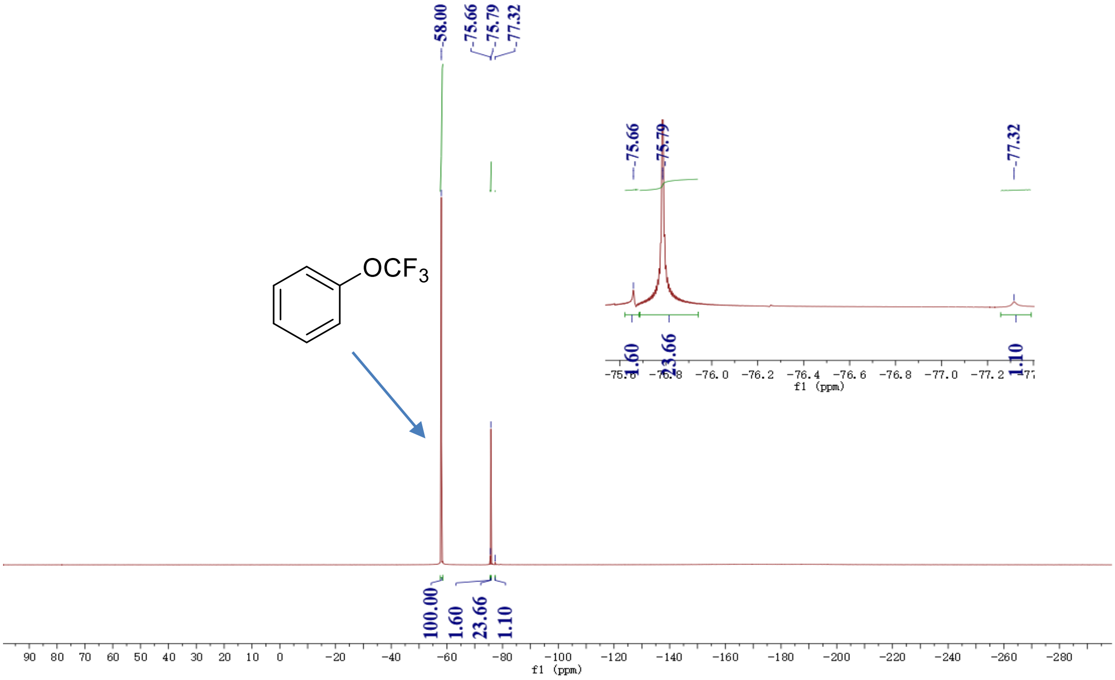

Figure S20. ^{1}H NMR spectrum of the above reaction mixture.

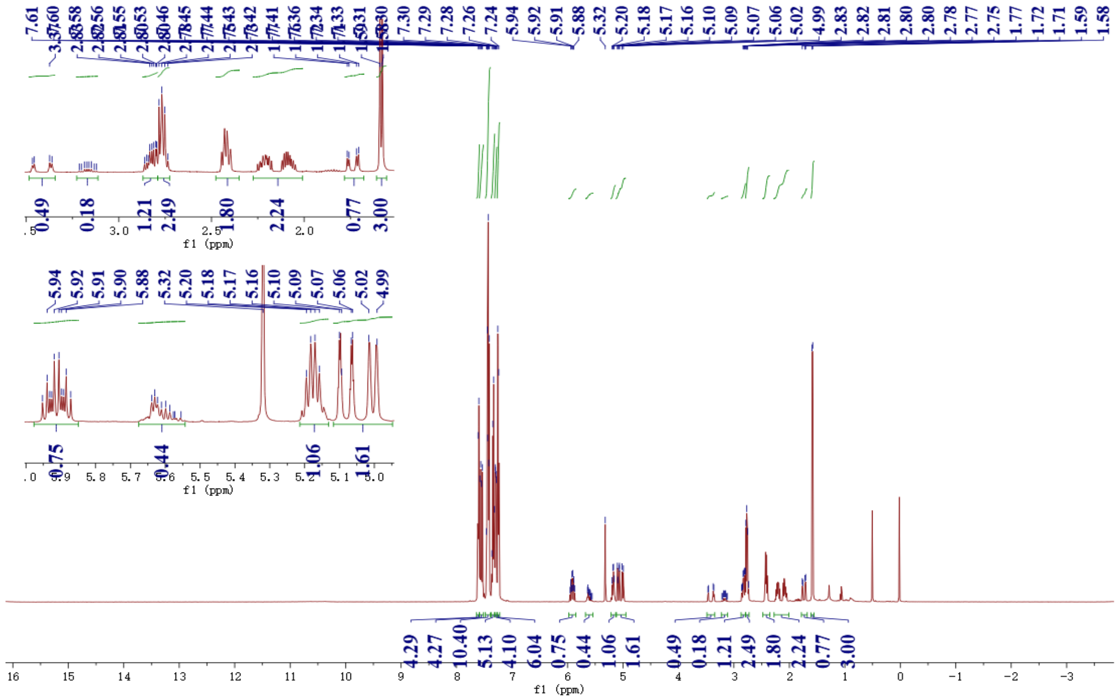

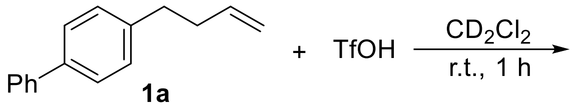

Experiment 8:

Under a nitrogen atmosphere, a sealed tube was charged with **1a** (20.8 mg, 0.10 mmol) and a solution of TfOH (7.5 mg, 0.05 mmol) in CD_2Cl_2 (1 mL) with stirring. The mixture was maintained at room temperature for 1 hour and analyzed by ^{19}F and ^{1}H NMR using $PhOCF_3$ (26.5 mg, 0.17 mmol) as an internal standard.

Figure S21. ^{19}F NMR spectrum of the above reaction mixture.

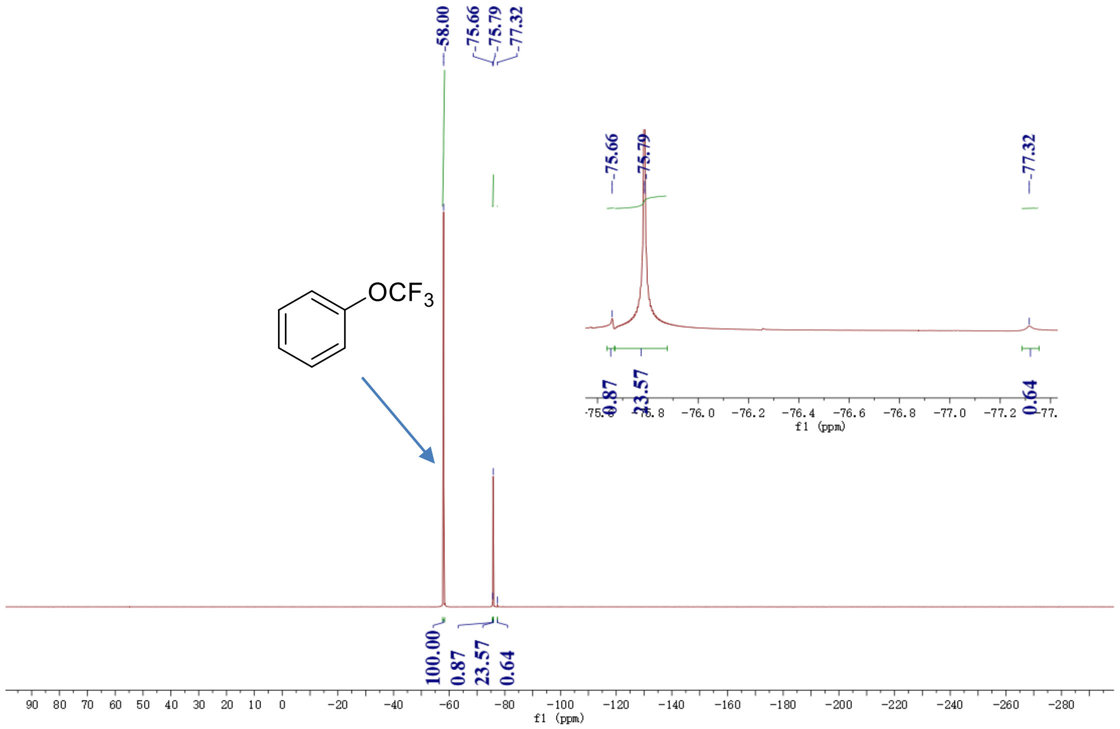

Figure S22. ^{1}H NMR spectrum of the above reaction mixture.

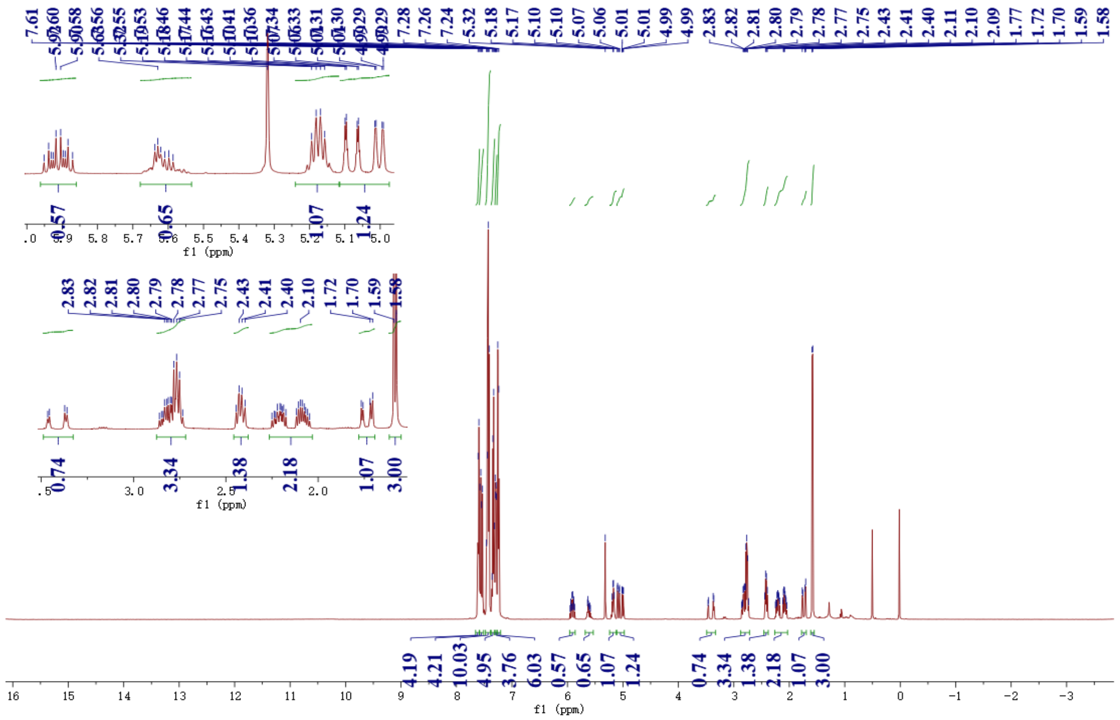

Figure S23. The combination of Figures 19 and 21

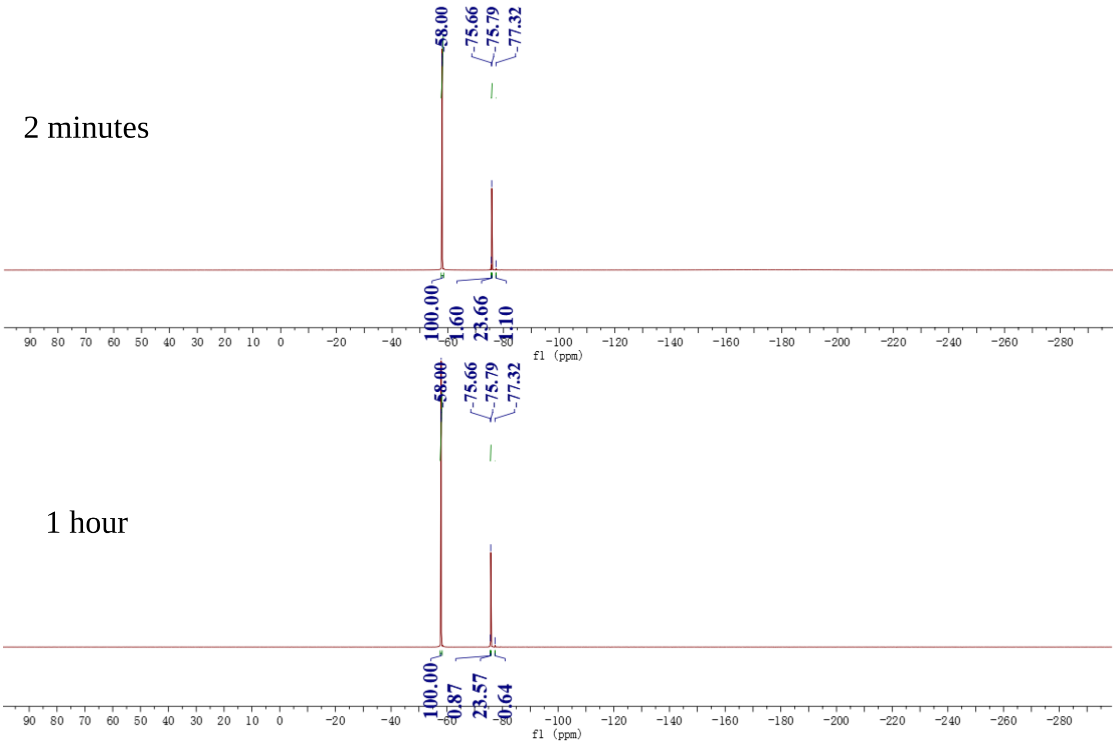

Figure S24. The combination of Figures 20 and 22

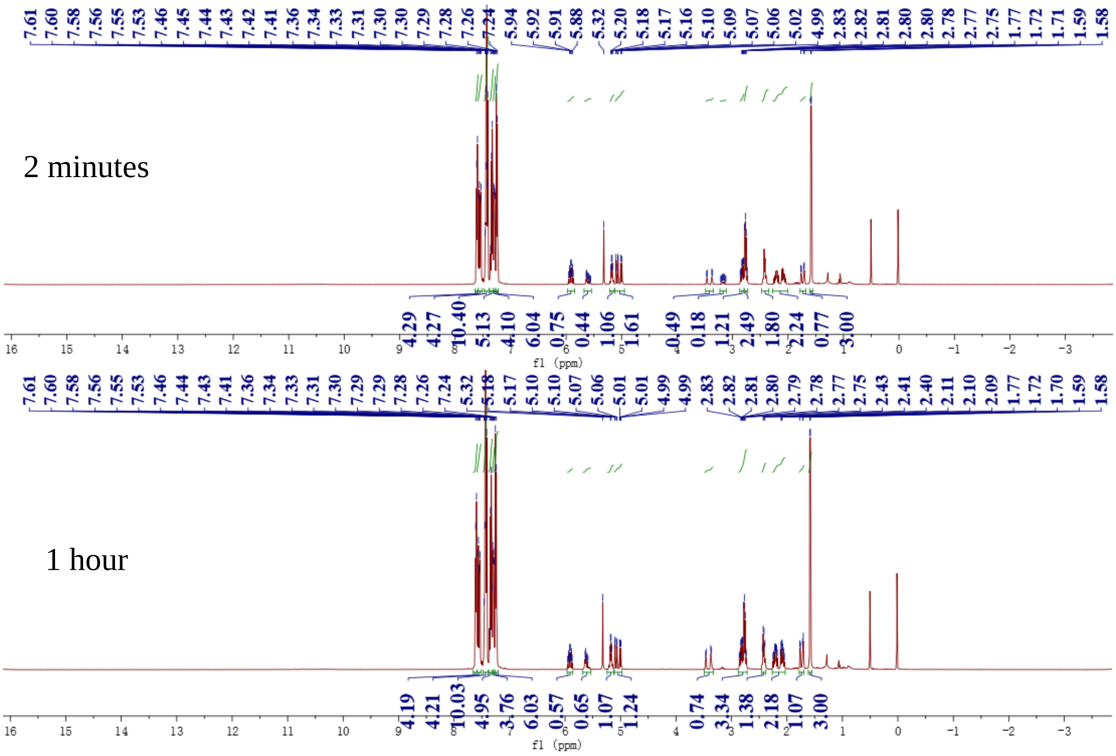

Reference:

[1] a) Roesky, H. W. *Efficient Preparations of Fluorine Compounds.* John Wiley & Sons, Inc. **2013**; b) Qi, X.-X.; Chen, P.-H.; Liu, G.-S. *Angew. Chem. Int. Ed.* **2017**, *56*,

9517-9521.

[2] Liu, R.; Lu, Z.-H.; Hu, X.-H.; Li, J.-L.; Yang, X.-J. *Org. Lett.* **2015**, *17*, 1489-1492.

[3] Brown, L. J.; Brown, R. C. D.; Raja, R. *RSC Adv*. **2013**, *3*, 843-850.

[4] Meng, Q.-Y.; Schirmer, T. E.; Katou, K.; König, B. *Angew. Chem. Int. Ed*. **2019**, *58*, 5723-5728.

[5] Armarego, W. L. F.; Chai, C. L. L. *Purification of Laboratory Chemicals*, 5th ed.; Butterworth Heinemann: Oxford, **2003**

[6] Dong, T.; He, J.; Li, Z.-H.; Zhang, C.-P. *ACS Sustainable Chem. Eng.* **2018**, *6*, 1327-1335.

7. NMR spectra of the products.

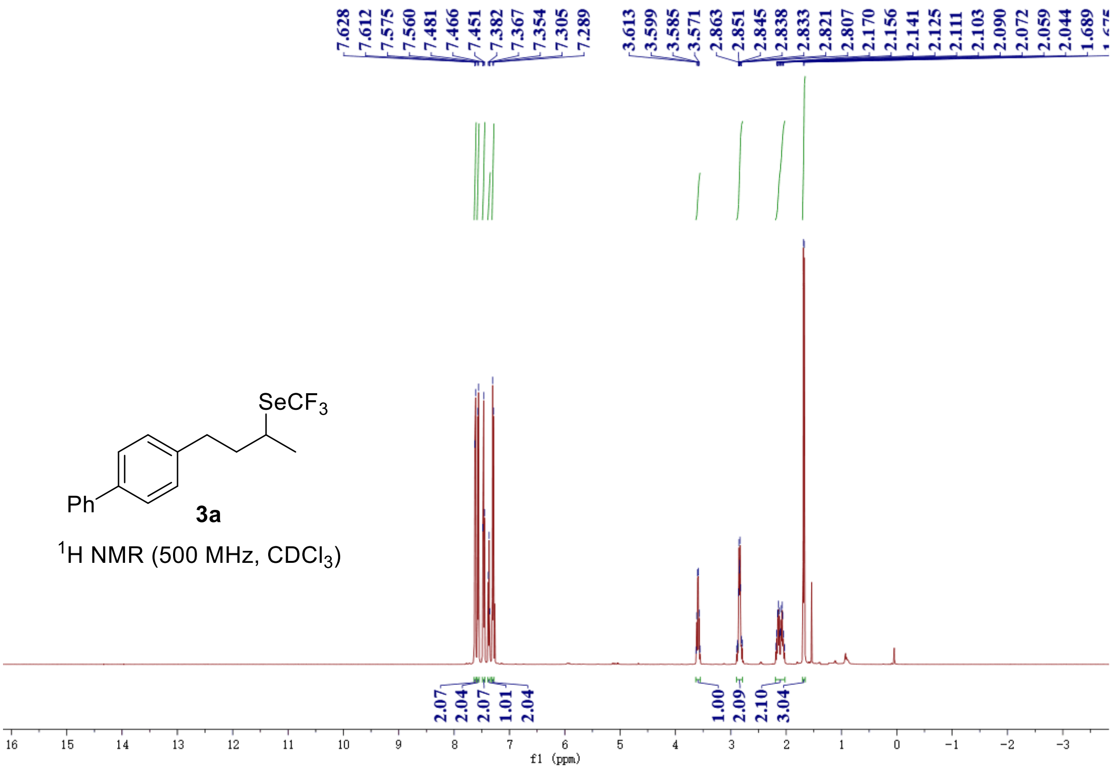

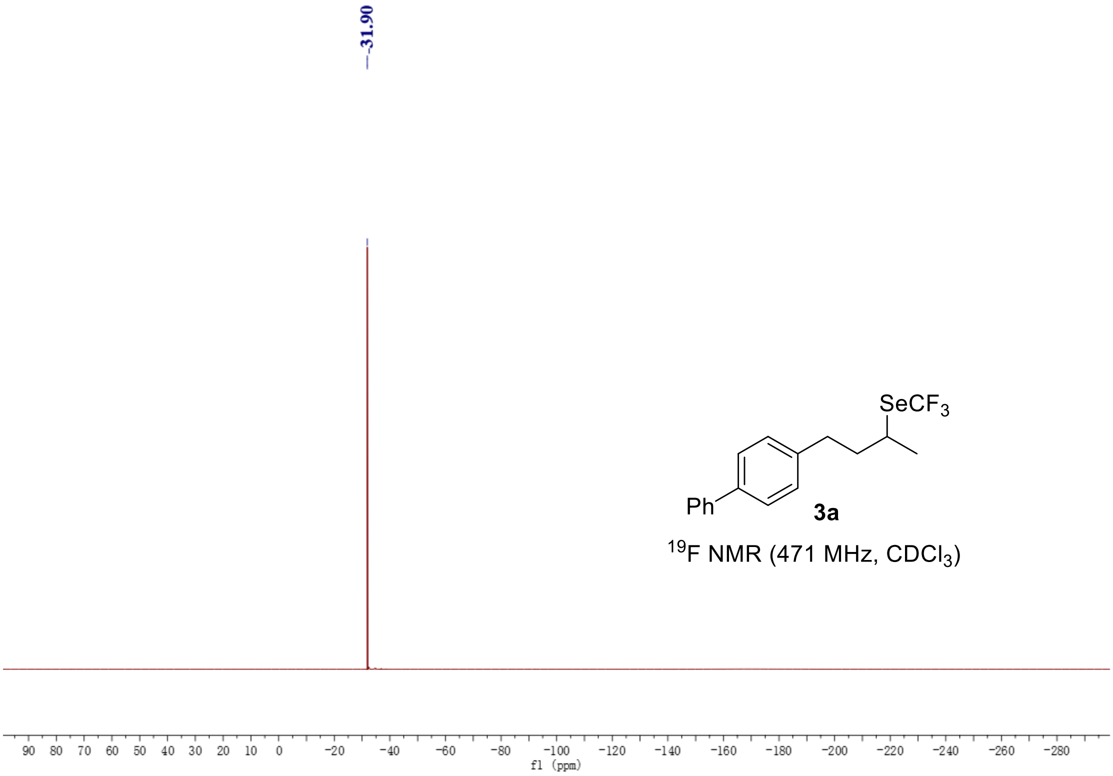

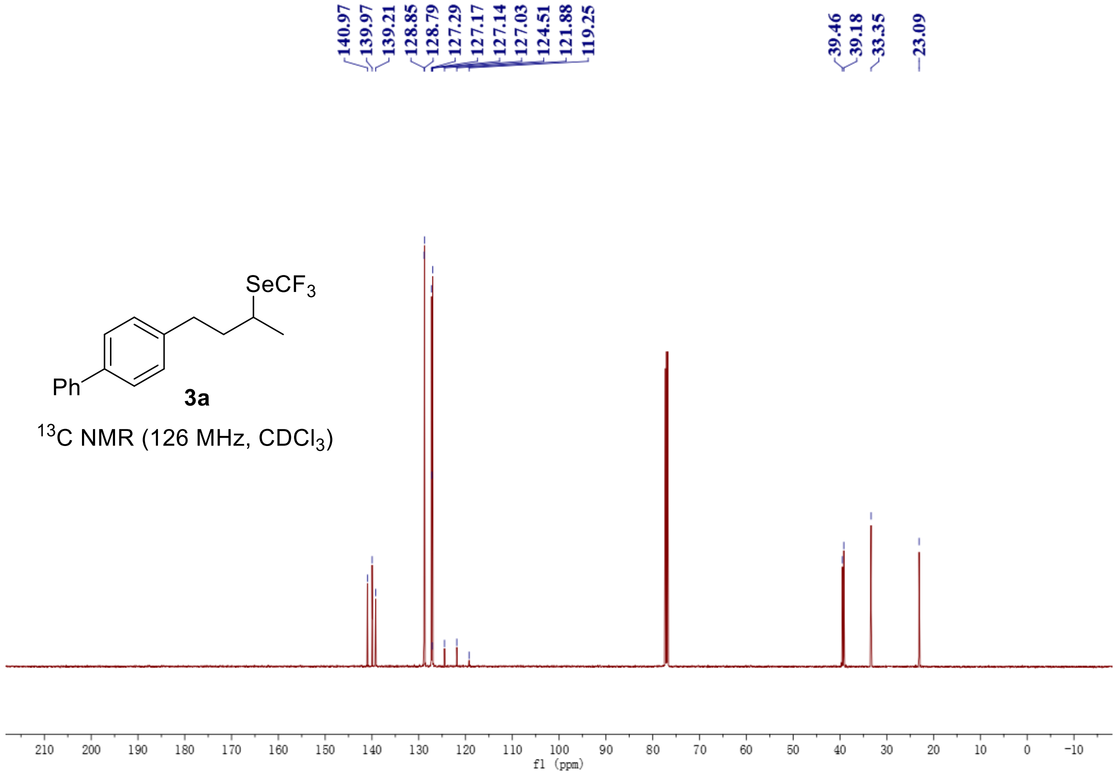
140.97
139.97
139.21
128.85
128.79
127.29
127.17
127.14
127.03
124.51
121.88
119.25
39.46
39.18
33.35
23.09
SeCF3
Ph
3a
13C NMR (126 MHz, CDCl3)
f1 (ppm)

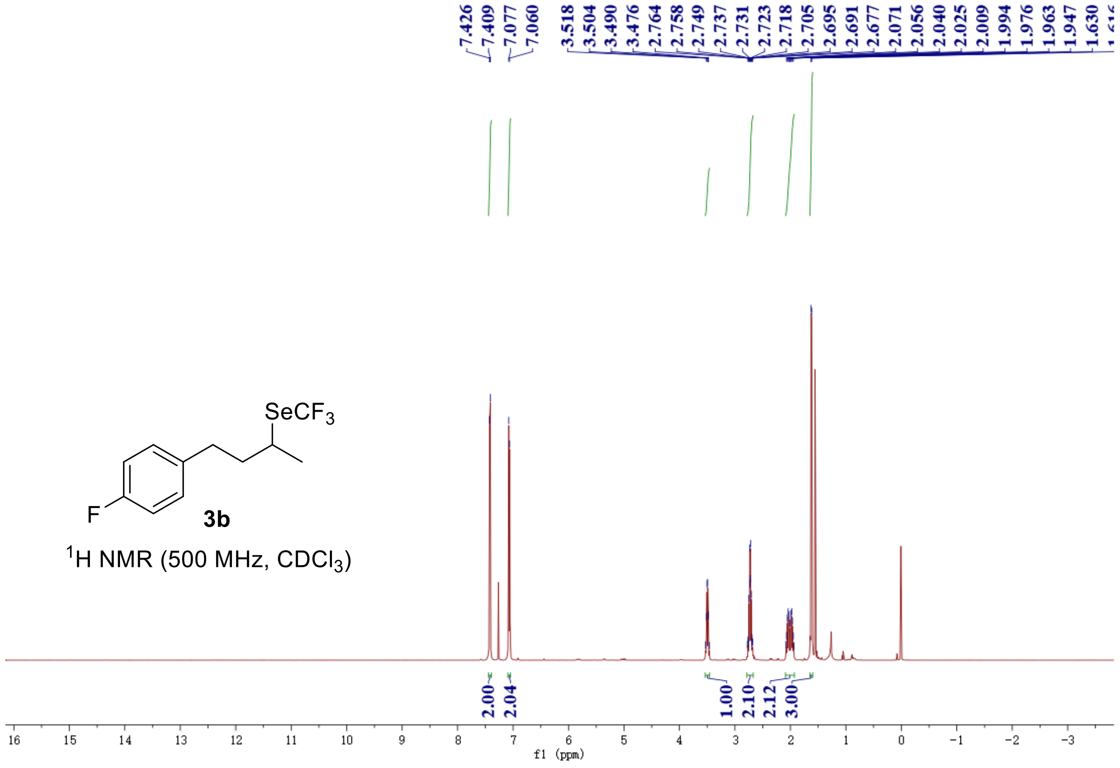
7.426
7.409
7.077
7.060
3.518
3.504
3.490
3.476
2.764
2.758
2.749
2.737
2.731
2.723
2.718
2.705
2.695
2.691
2.677
2.071
2.056
2.040
2.025
2.009
1.994
1.976
1.963
1.947
1.630
SeCF3
F
3b
1H NMR (500 MHz, CDCl3)
2.00
2.04
1.00
2.10
2.12
3.00
f1 (ppm)

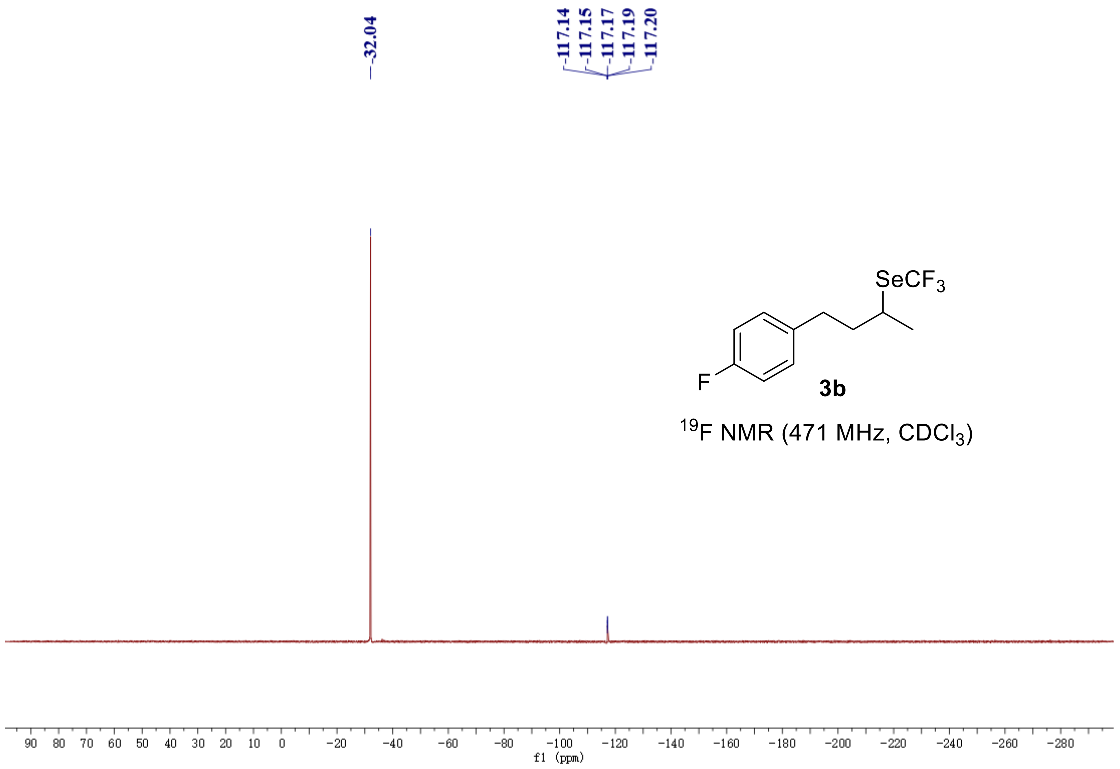
-32.04
-117.14
-117.15
-117.17
-117.19
-117.20
SeCF3
F
3b
19F NMR (471 MHz, CDCl3)
f1 (ppm)

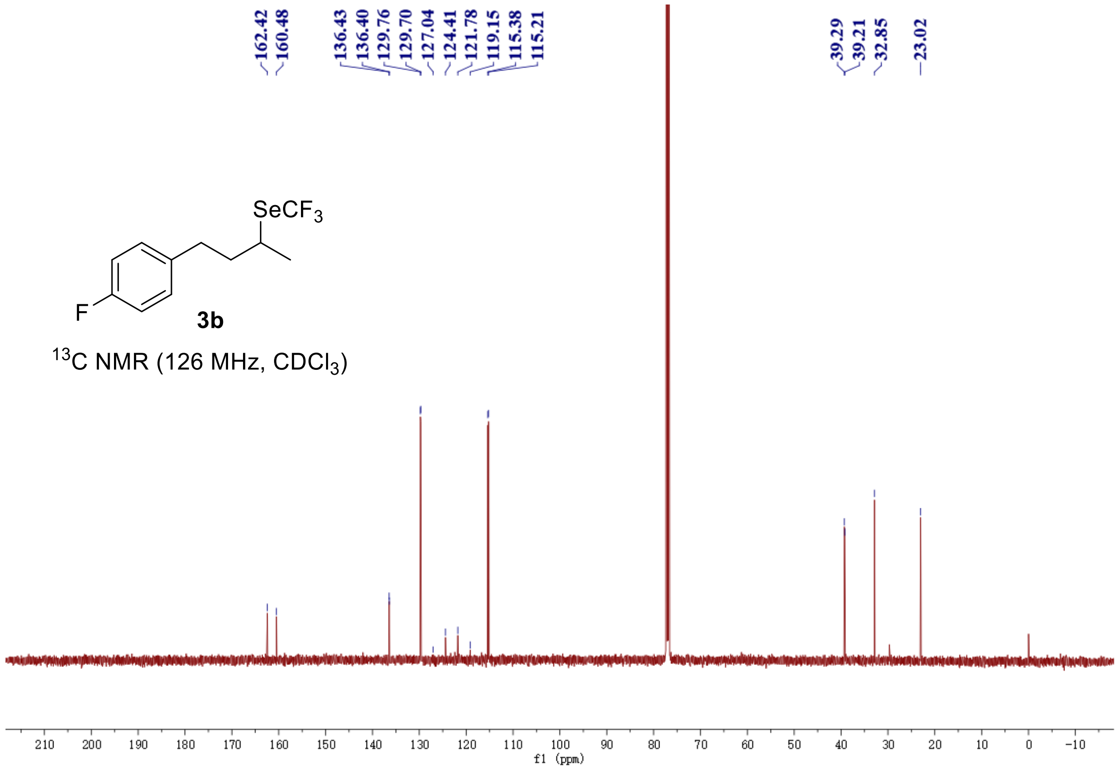
162.42
160.48
136.43
136.40
129.76
129.70
127.04
124.41
121.78
119.15
115.38
115.21
39.29
39.21
32.85
23.02
SeCF3
F
3b
13C NMR (126 MHz, CDCl3)
f1 (ppm)

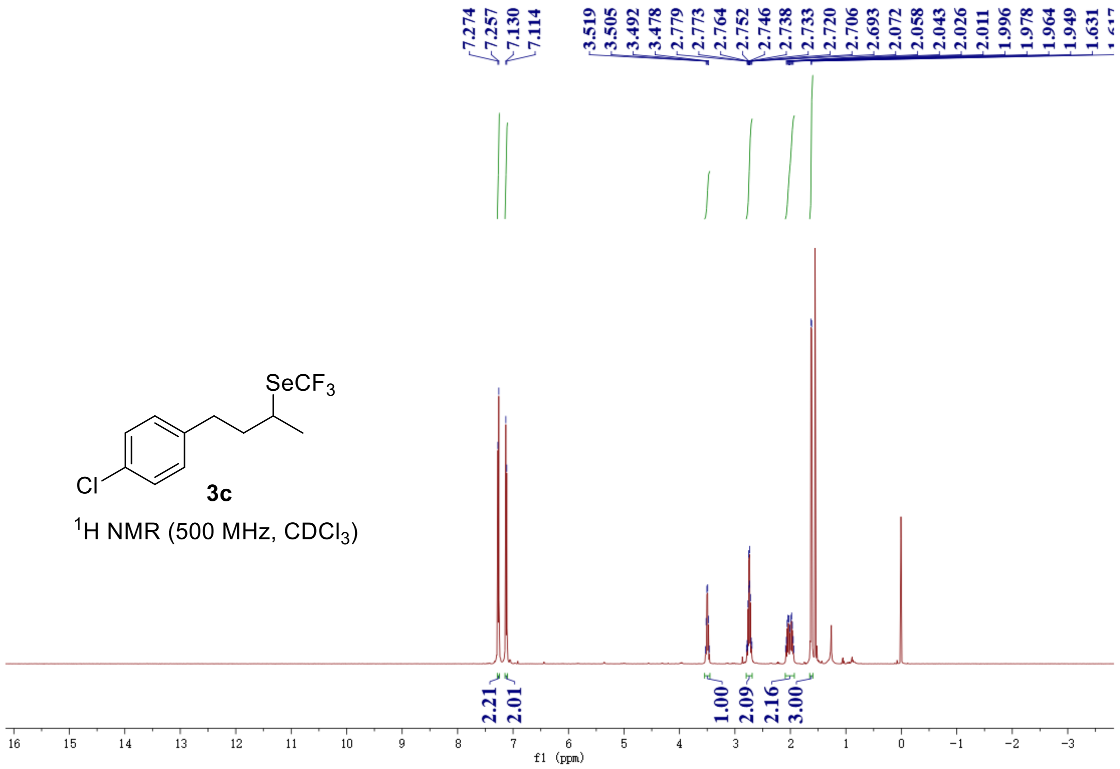

7.274
7.257
7.130
7.114
3.519
3.505
3.492
3.478
2.779
2.773
2.764
2.752
2.746
2.738
2.733
2.720
2.706
2.693
2.072
2.058
2.043
2.026
2.011
1.996
1.978
1.964
1.949
1.631
SeCF3
Cl
3c
1H NMR (500 MHz, CDCl3)
2.21
2.01
1.00
2.09
2.16
3.00
f1 (ppm)

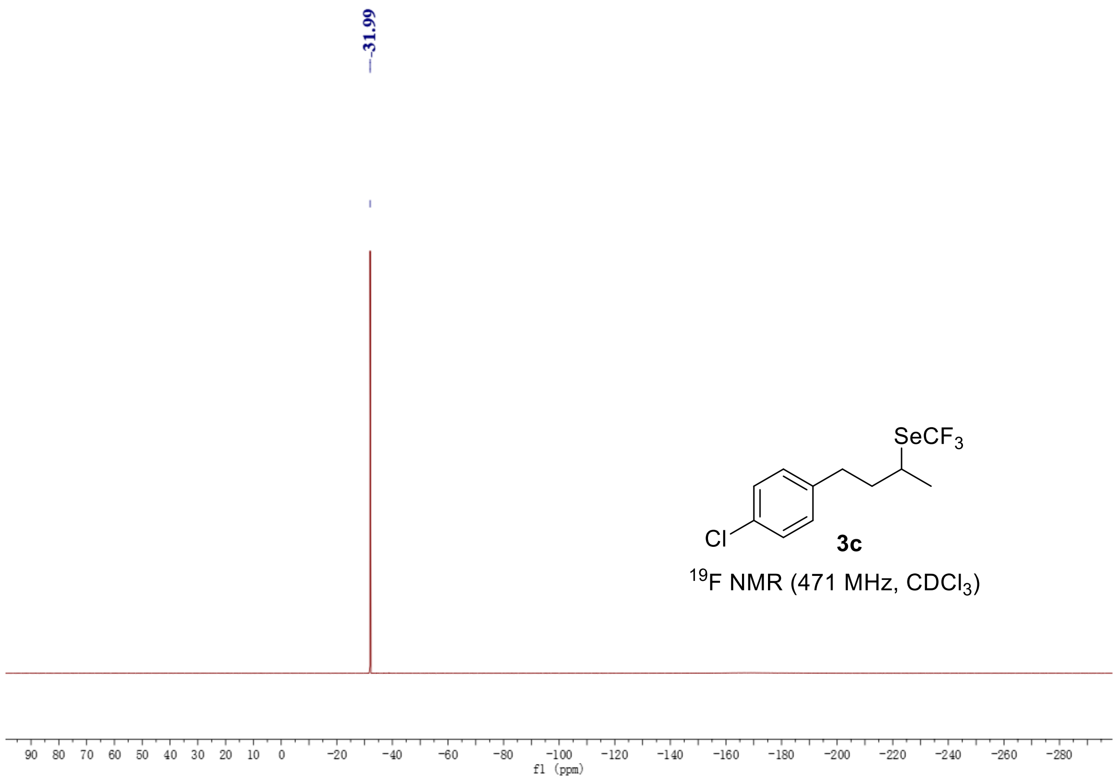

-31.99
SeCF3
Cl
3c
19F NMR (471 MHz, CDCl3)
f1 (ppm)

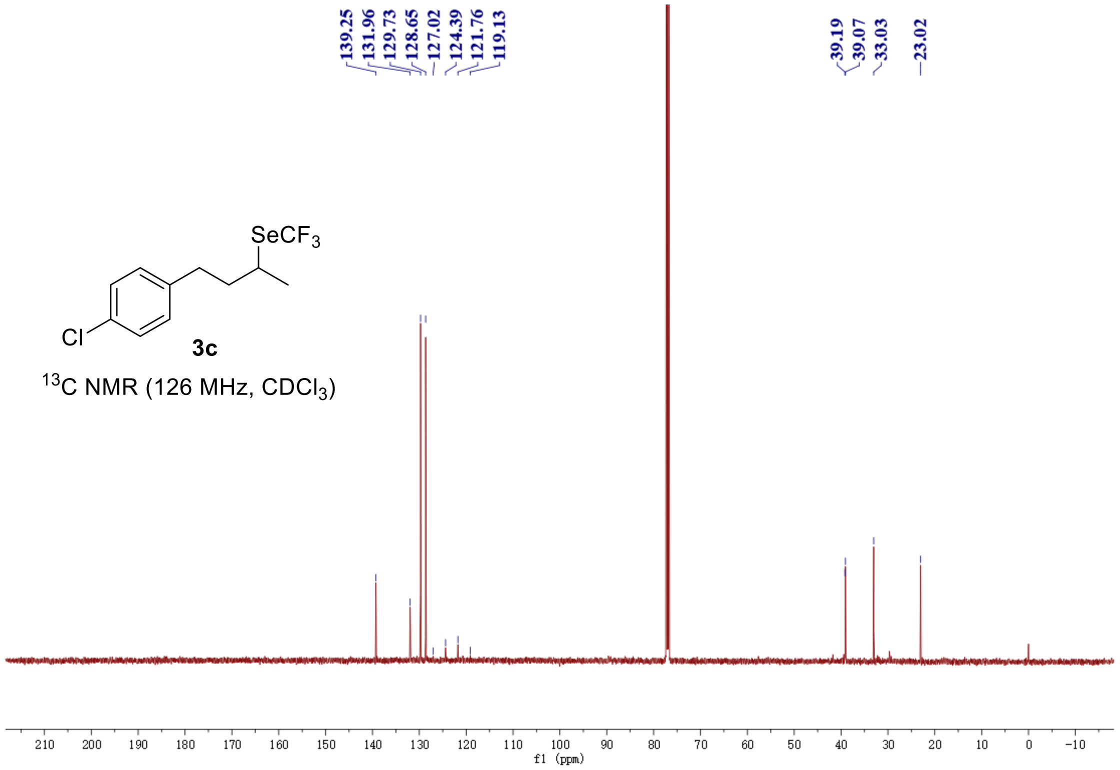
139.25
131.96
129.73
128.65
127.02
124.39
121.76
119.13
39.19
39.07
33.03
23.02
SeCF3
Cl
3c
13C NMR (126 MHz, CDCl3)
f1 (ppm)

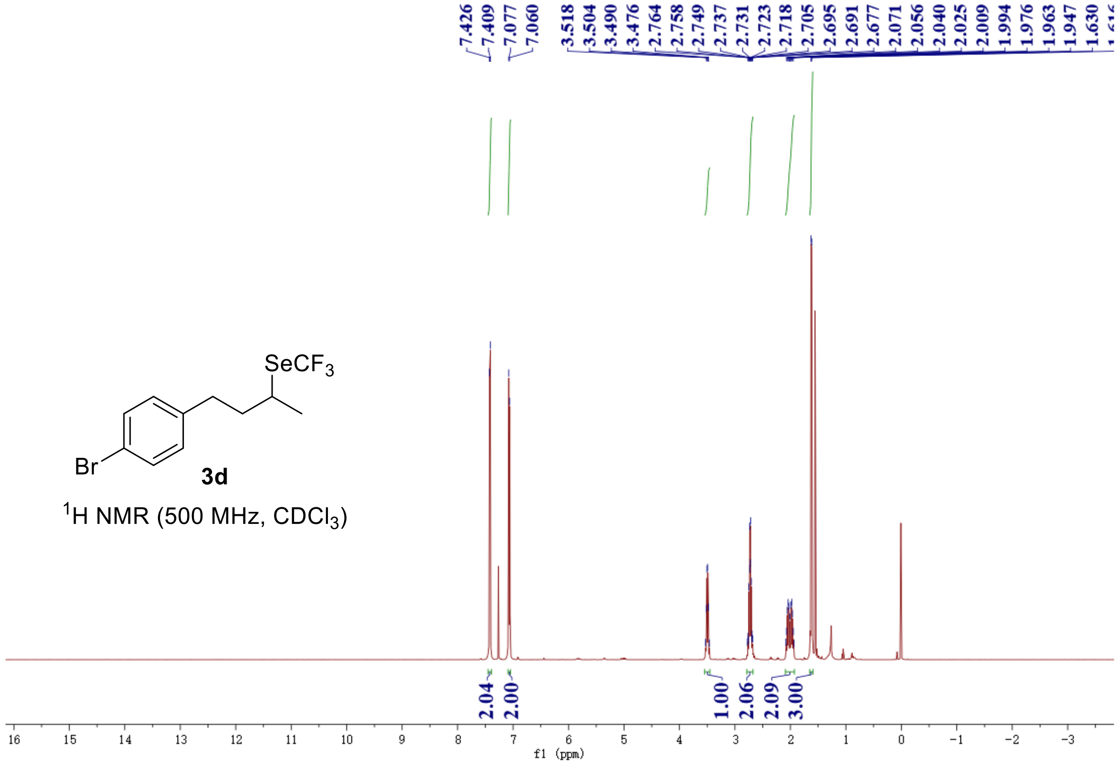
7.426
7.409
7.077
7.060
3.518
3.504
3.490
3.476
2.764
2.758
2.749
2.737
2.731
2.723
2.718
2.705
2.695
2.691
2.677
2.071
2.056
2.040
2.025
2.009
1.994
1.976
1.963
1.947
1.630
SeCF3
Br
3d
1H NMR (500 MHz, CDCl3)
2.04
2.00
1.00
2.06
2.09
3.00
f1 (ppm)

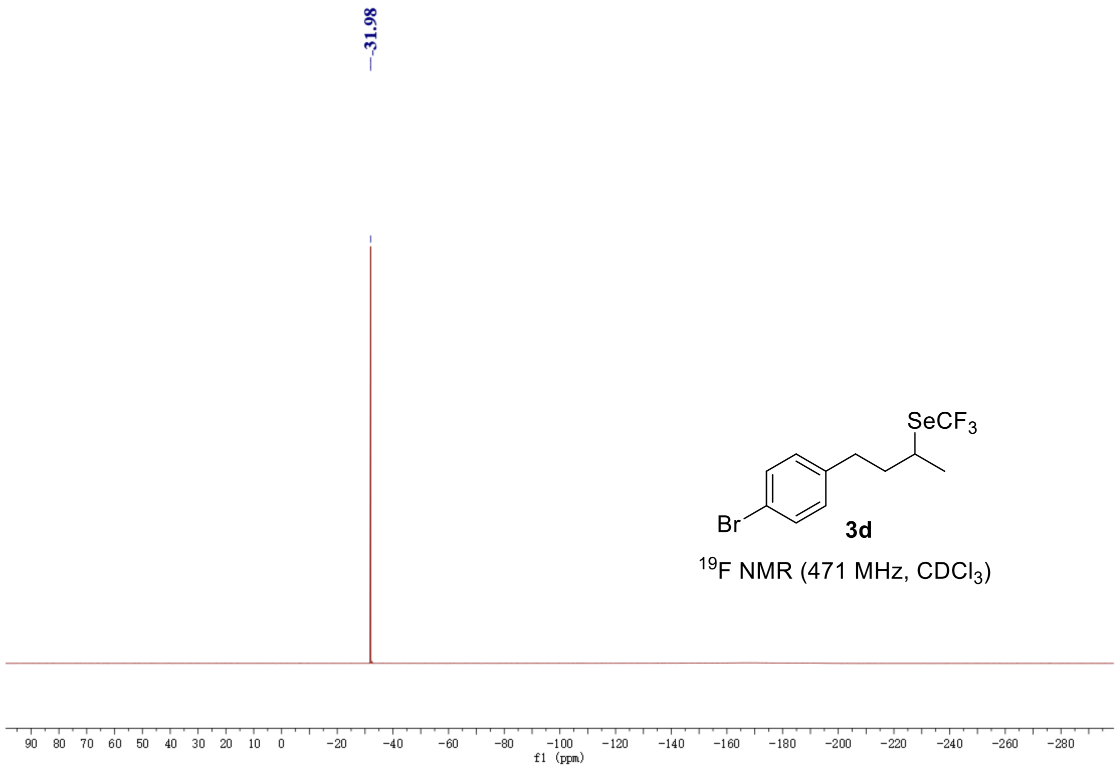

-31.98
SeCF3
Br
3d
19F NMR (471 MHz, CDCl3)
f1 (ppm)

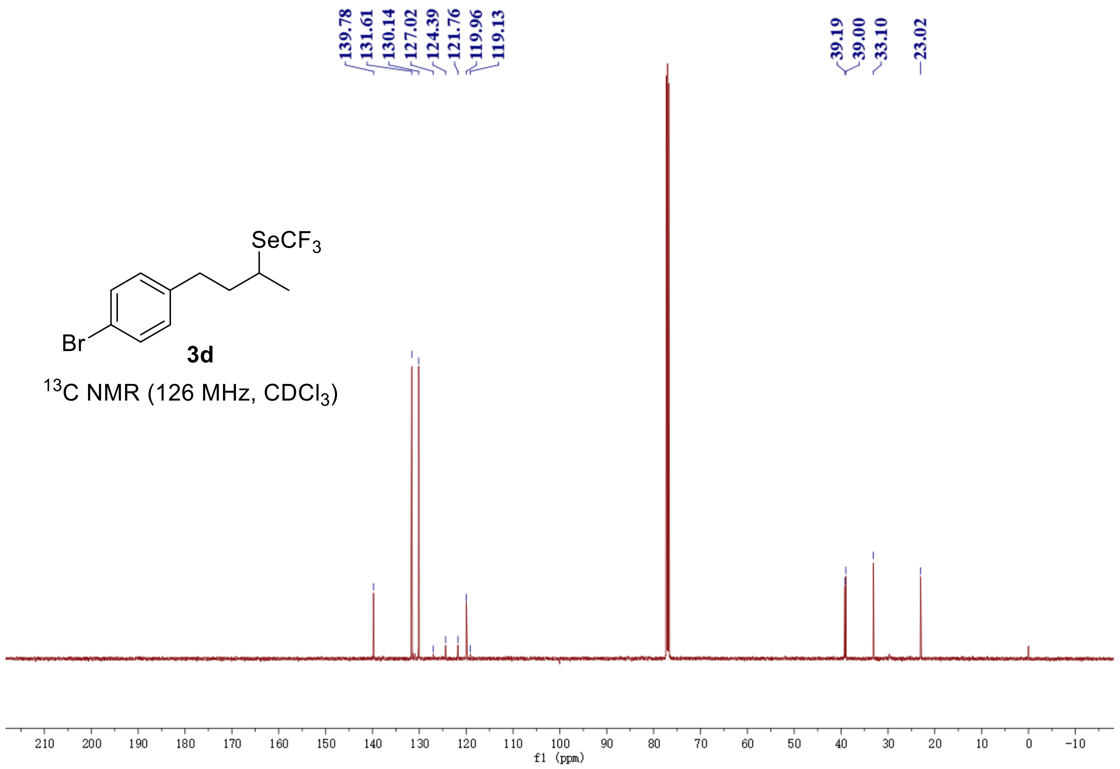

139.78
131.61
130.14
127.02
124.39
121.76
119.96
119.13
39.19
39.00
33.10
23.02
SeCF3
Br
3d
13C NMR (126 MHz, CDCl3)
f1 (ppm)

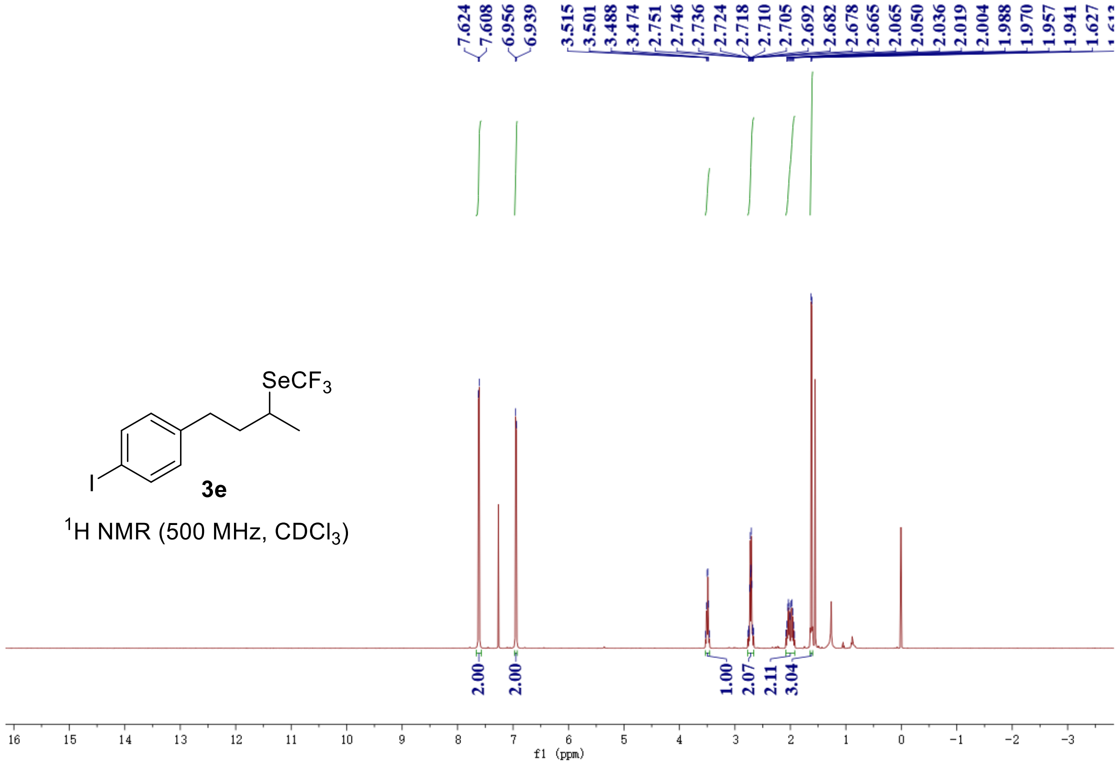

3e

^{1}H NMR (500 MHz, $CDCl_3$)

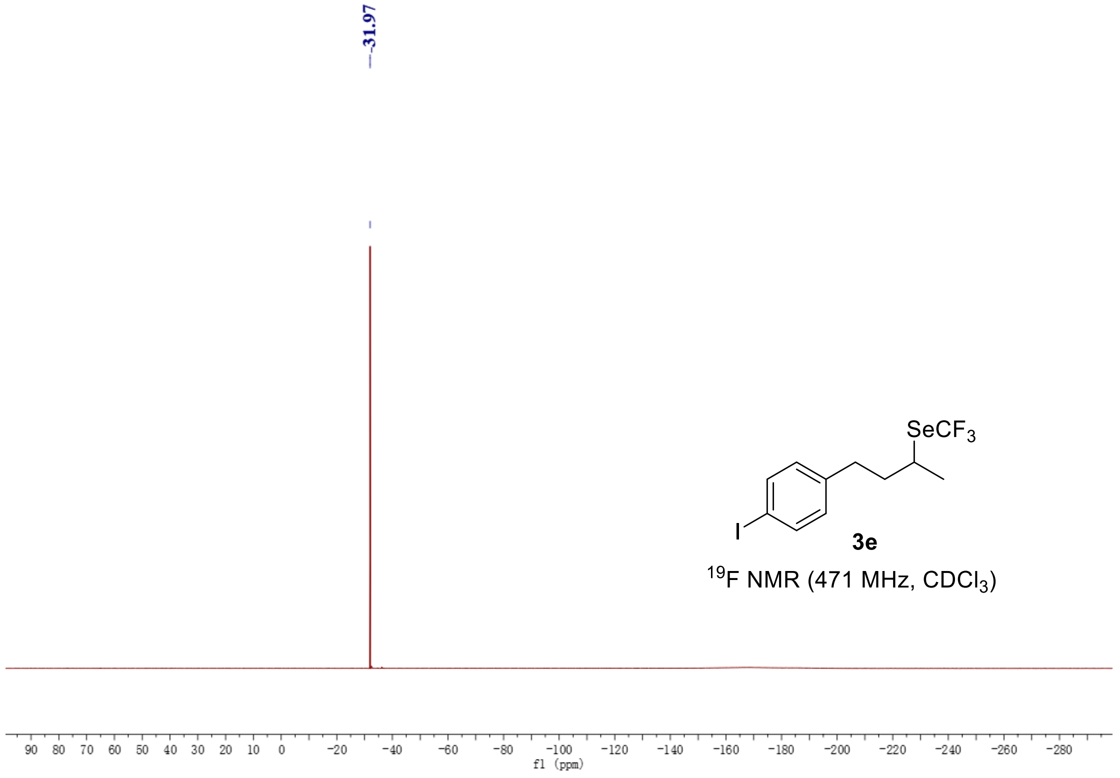

3e

^{19}F NMR (471 MHz, $CDCl_3$)

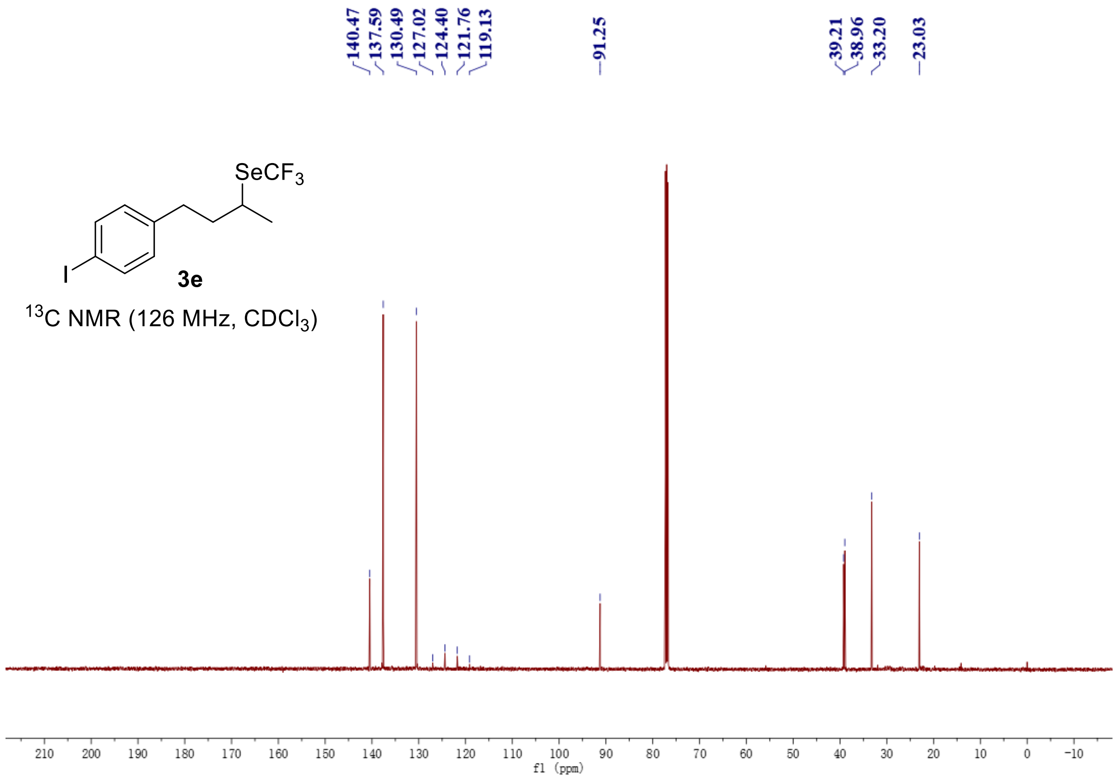
140.47
137.59
130.49
127.02
124.40
121.76
119.13
91.25
39.21
38.96
33.20
23.03
SeCF3
I
3e
13C NMR (126 MHz, CDCl3)
f1 (ppm)

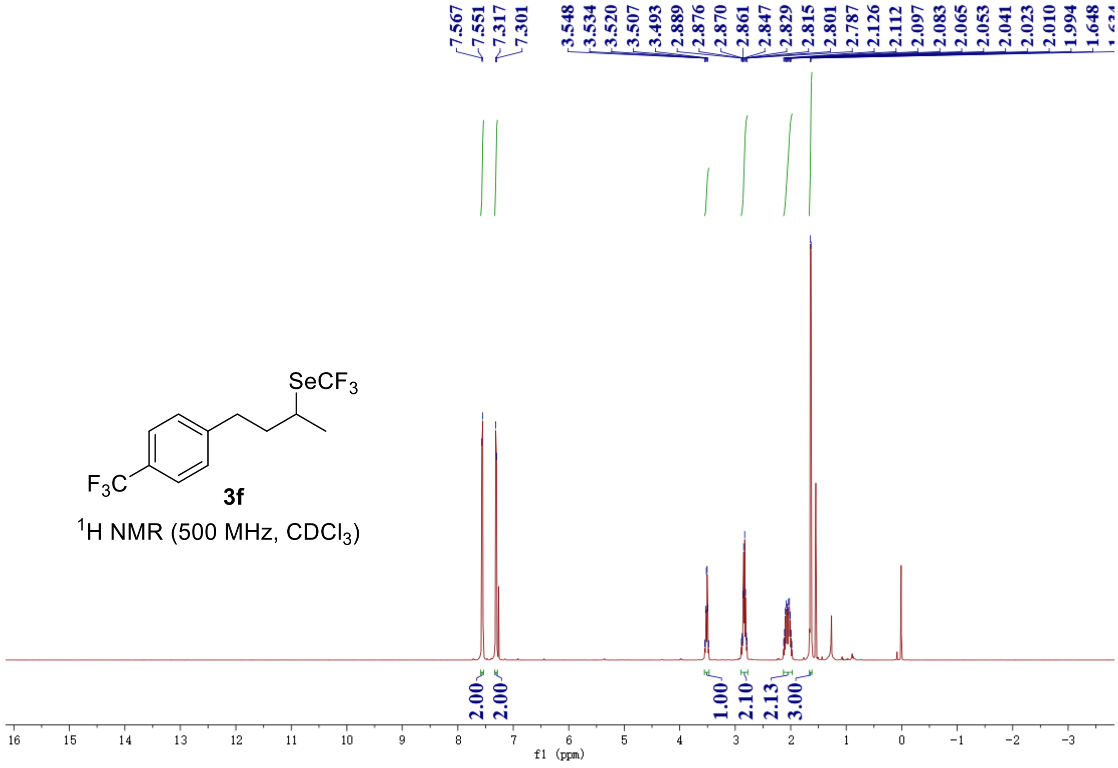
7.567
7.551
7.317
7.301
3.548
3.534
3.520
3.507
3.493
2.889
2.876
2.870
2.861
2.847
2.829
2.815
2.801
2.787
2.126
2.112
2.097
2.083
2.065
2.053
2.041
2.023
2.010
1.994
1.648
SeCF3
F3C
3f
1H NMR (500 MHz, CDCl3)
2.00
2.00
1.00
2.10
2.13
3.00
f1 (ppm)

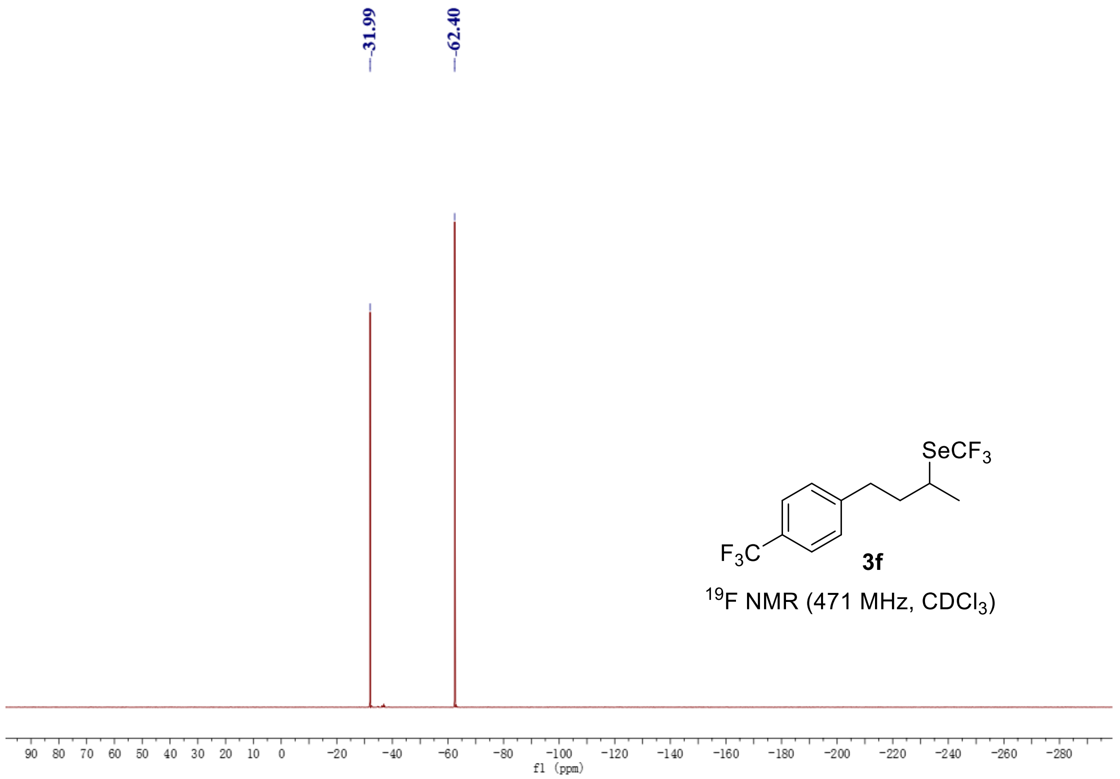

−31.99
−62.40
SeCF3
F3C
3f
19F NMR (471 MHz, CDCl3)
90 80 70 60 50 40 30 20 10 0 −20 −40 −60 −80 −100 −120 −140 −160 −180 −200 −220 −240 −260 −280
f1 (ppm)

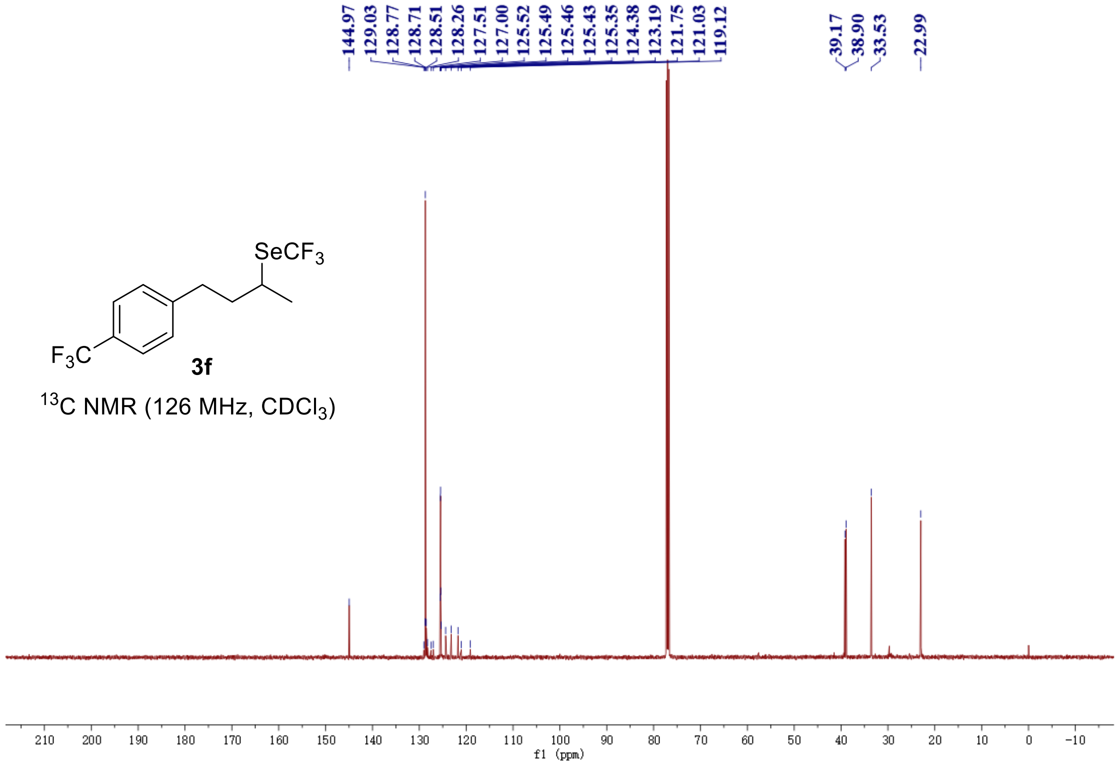

144.97
129.03
128.77
128.71
128.51
128.26
127.51
127.00
125.52
125.49
125.46
125.43
125.35
124.38
123.19
121.75
121.03
119.12
39.17
38.90
33.53
22.99
SeCF3
F3C
3f
13C NMR (126 MHz, CDCl3)
210 200 190 180 170 160 150 140 130 120 110 100 90 80 70 60 50 40 30 20 10 0 −10
f1 (ppm)

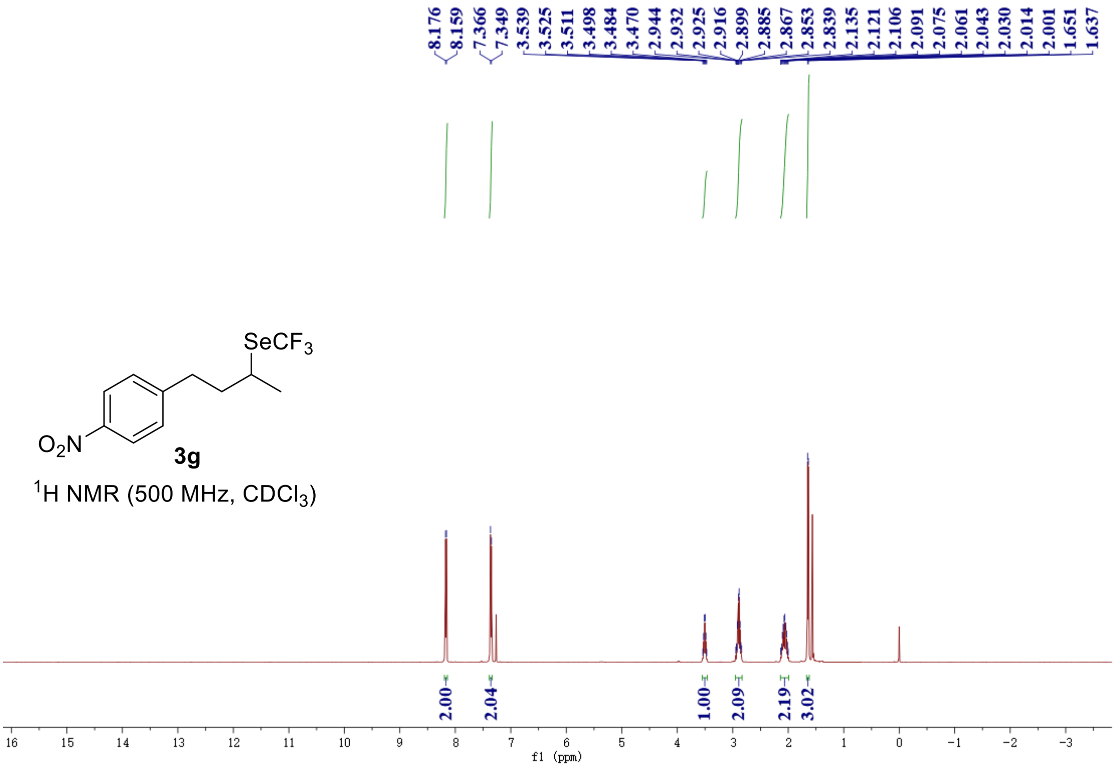
8.176
8.159
7.366
7.349
3.539
3.525
3.511
3.498
3.484
3.470
2.944
2.932
2.925
2.916
2.899
2.885
2.867
2.853
2.839
2.135
2.121
2.106
2.091
2.075
2.061
2.043
2.030
2.014
2.001
1.651
1.637
SeCF3
O2N
3g
1H NMR (500 MHz, CDCl3)
2.00
2.04
1.00
2.09
2.19
3.02
f1 (ppm)

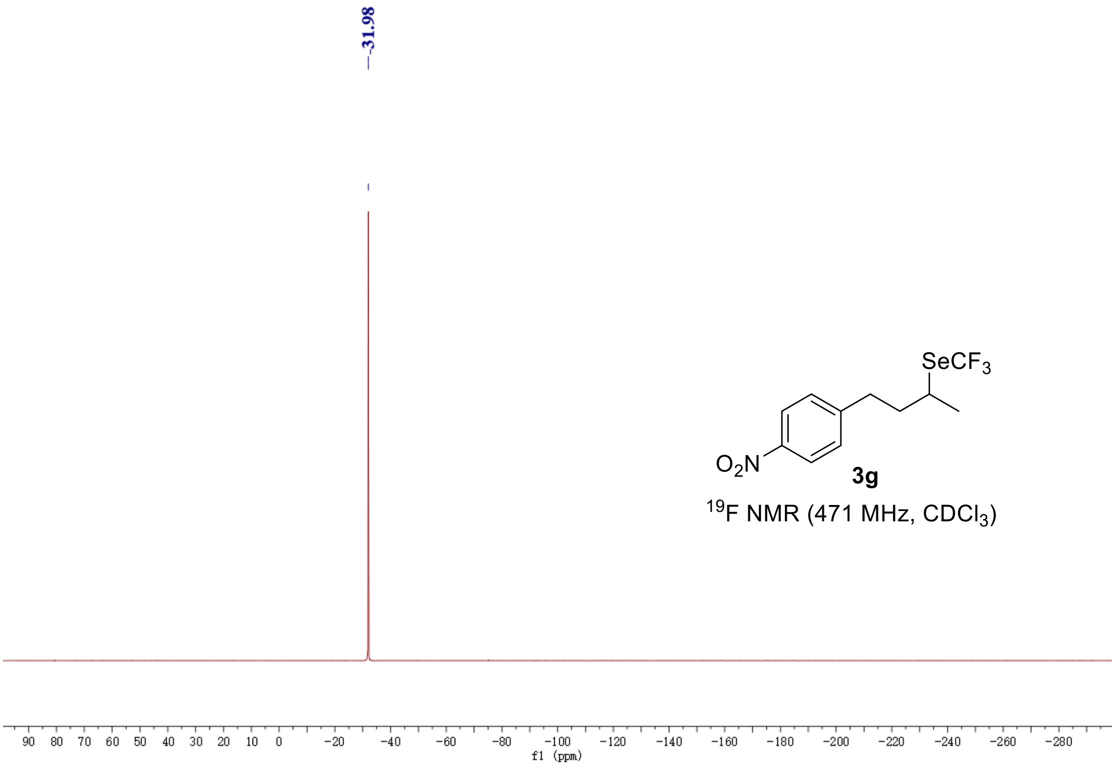
−31.98
SeCF3
O2N
3g
19F NMR (471 MHz, CDCl3)
f1 (ppm)

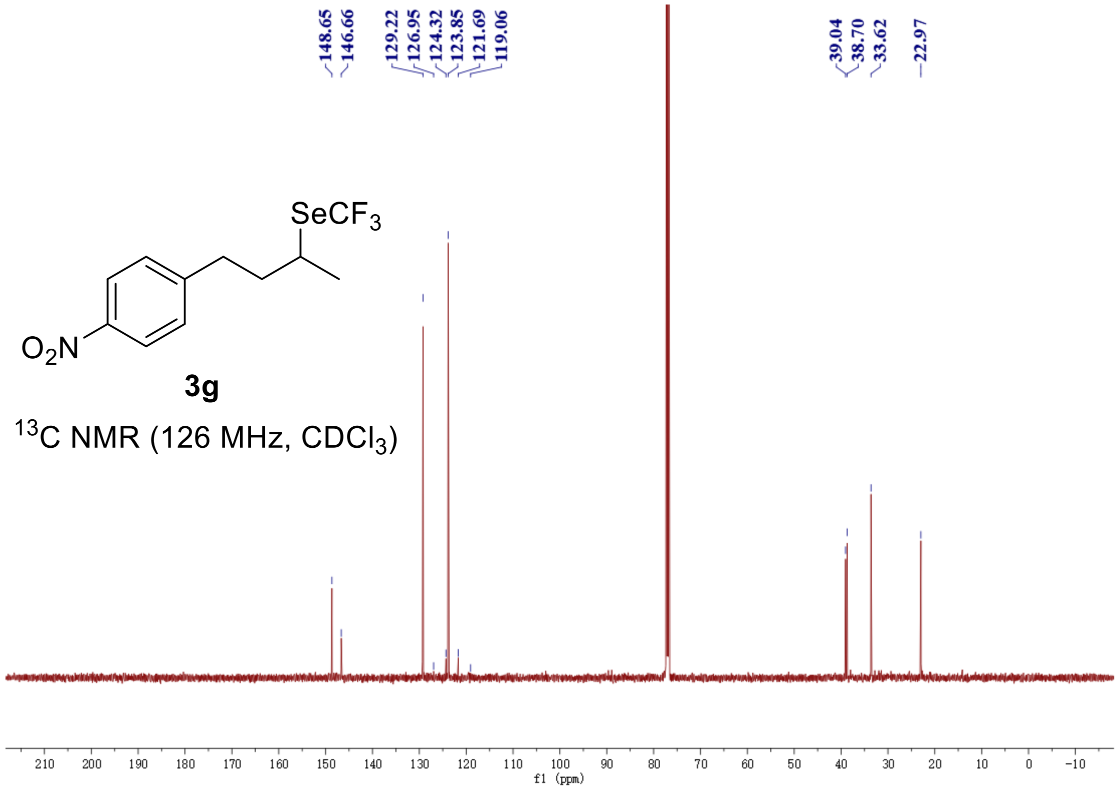

3g

^{13}C NMR (126 MHz, $CDCl_3$)

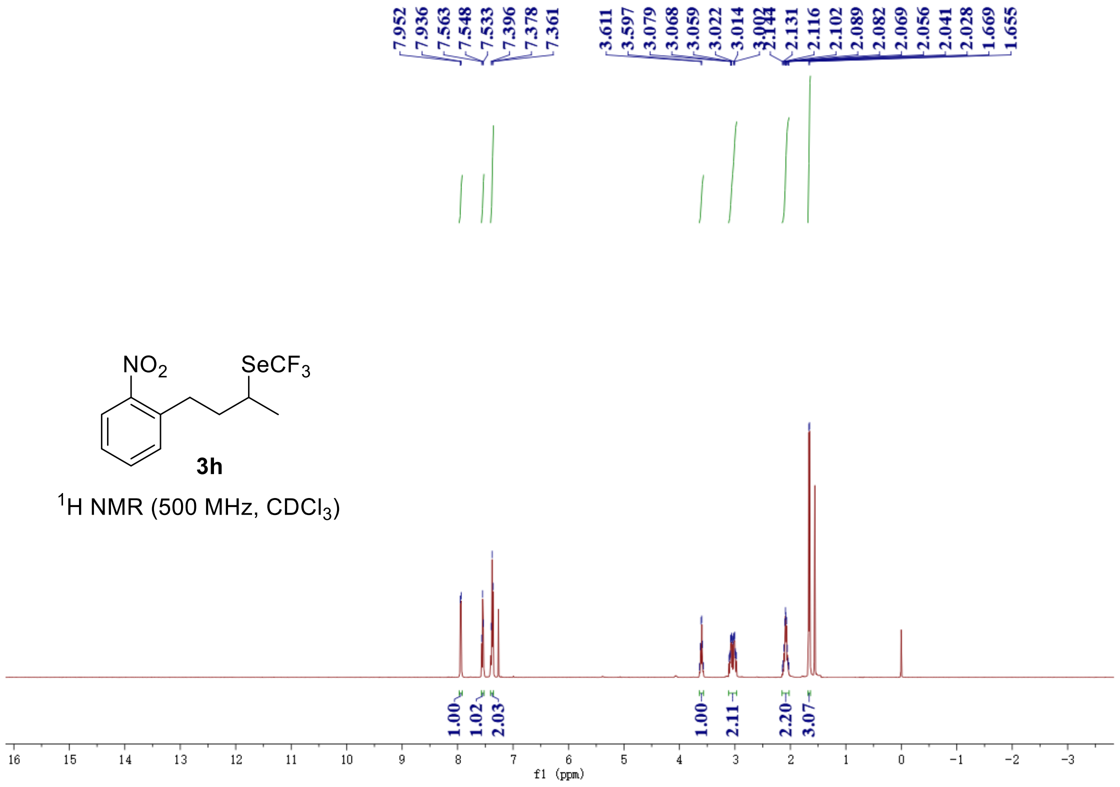

3h

^{1}H NMR (500 MHz, $CDCl_3$)

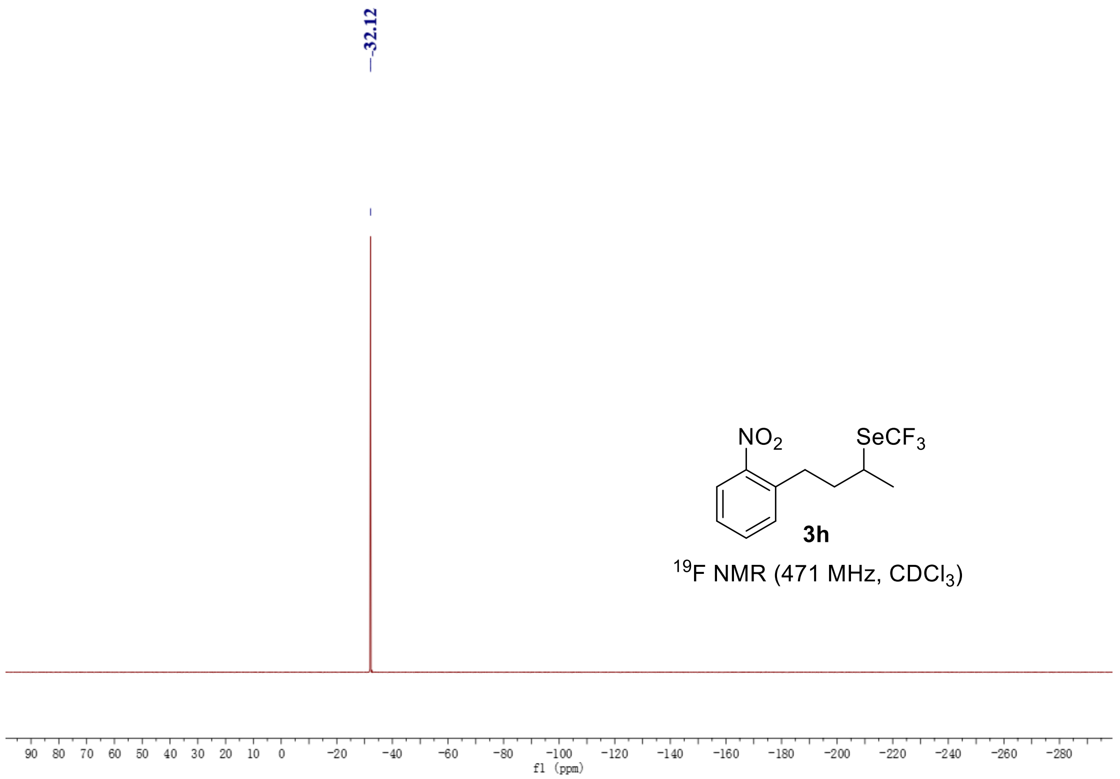

-32.12
NO_2
$SeCF_3$
3h
^{19}F NMR (471 MHz, $CDCl_3$)
90 80 70 60 50 40 30 20 10 0 -20 -40 -60 -80 -100 -120 -140 -160 -180 -200 -220 -240 -260 -280
f1 (ppm)

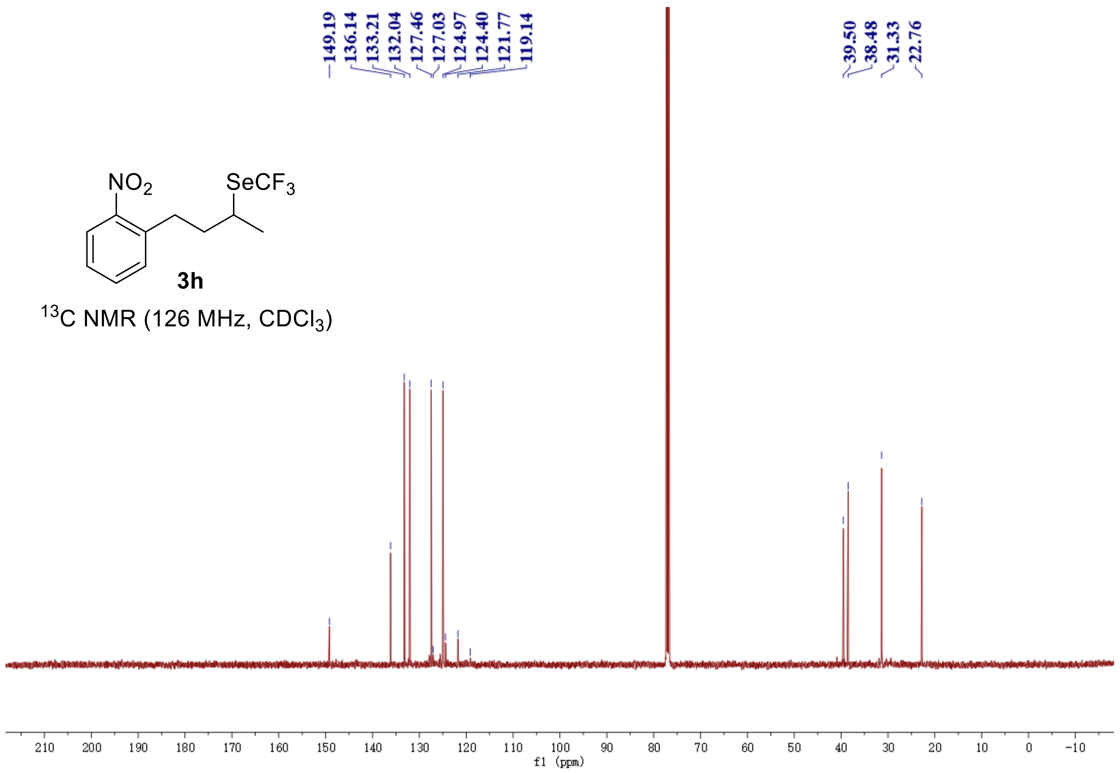

149.19
136.14
133.21
132.04
127.46
127.03
124.97
124.40
121.77
119.14
39.50
38.48
31.33
22.76
NO_2
$SeCF_3$
3h
^{13}C NMR (126 MHz, $CDCl_3$)
210 200 190 180 170 160 150 140 130 120 110 100 90 80 70 60 50 40 30 20 10 0 -10
f1 (ppm)

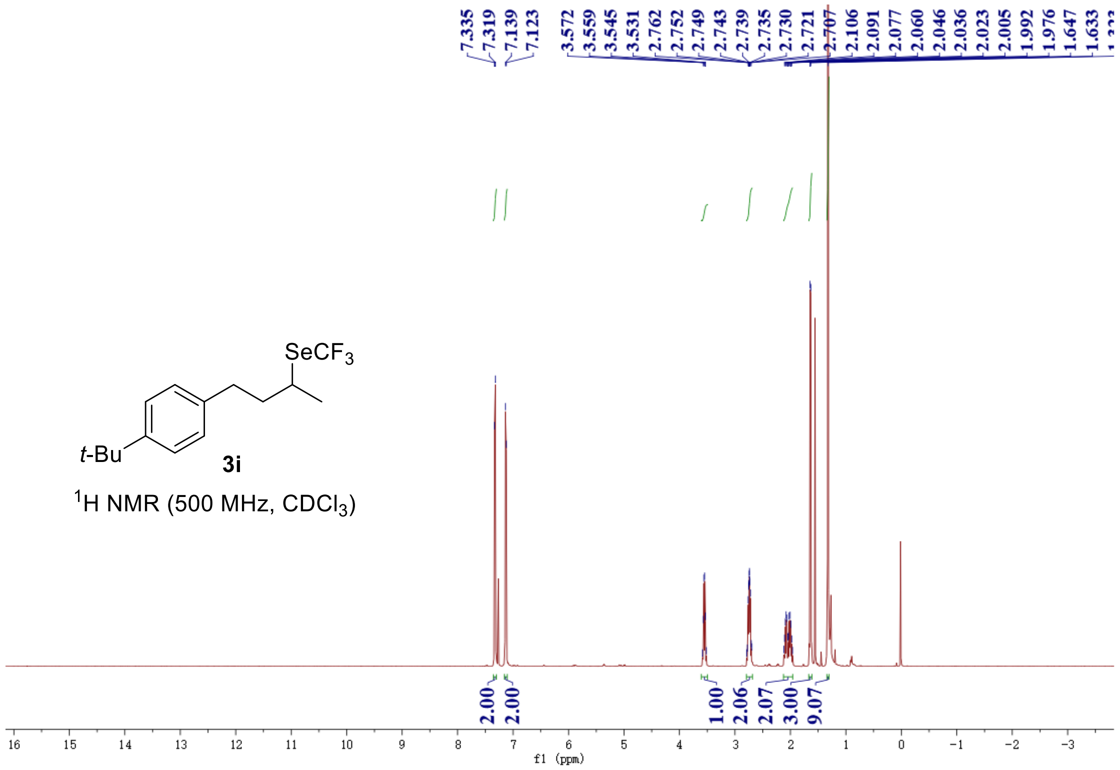
7.335
7.319
7.139
7.123
3.572
3.559
3.545
3.531
2.762
2.752
2.749
2.743
2.739
2.735
2.730
2.721
2.707
2.106
2.091
2.077
2.060
2.046
2.036
2.023
2.005
1.992
1.976
1.647
1.633
SeCF3
t-Bu
3i
1H NMR (500 MHz, CDCl3)
2.00
2.00
1.00
2.06
2.07
3.00
9.07
16 15 14 13 12 11 10 9 8 7 6 5 4 3 2 1 0 -1 -2 -3
f1 (ppm)

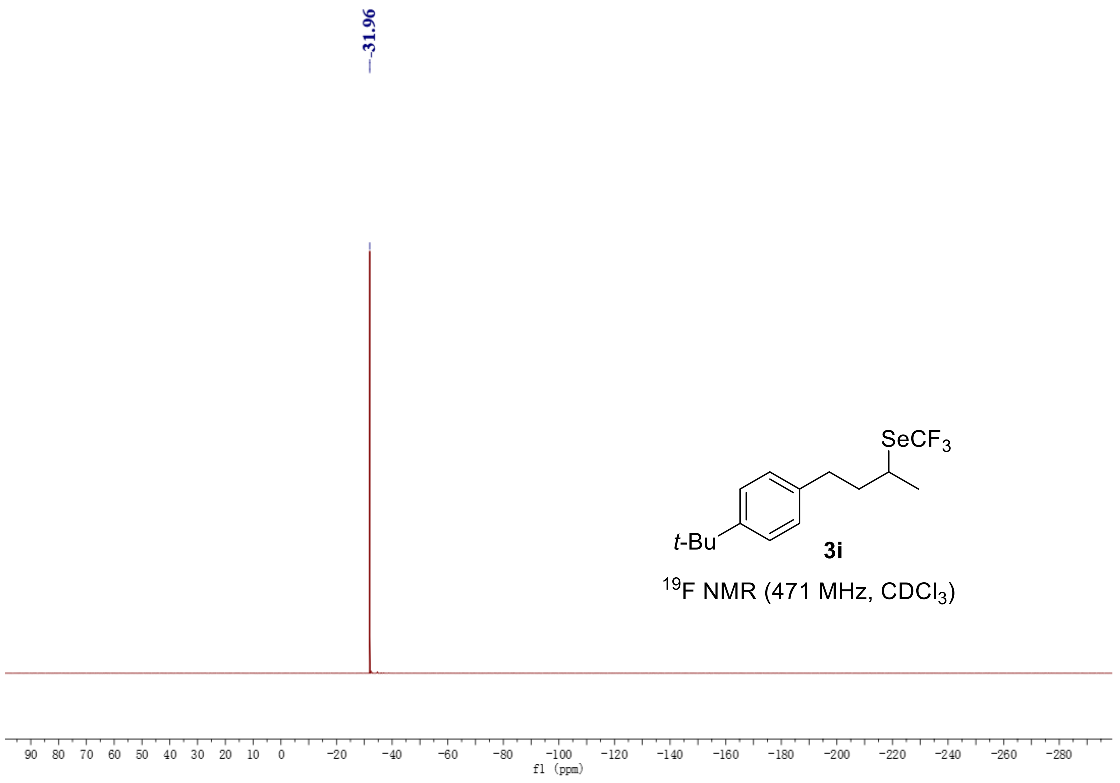
-31.96
SeCF3
t-Bu
3i
19F NMR (471 MHz, CDCl3)
90 80 70 60 50 40 30 20 10 0 -20 -40 -60 -80 -100 -120 -140 -160 -180 -200 -220 -240 -260 -280
f1 (ppm)

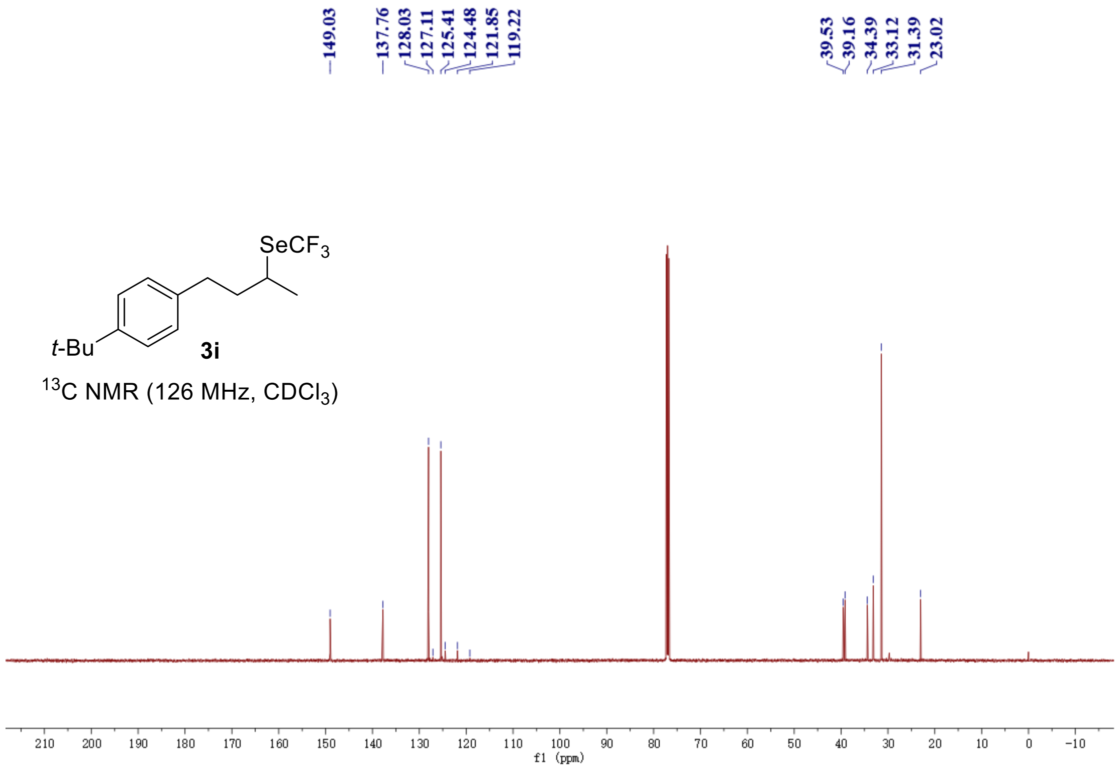
149.03
137.76
128.03
127.11
125.41
124.48
121.85
119.22
39.53
39.16
34.39
33.12
31.39
23.02
SeCF3
t-Bu
3i
13C NMR (126 MHz, CDCl3)
f1 (ppm)

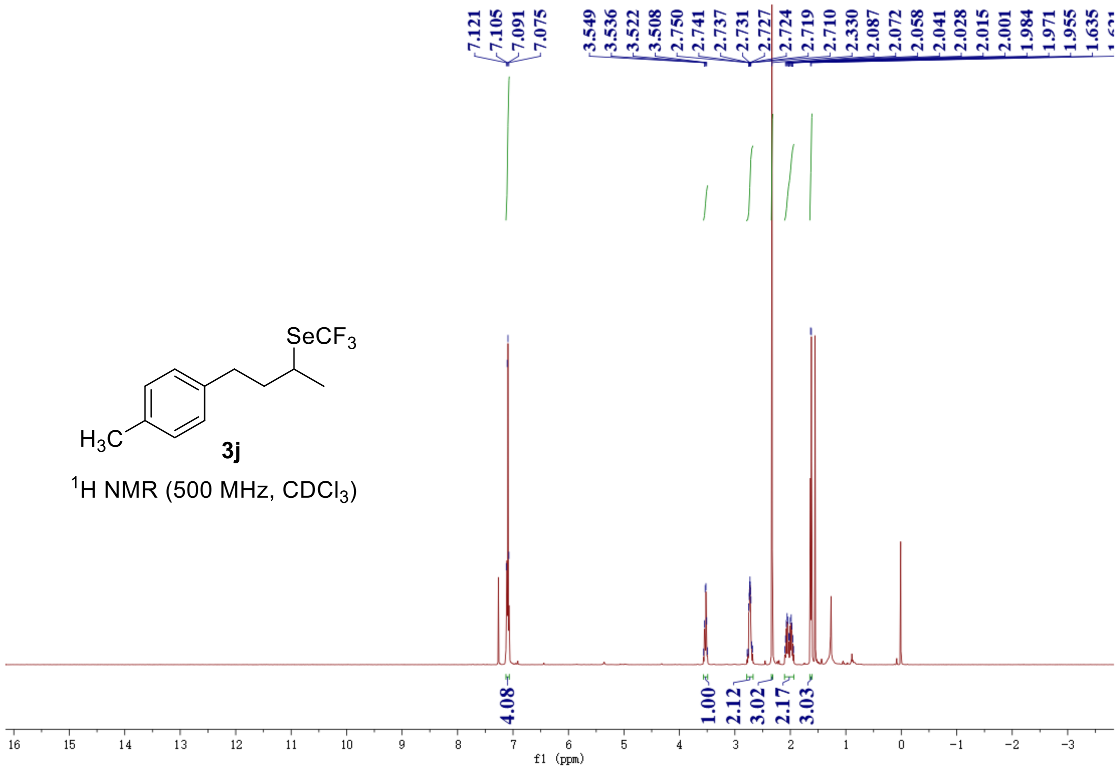
7.121
7.105
7.091
7.075
3.549
3.536
3.522
3.508
2.750
2.741
2.737
2.731
2.727
2.724
2.719
2.710
2.330
2.087
2.072
2.058
2.041
2.028
2.015
2.001
1.984
1.971
1.955
1.635
SeCF3
H3C
3j
1H NMR (500 MHz, CDCl3)
4.08
1.00
2.12
3.02
2.17
3.03
f1 (ppm)

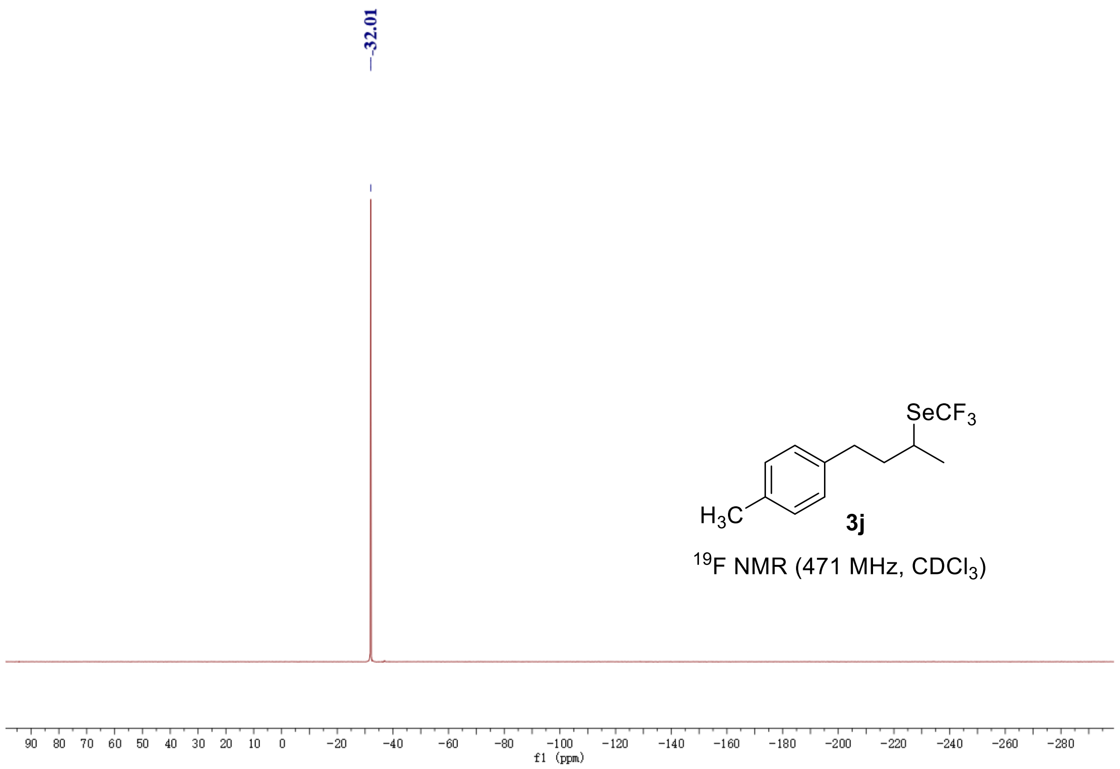
—32.01
SeCF3
H3C
3j
19F NMR (471 MHz, CDCl3)
90 80 70 60 50 40 30 20 10 0 −20 −40 −60 −80 −100 −120 −140 −160 −180 −200 −220 −240 −260 −280
f1 (ppm)

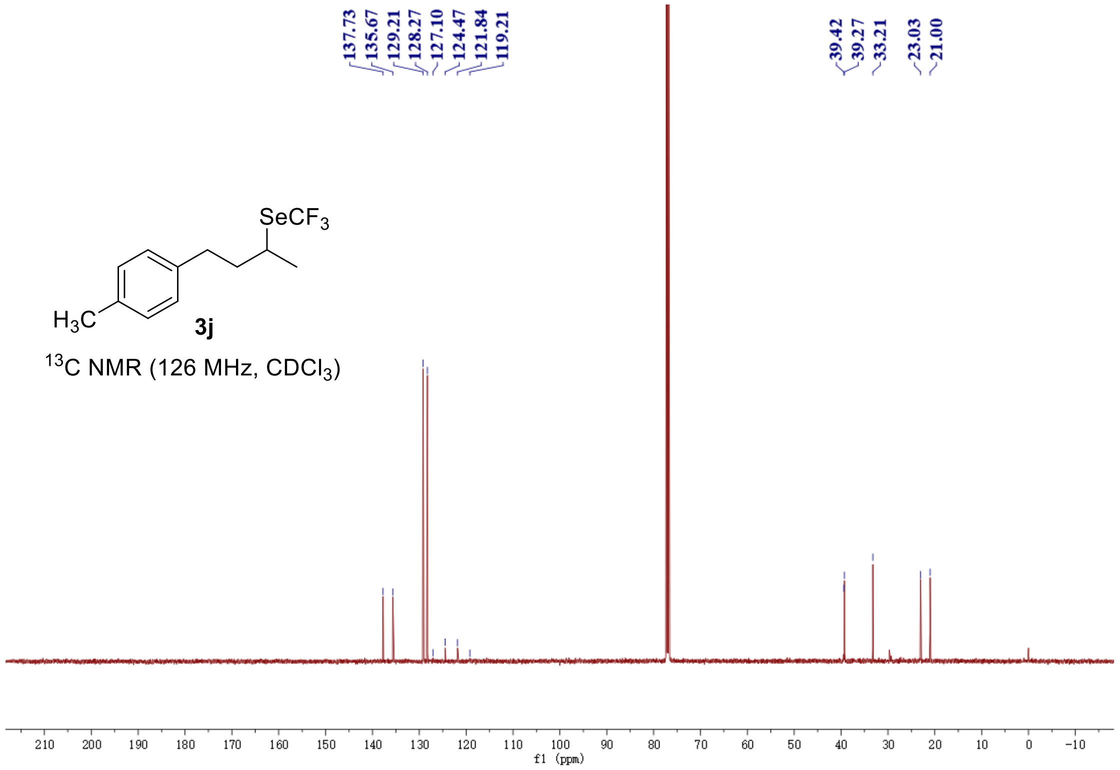
137.73
135.67
129.21
128.27
127.10
124.47
121.84
119.21
39.42
39.27
33.21
23.03
21.00
SeCF3
H3C
3j
13C NMR (126 MHz, CDCl3)
210 200 190 180 170 160 150 140 130 120 110 100 90 80 70 60 50 40 30 20 10 0 −10
f1 (ppm)

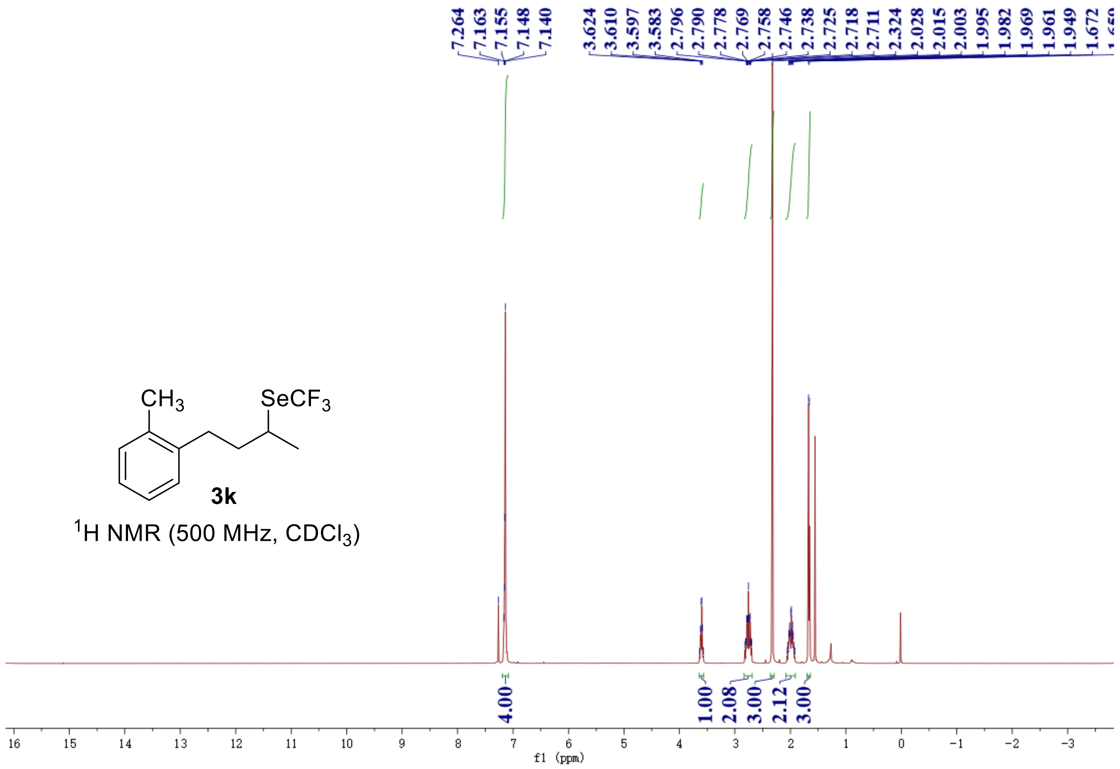
7.264
7.163
7.155
7.148
7.140
3.624
3.610
3.597
3.583
2.796
2.790
2.778
2.769
2.758
2.746
2.738
2.725
2.718
2.711
2.324
2.028
2.015
2.003
1.995
1.982
1.969
1.961
1.949
1.672
CH3
SeCF3
3k
1H NMR (500 MHz, CDCl3)
4.00
1.00
2.08
3.00
2.12
3.00
f1 (ppm)

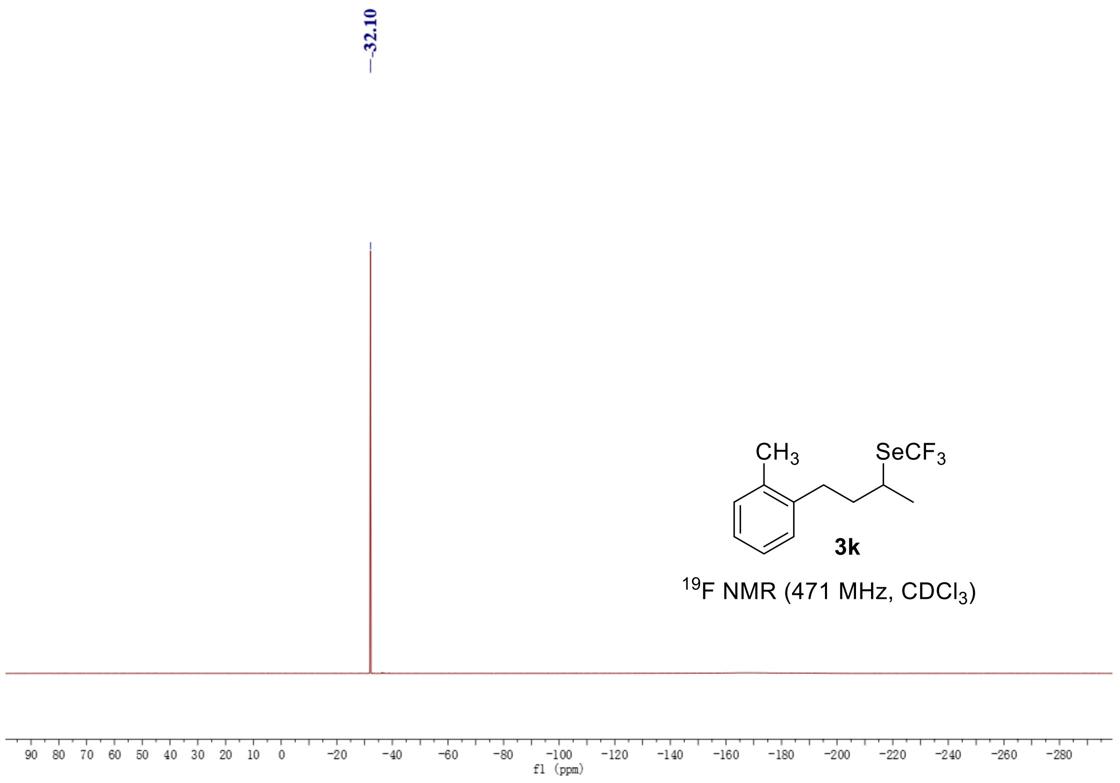
-32.10
CH3
SeCF3
3k
19F NMR (471 MHz, CDCl3)
f1 (ppm)

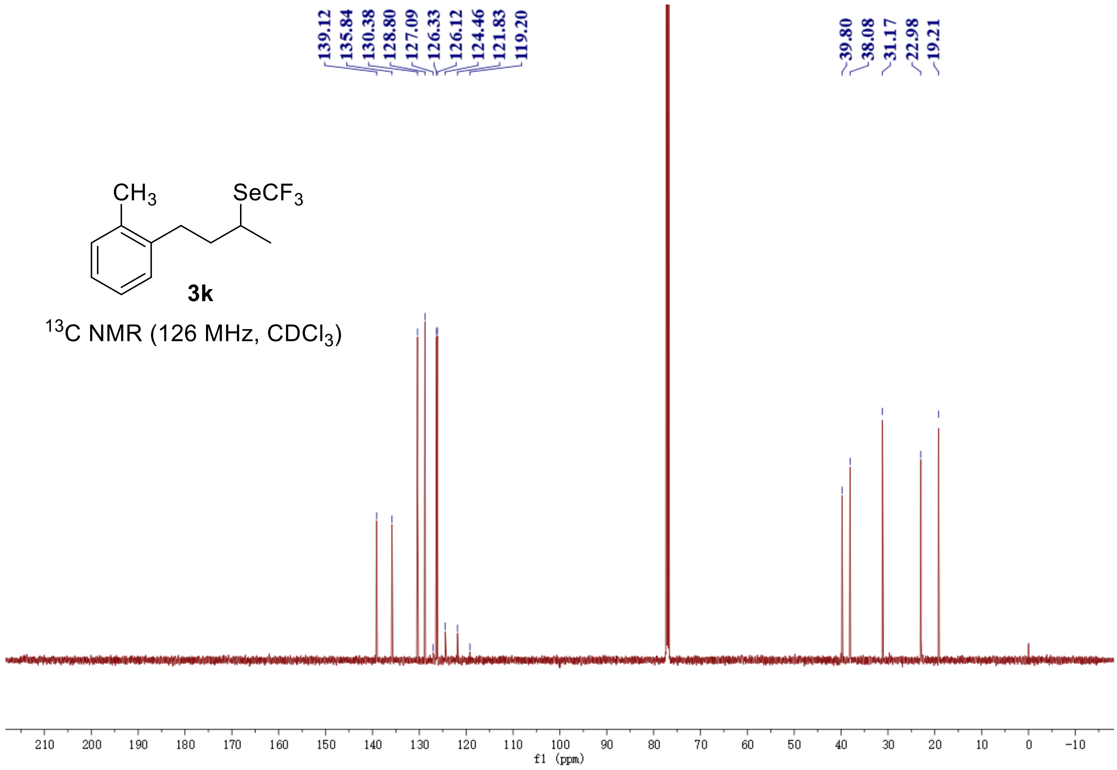

139.12
135.84
130.38
128.80
127.09
126.33
126.12
124.46
121.83
119.20
39.80
38.08
31.17
22.98
19.21
CH3
SeCF3
3k
13C NMR (126 MHz, CDCl3)
f1 (ppm)

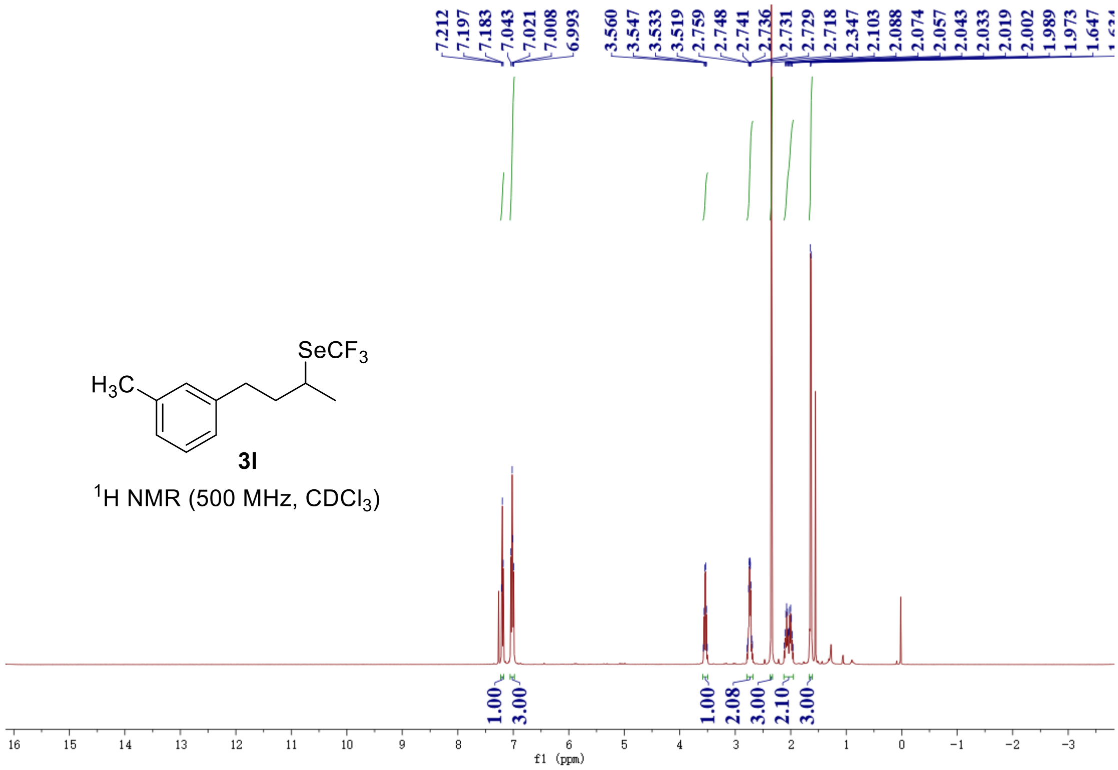

7.212
7.197
7.183
7.043
7.021
7.008
6.993
3.560
3.547
3.533
3.519
2.759
2.748
2.741
2.736
2.731
2.729
2.718
2.347
2.103
2.088
2.074
2.057
2.043
2.033
2.019
2.002
1.989
1.973
1.647
H3C
SeCF3
3l
1H NMR (500 MHz, CDCl3)
1.00
3.00
1.00
2.08
3.00
2.10
3.00
f1 (ppm)

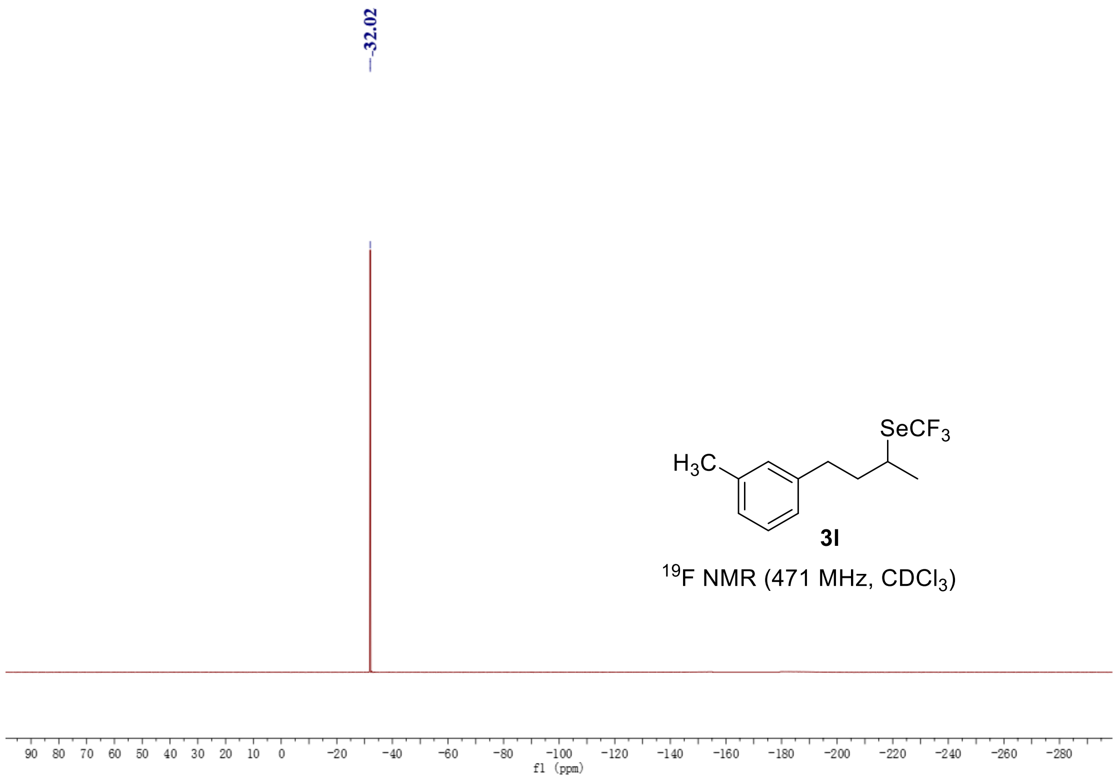
-32.02
SeCF3
H3C
3l
19F NMR (471 MHz, CDCl3)
90 80 70 60 50 40 30 20 10 0 -20 -40 -60 -80 -100 -120 -140 -160 -180 -200 -220 -240 -260 -280
f1 (ppm)

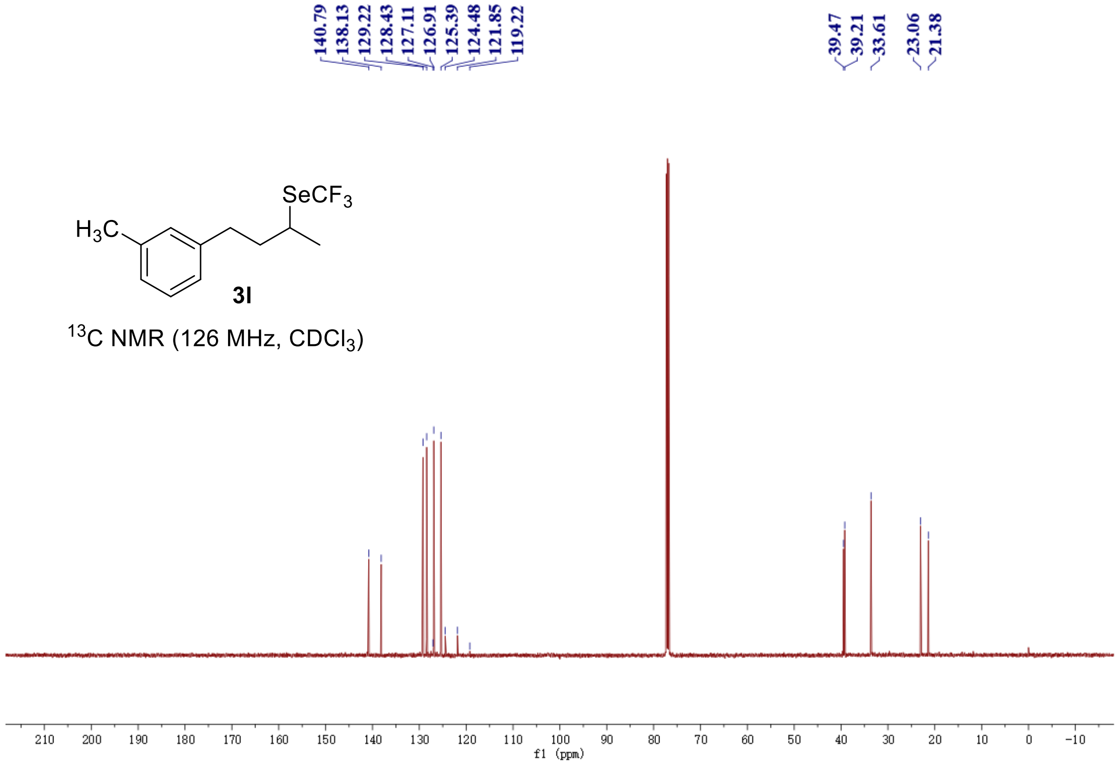
140.79
138.13
129.22
128.43
127.11
126.91
125.39
124.48
121.85
119.22
39.47
39.21
33.61
23.06
21.38
SeCF3
H3C
3l
13C NMR (126 MHz, CDCl3)
210 200 190 180 170 160 150 140 130 120 110 100 90 80 70 60 50 40 30 20 10 0 -10
f1 (ppm)

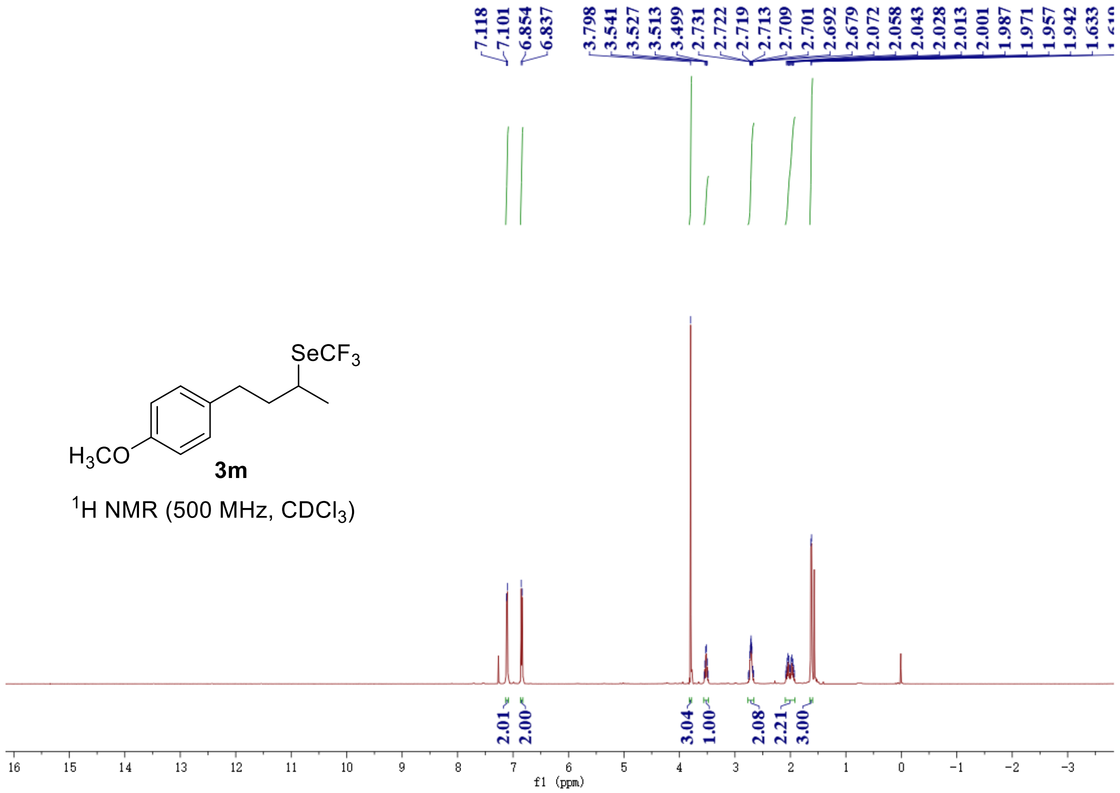

3m

^{1}H NMR (500 MHz, $CDCl_3$)

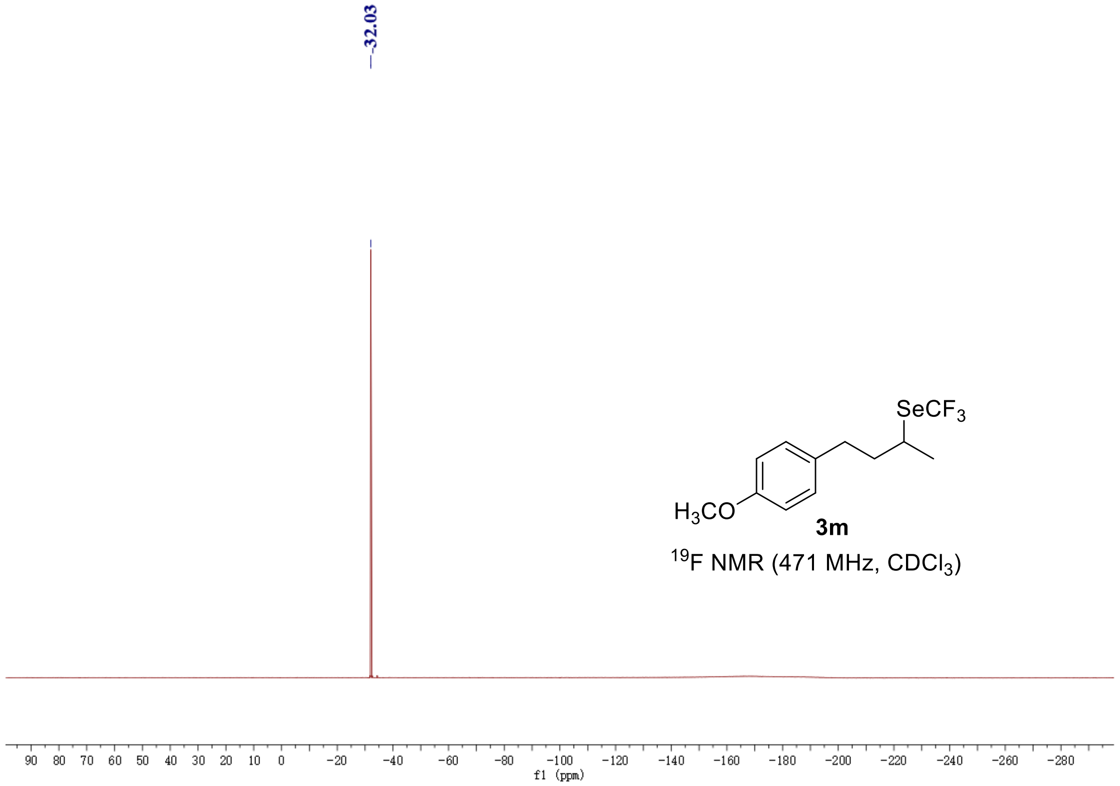

3m

^{19}F NMR (471 MHz, $CDCl_3$)

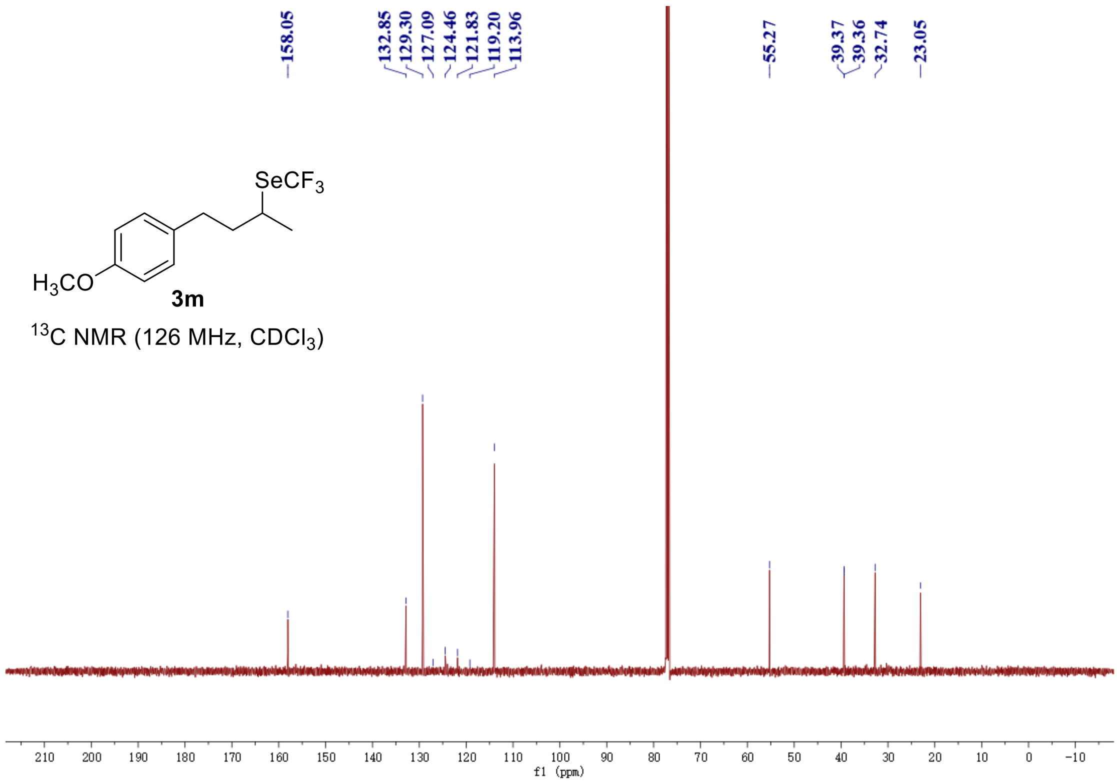

3m

^{13}C NMR (126 MHz, $CDCl_3$)

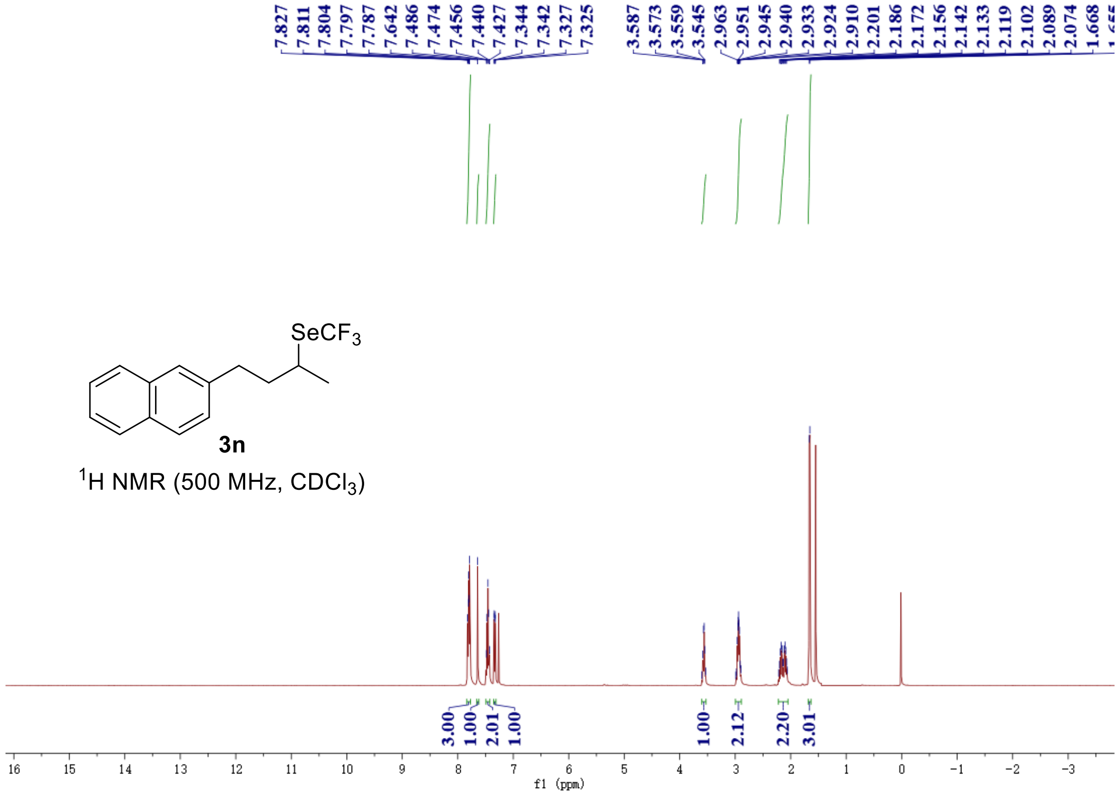

3n

^{1}H NMR (500 MHz, $CDCl_3$)

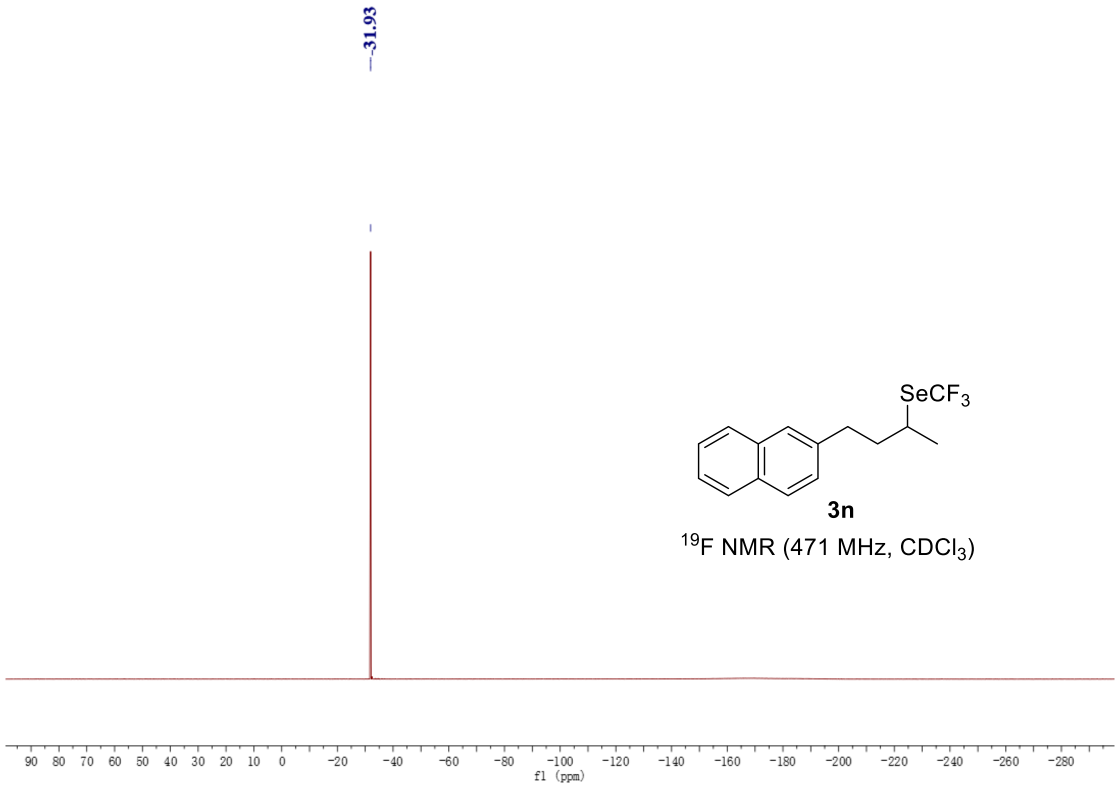

-31.93
SeCF3
3n
19F NMR (471 MHz, CDCl3)
90 80 70 60 50 40 30 20 10 0 -20 -40 -60 -80 -100 -120 -140 -160 -180 -200 -220 -240 -260 -280
f1 (ppm)

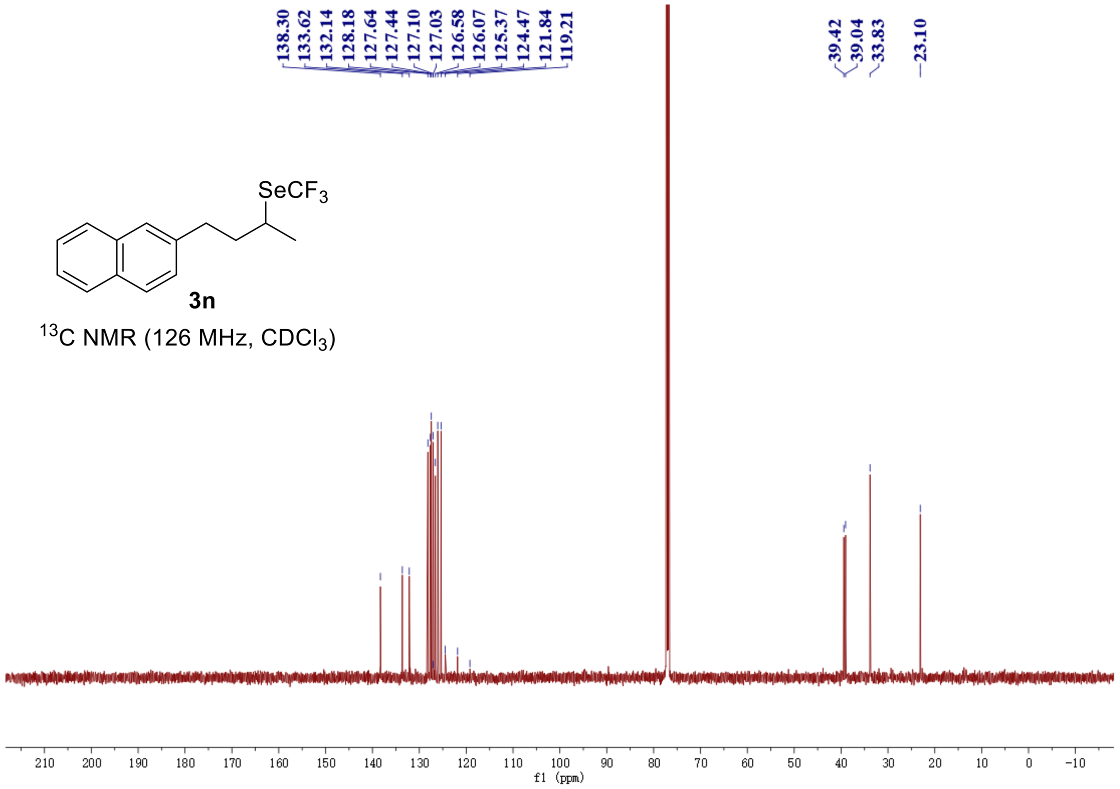

138.30
133.62
132.14
128.18
127.64
127.44
127.10
127.03
126.58
126.07
125.37
124.47
121.84
119.21
39.42
39.04
33.83
23.10
SeCF3
3n
13C NMR (126 MHz, CDCl3)
210 200 190 180 170 160 150 140 130 120 110 100 90 80 70 60 50 40 30 20 10 0 -10
f1 (ppm)

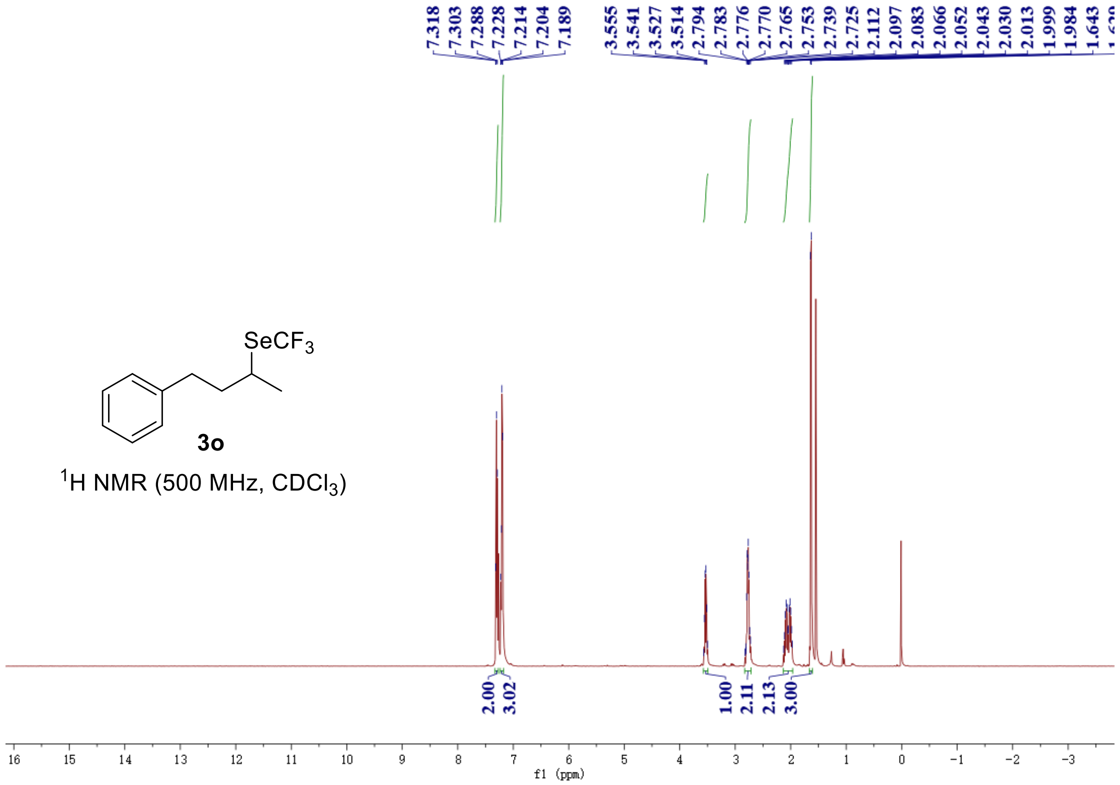
7.318
7.303
7.288
7.228
7.214
7.204
7.189
3.555
3.541
3.527
3.514
2.794
2.783
2.776
2.770
2.765
2.753
2.739
2.725
2.112
2.097
2.083
2.066
2.052
2.043
2.030
2.013
1.999
1.984
1.643
SeCF3
3o
1H NMR (500 MHz, CDCl3)
2.00
3.02
1.00
2.11
2.13
3.00
f1 (ppm)

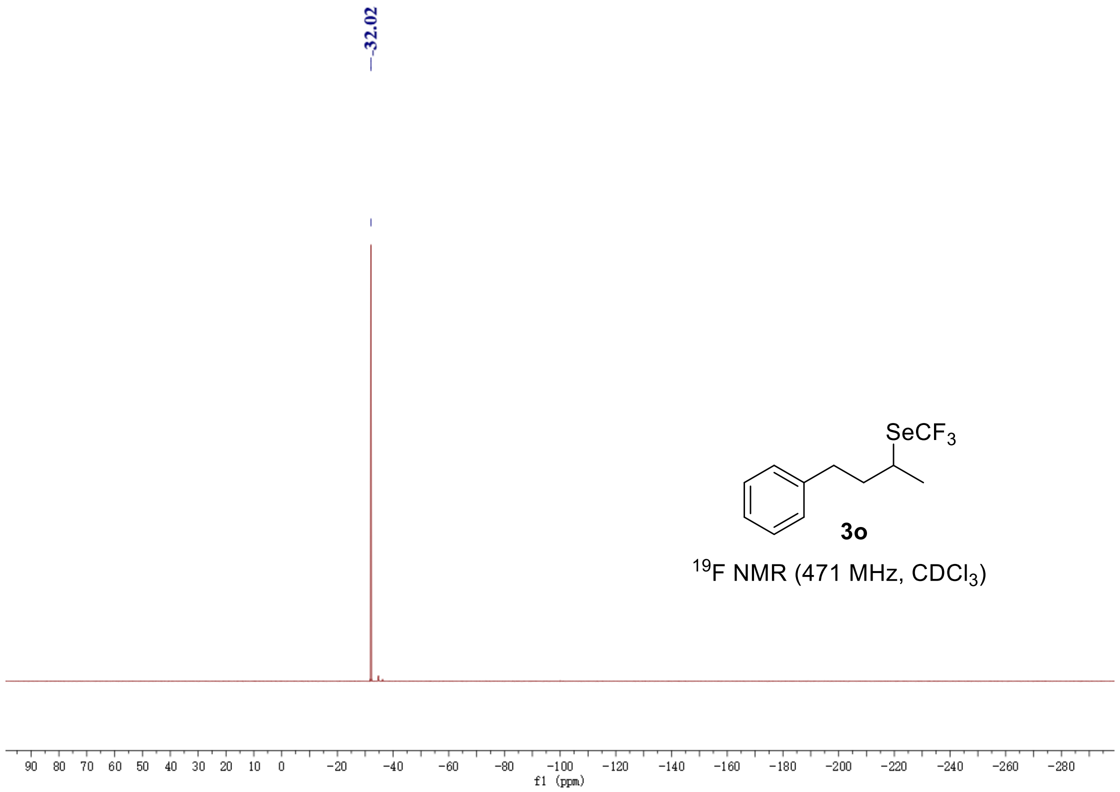
-32.02
SeCF3
3o
19F NMR (471 MHz, CDCl3)
f1 (ppm)

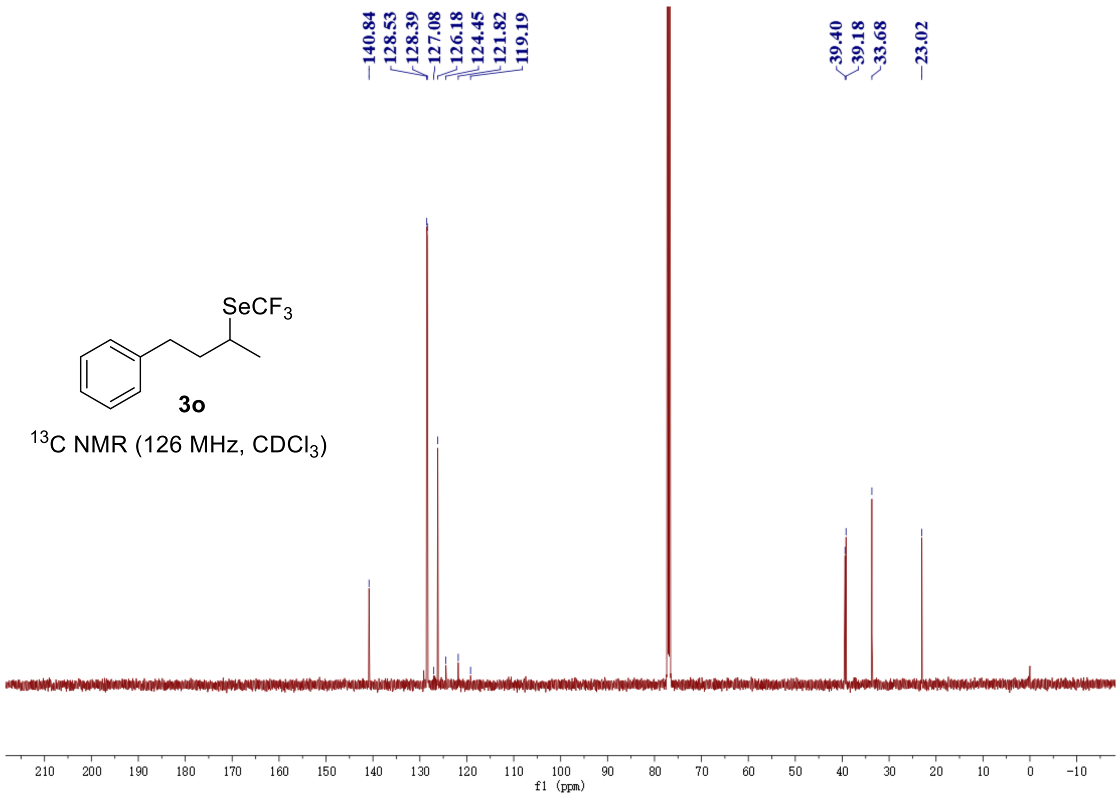

3o

^{13}C NMR (126 MHz, $CDCl_3$)

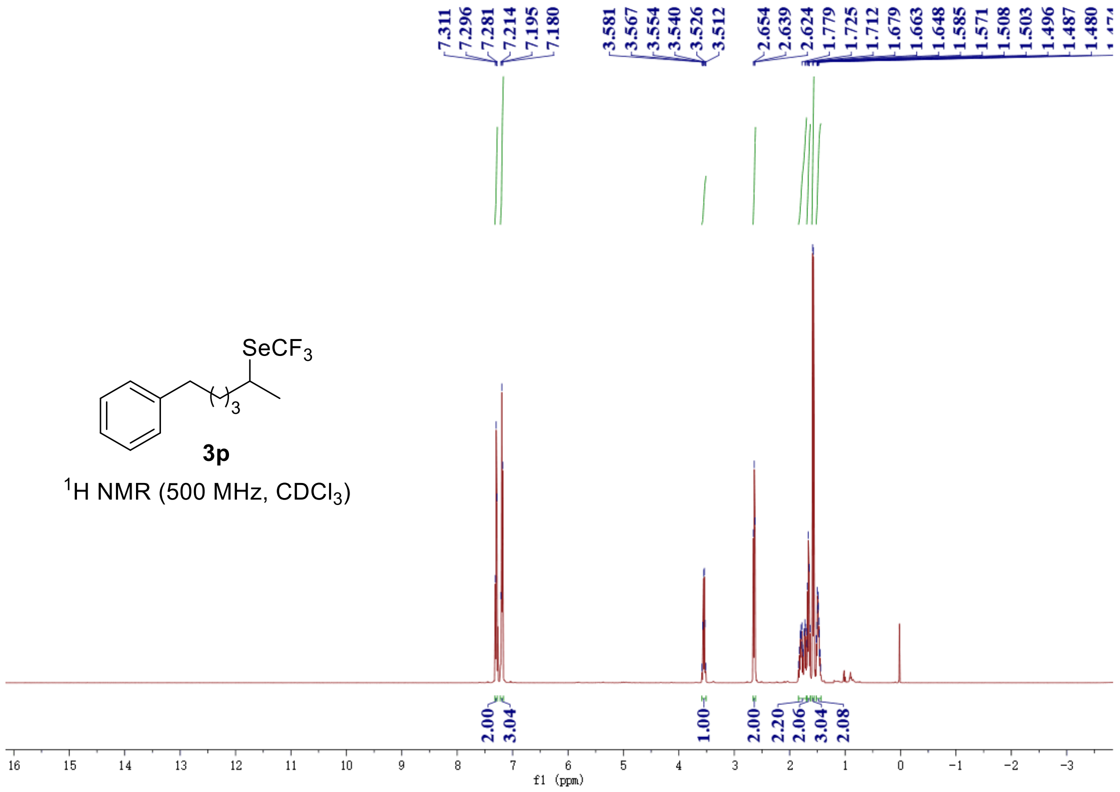

3p

^{1}H NMR (500 MHz, $CDCl_3$)

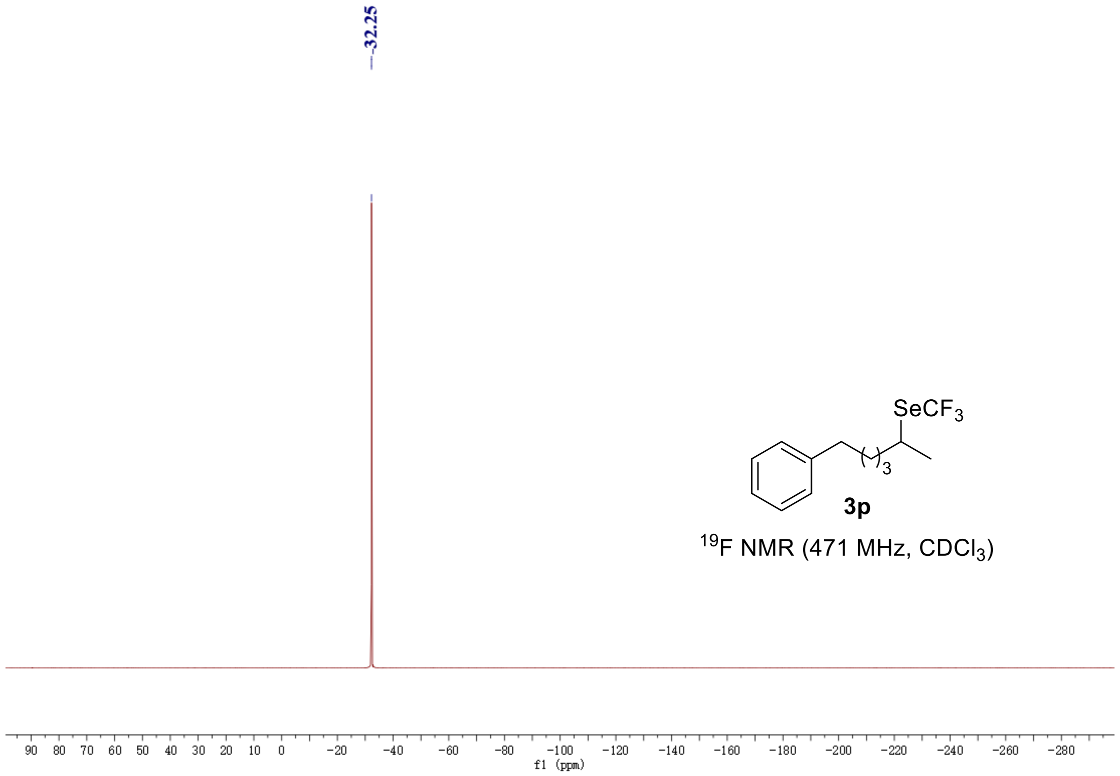
-32.25
SeCF3
3
3p
19F NMR (471 MHz, CDCl3)
90 80 70 60 50 40 30 20 10 0 -20 -40 -60 -80 -100 -120 -140 -160 -180 -200 -220 -240 -260 -280
f1 (ppm)

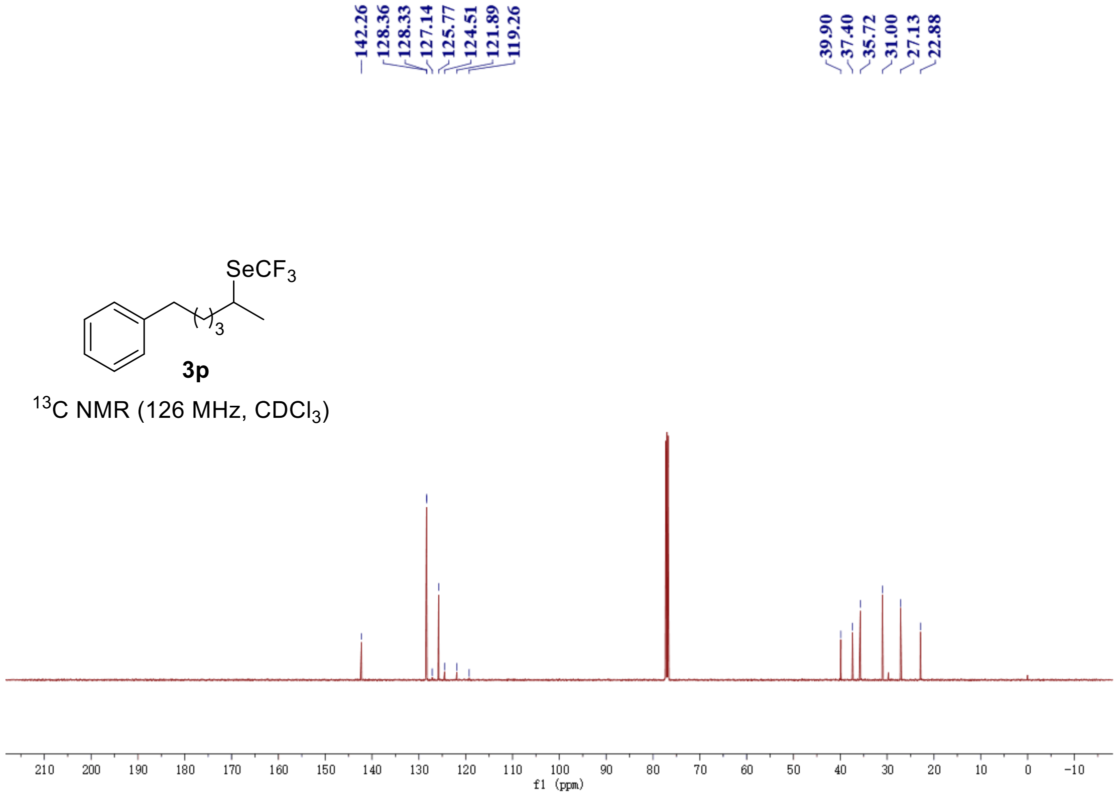
142.26
128.36
128.33
127.14
125.77
124.51
121.89
119.26
39.90
37.40
35.72
31.00
27.13
22.88
SeCF3
3
3p
13C NMR (126 MHz, CDCl3)
210 200 190 180 170 160 150 140 130 120 110 100 90 80 70 60 50 40 30 20 10 0 -10
f1 (ppm)

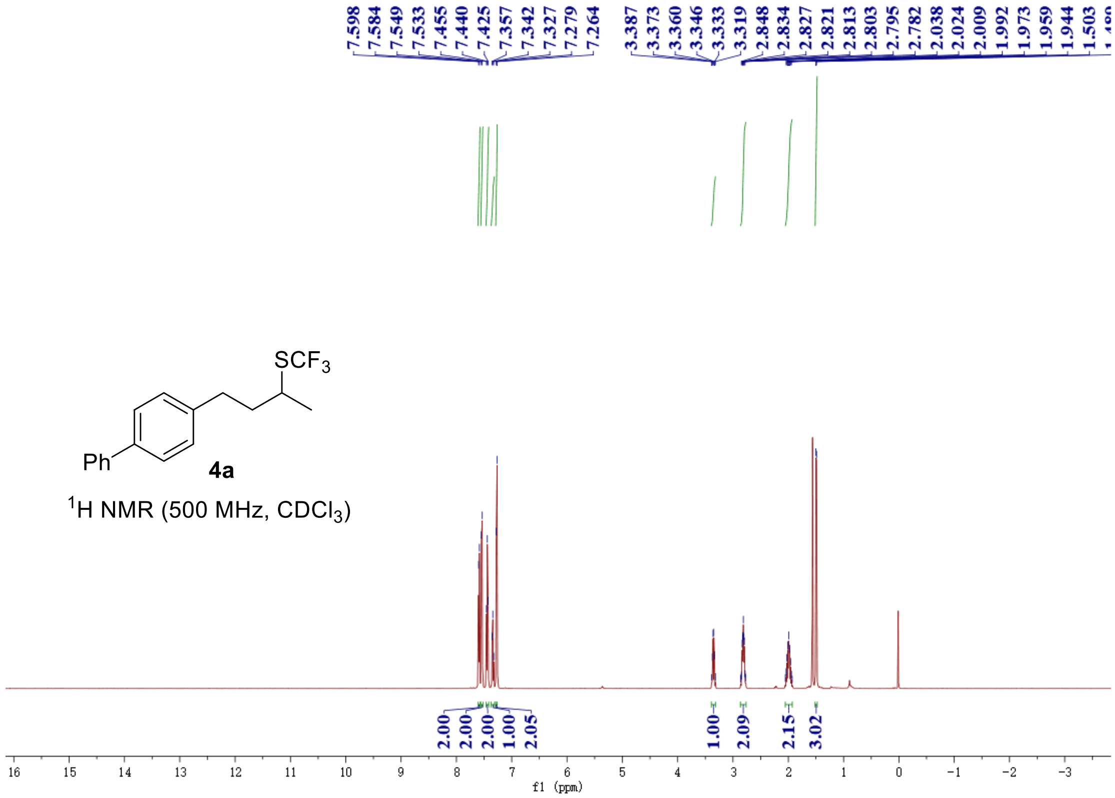
7.598 7.584 7.549 7.533 7.455 7.440 7.425 7.357 7.342 7.327 7.279 7.264 3.387 3.373 3.360 3.346 3.333 3.319 2.848 2.834 2.827 2.821 2.813 2.803 2.795 2.782 2.038 2.024 2.009 1.992 1.973 1.959 1.944 1.503
SCF3
Ph
4a
1H NMR (500 MHz, CDCl3)
2.00 2.00 2.00 1.00 2.05 1.00 2.09 2.15 3.02
f1 (ppm)

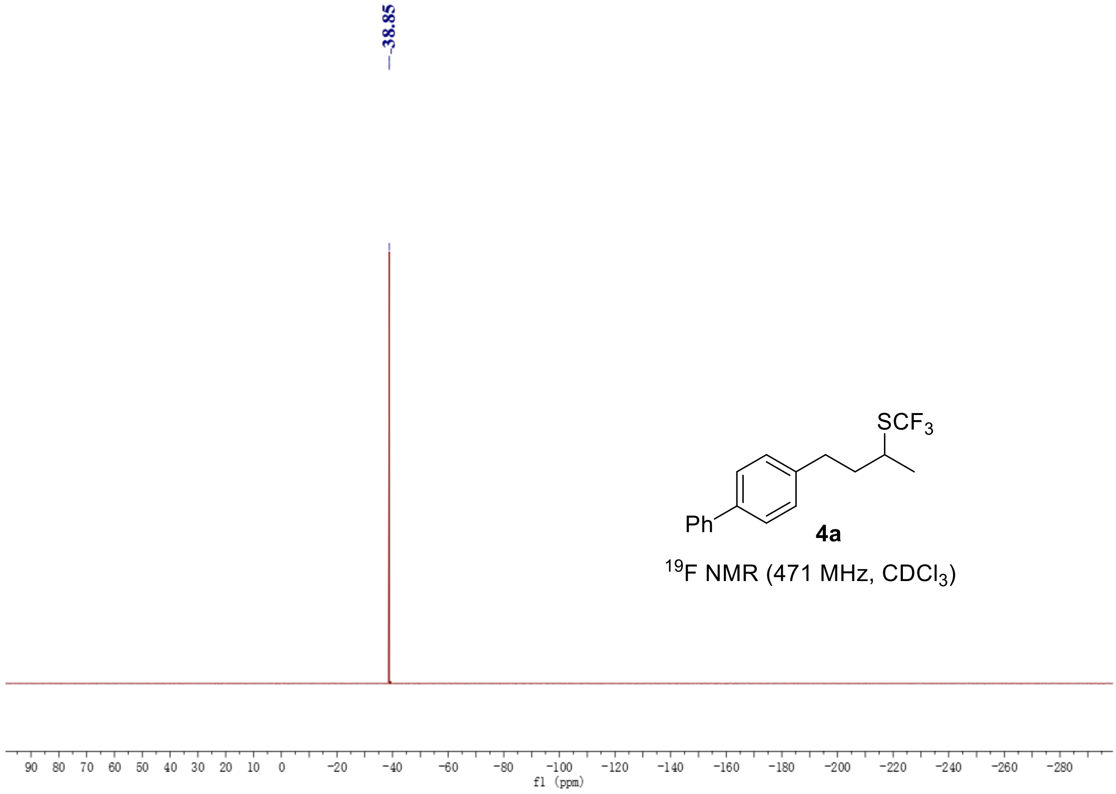
-38.85
SCF3
Ph
4a
19F NMR (471 MHz, CDCl3)
f1 (ppm)

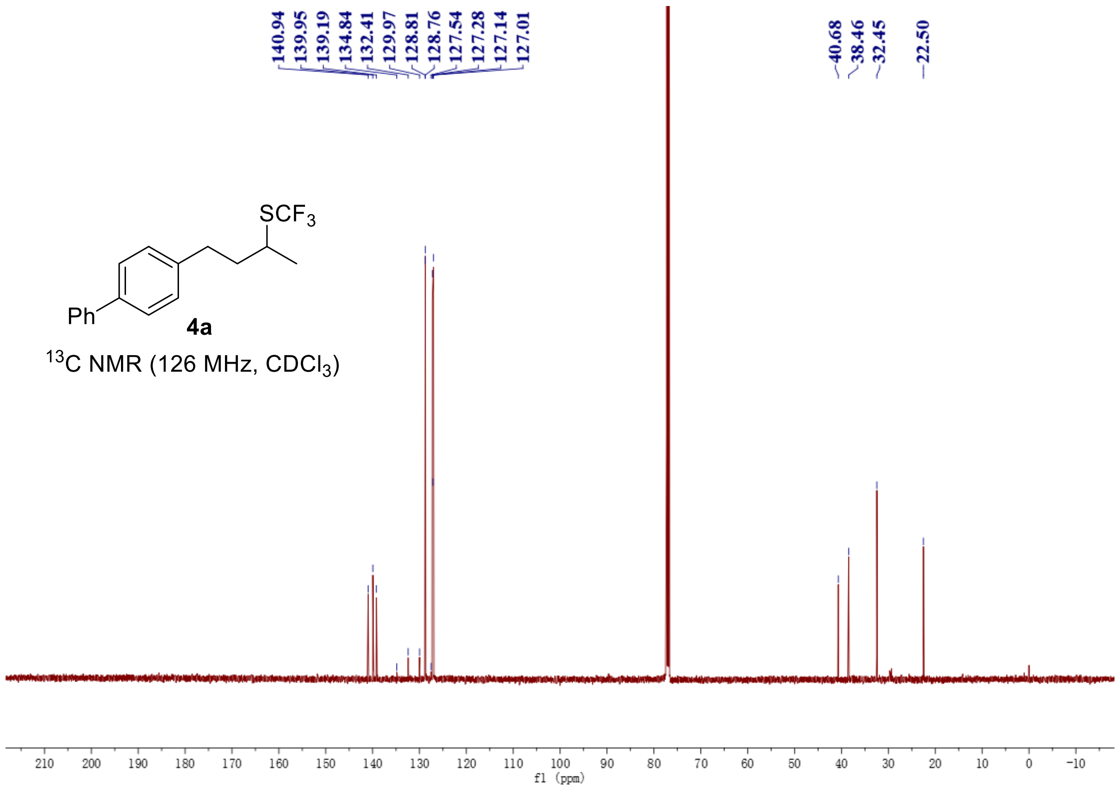

4a

^{13}C NMR (126 MHz, $CDCl_3$)

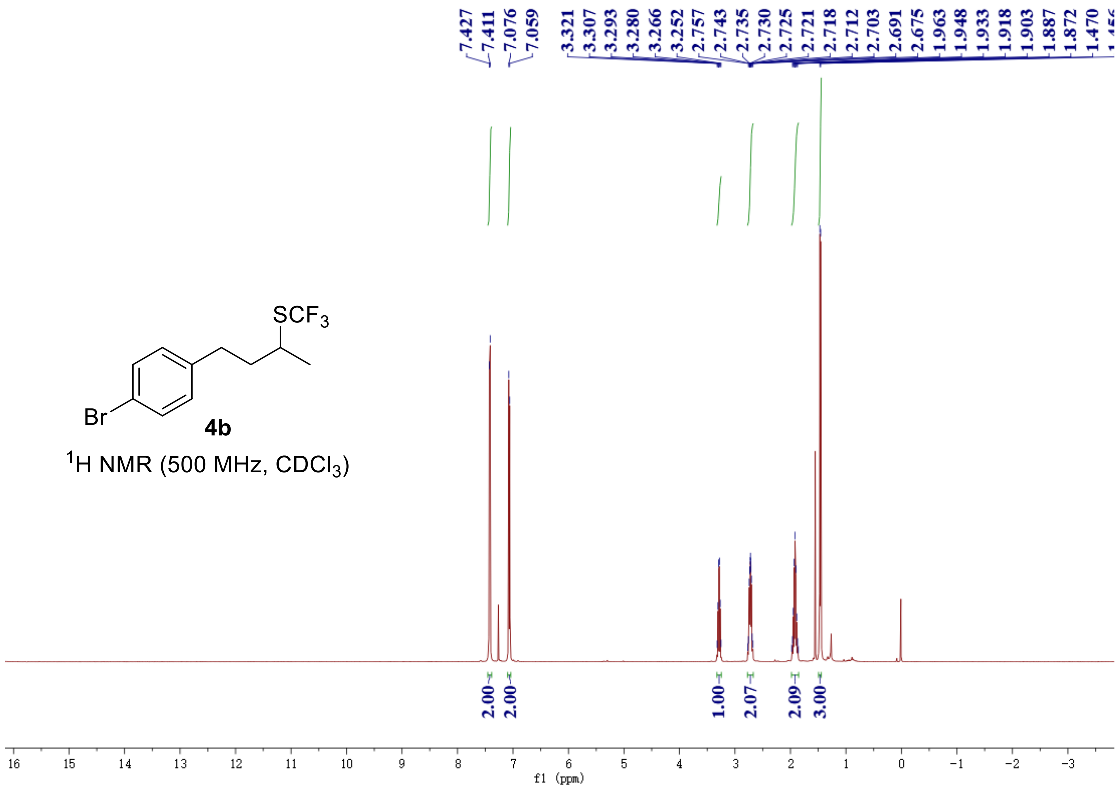

4b

^{1}H NMR (500 MHz, $CDCl_3$)

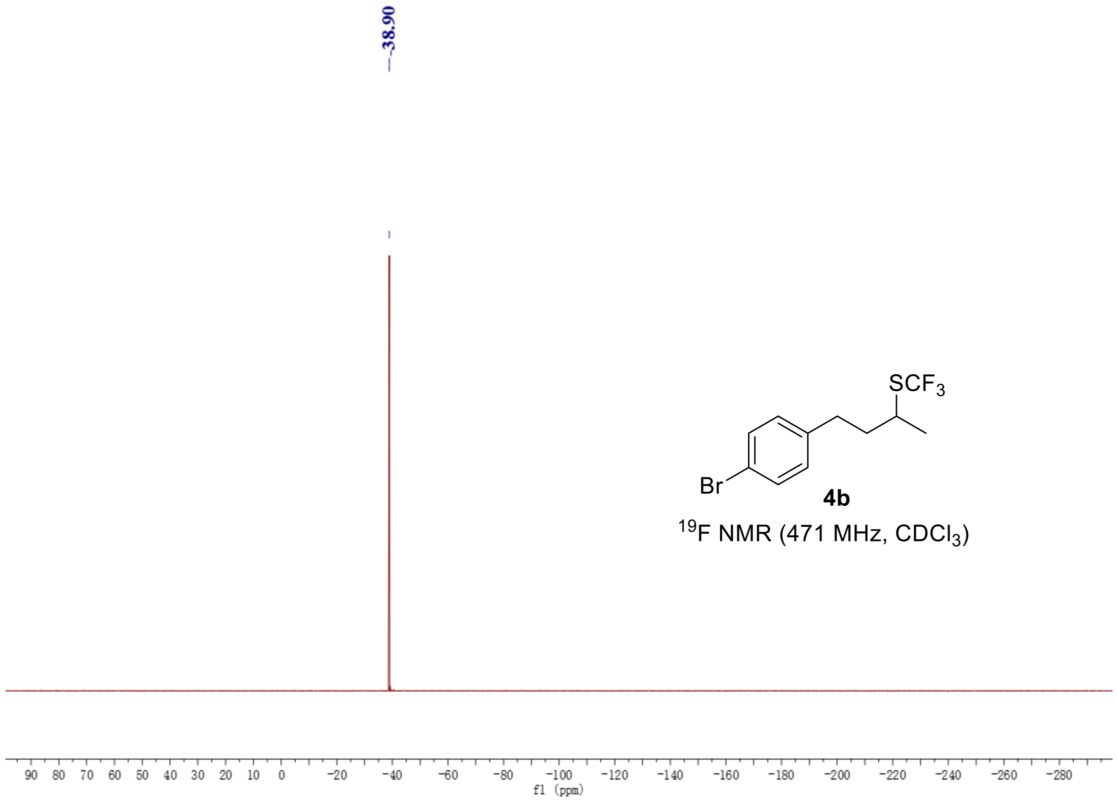
-38.90
SCF3
Br
4b
19F NMR (471 MHz, CDCl3)
f1 (ppm)

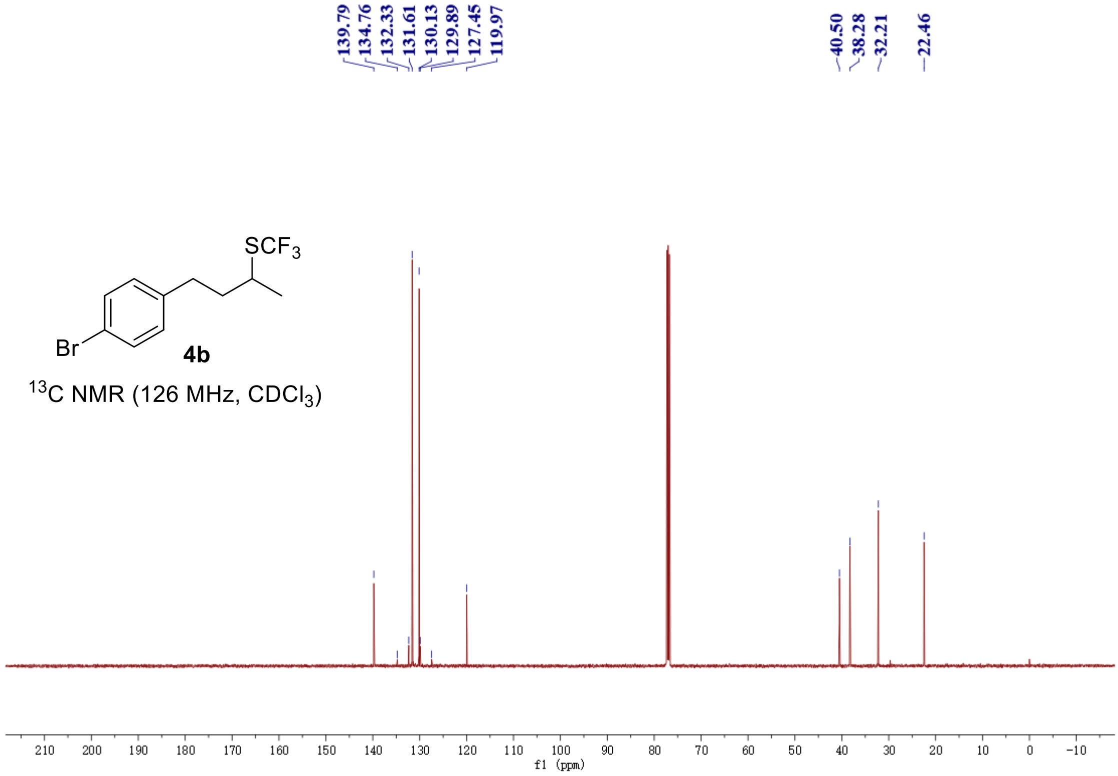
139.79
134.76
132.33
131.61
130.13
129.89
127.45
119.97
40.50
38.28
32.21
22.46
SCF3
Br
4b
13C NMR (126 MHz, CDCl3)
f1 (ppm)

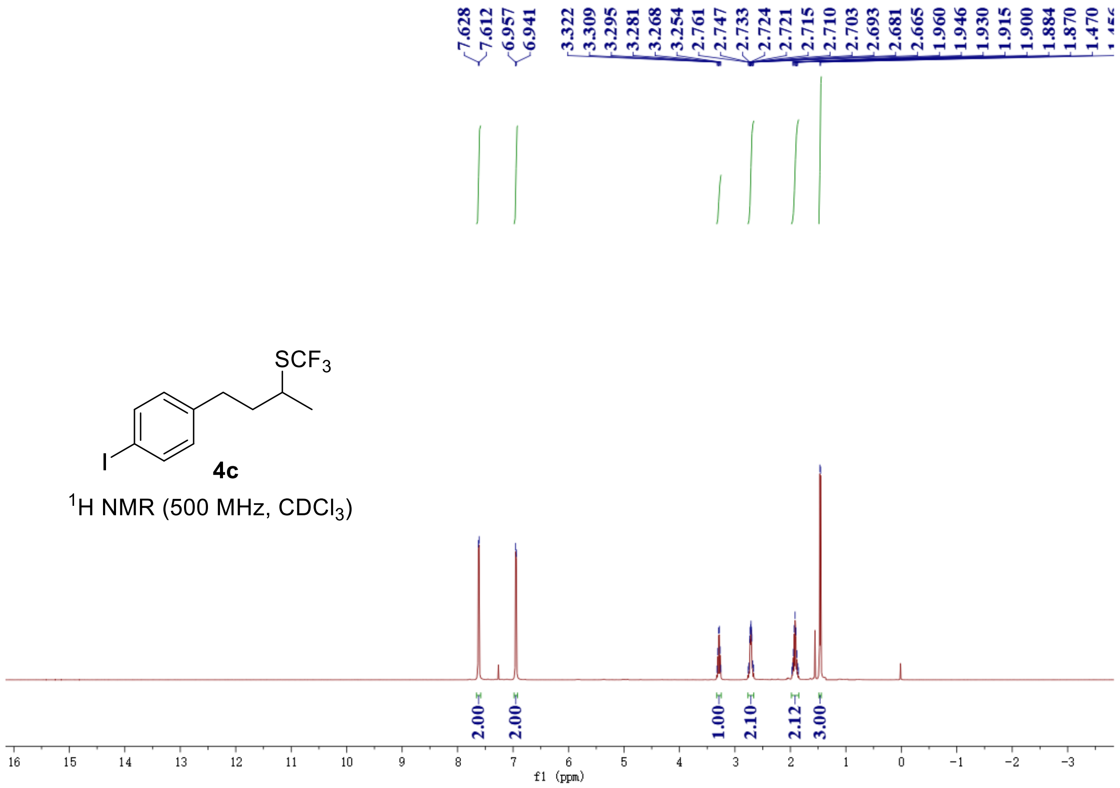
7.628
7.612
6.957
6.941
3.322
3.309
3.295
3.281
3.268
3.254
2.761
2.747
2.733
2.724
2.721
2.715
2.710
2.703
2.693
2.681
2.665
1.960
1.946
1.930
1.915
1.900
1.884
1.870
1.470
SCF3
I
4c
1H NMR (500 MHz, CDCl3)
2.00
2.00
1.00
2.10
2.12
3.00
f1 (ppm)

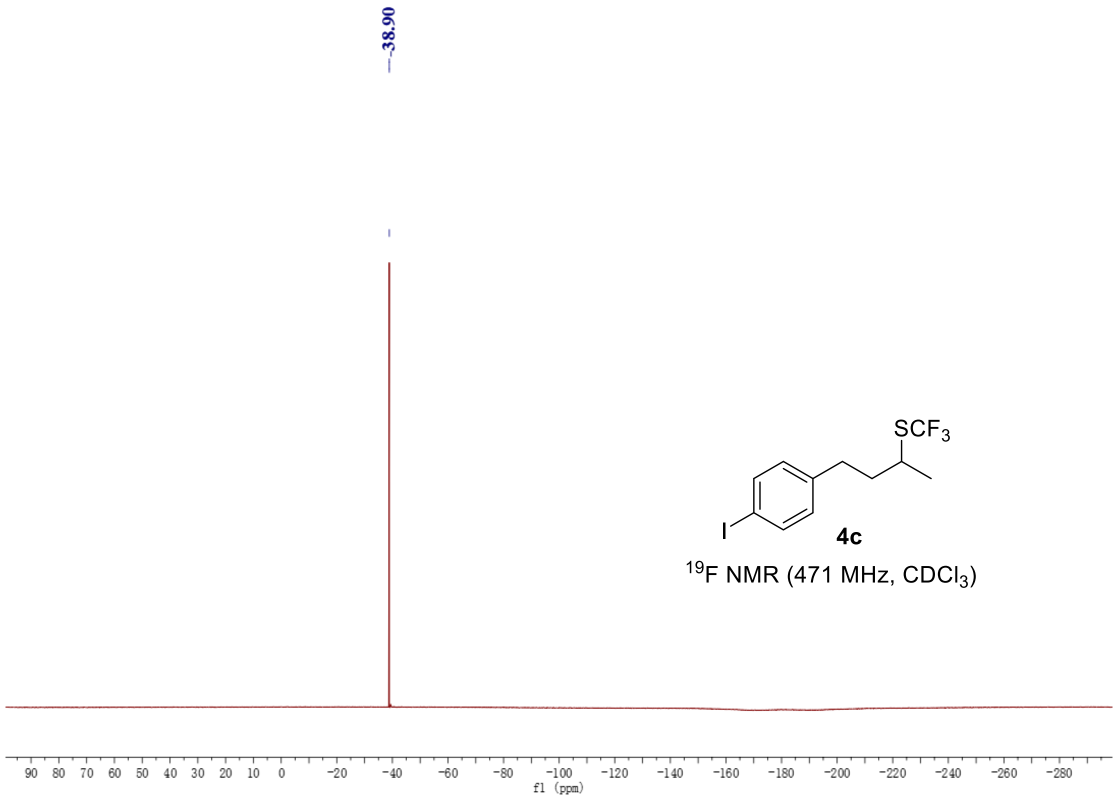
-38.90
SCF3
I
4c
19F NMR (471 MHz, CDCl3)
f1 (ppm)

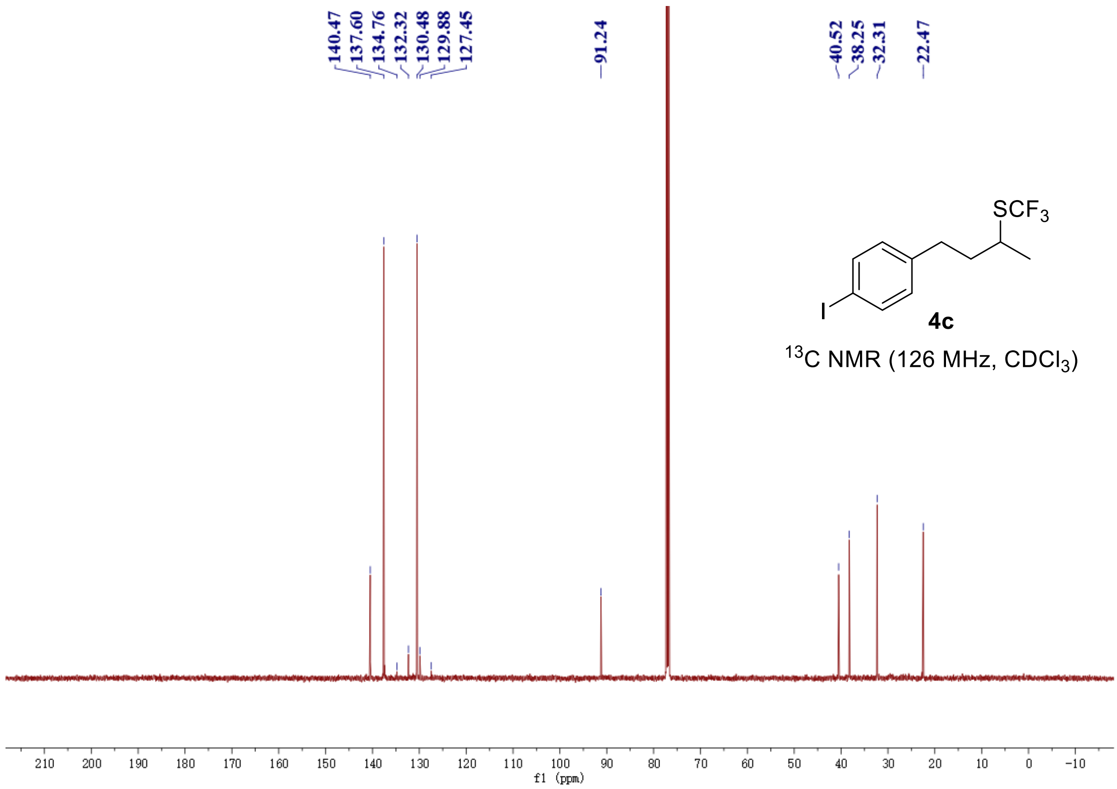
140.47
137.60
134.76
132.32
130.48
129.88
127.45
91.24
40.52
38.25
32.31
22.47
SCF3
I
4c
13C NMR (126 MHz, CDCl3)
f1 (ppm)

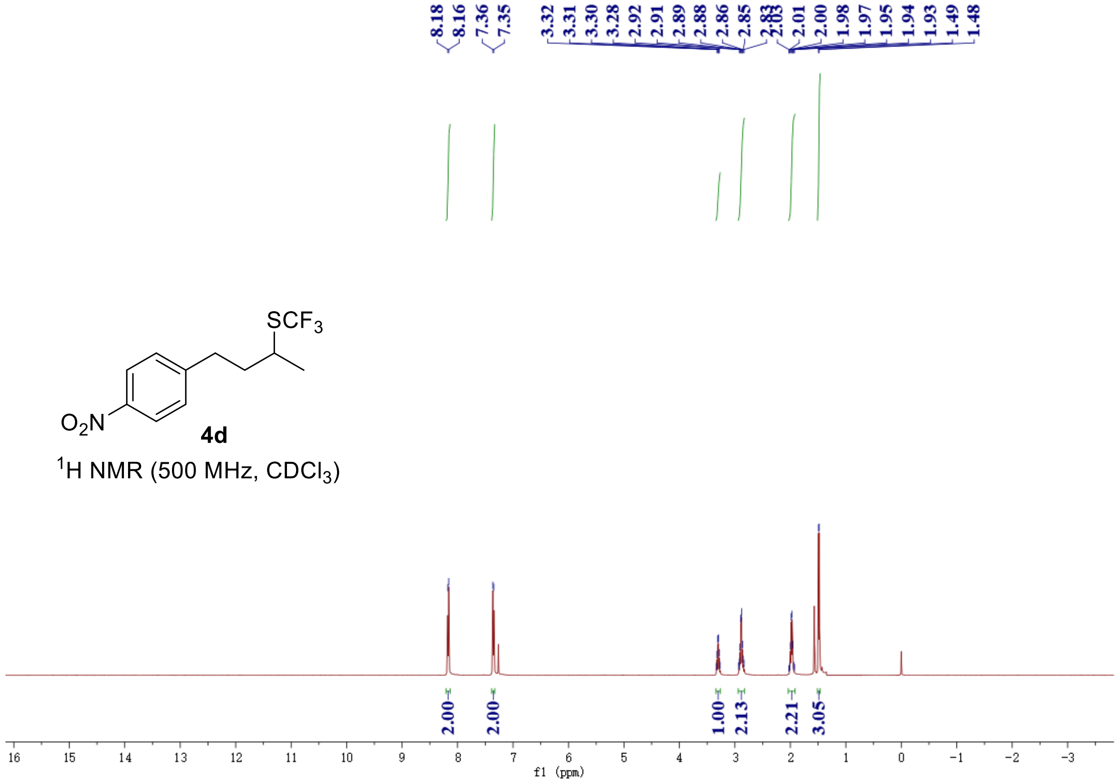
8.18
8.16
7.36
7.35
3.32
3.31
3.30
3.28
2.92
2.91
2.89
2.88
2.86
2.85
2.83
2.03
2.01
2.00
1.98
1.97
1.95
1.94
1.93
1.49
1.48
SCF3
O2N
4d
1H NMR (500 MHz, CDCl3)
2.00
2.00
1.00
2.13
2.21
3.05
f1 (ppm)

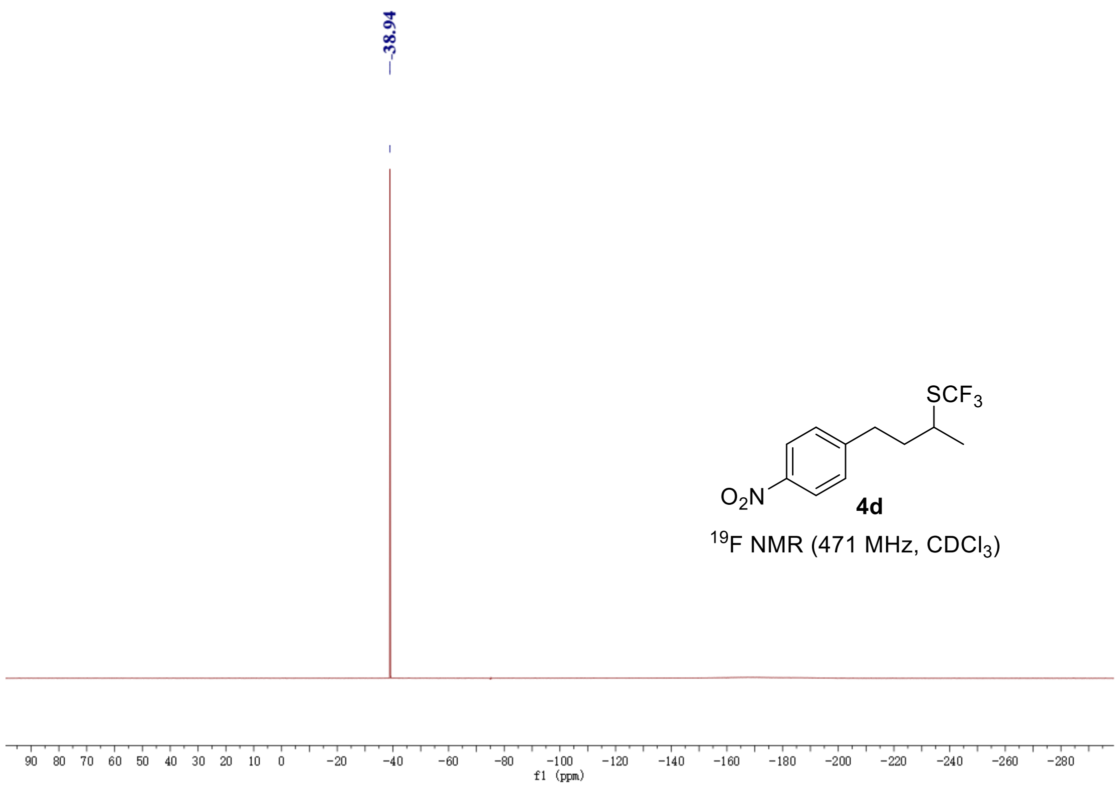

-38.94
SCF3
O2N
4d
19F NMR (471 MHz, CDCl3)
90 80 70 60 50 40 30 20 10 0 -20 -40 -60 -80 -100 -120 -140 -160 -180 -200 -220 -240 -260 -280
f1 (ppm)

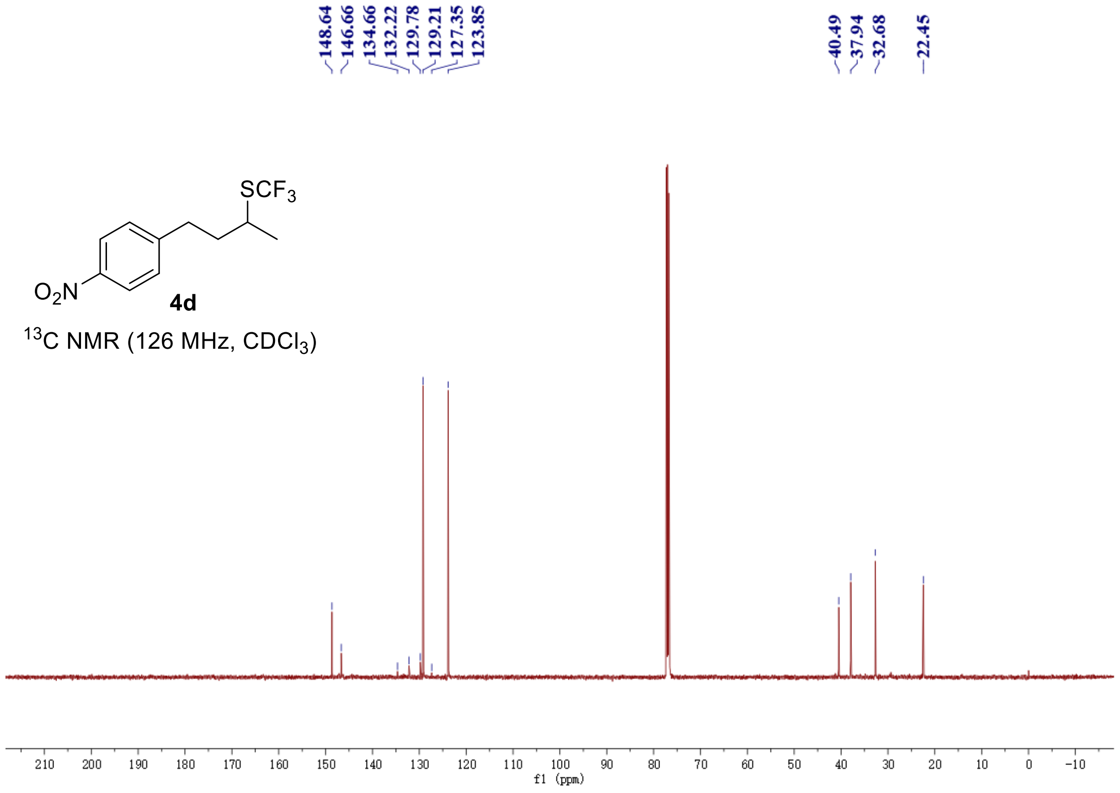

148.64
146.66
134.66
132.22
129.78
129.21
127.35
123.85
40.49
37.94
32.68
22.45
SCF3
O2N
4d
13C NMR (126 MHz, CDCl3)
210 200 190 180 170 160 150 140 130 120 110 100 90 80 70 60 50 40 30 20 10 0 -10
f1 (ppm)

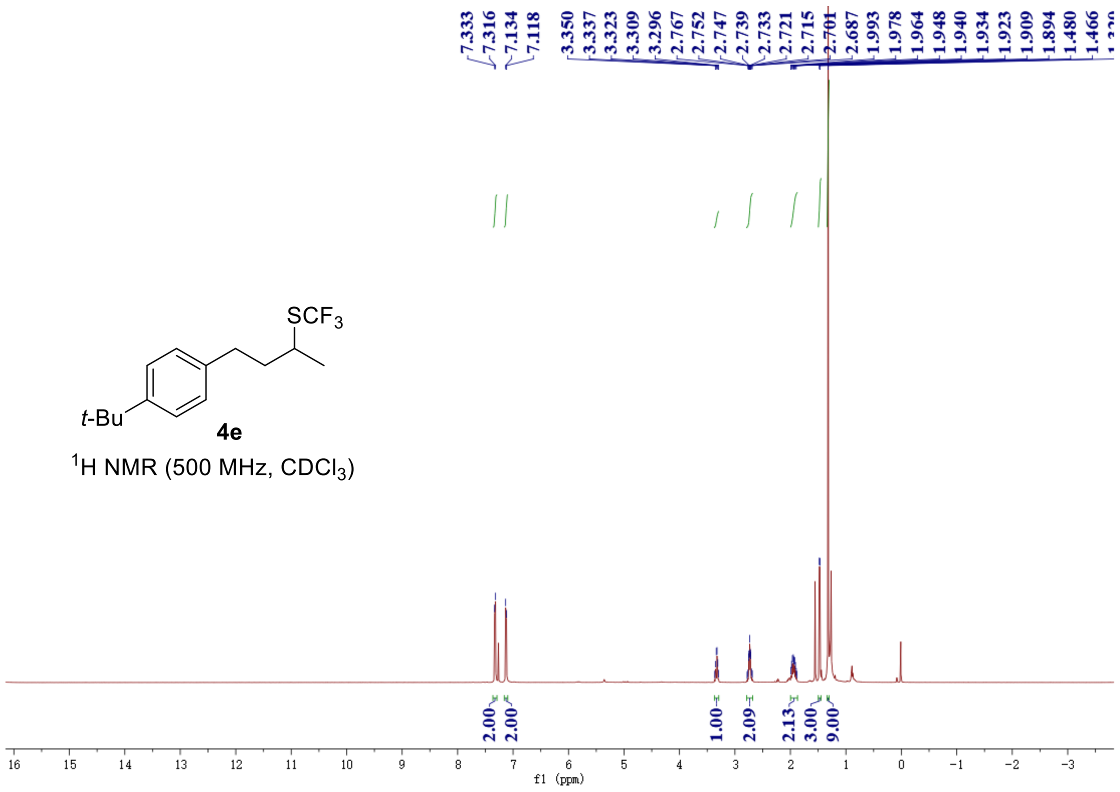
7.333
7.316
7.134
7.118
3.350
3.337
3.323
3.309
3.296
2.767
2.752
2.747
2.739
2.733
2.721
2.715
2.701
2.687
1.993
1.978
1.964
1.948
1.940
1.934
1.923
1.909
1.894
1.480
1.466
SCF3
t-Bu
4e
1H NMR (500 MHz, CDCl3)
2.00
2.00
1.00
2.09
2.13
3.00
9.00
f1 (ppm)

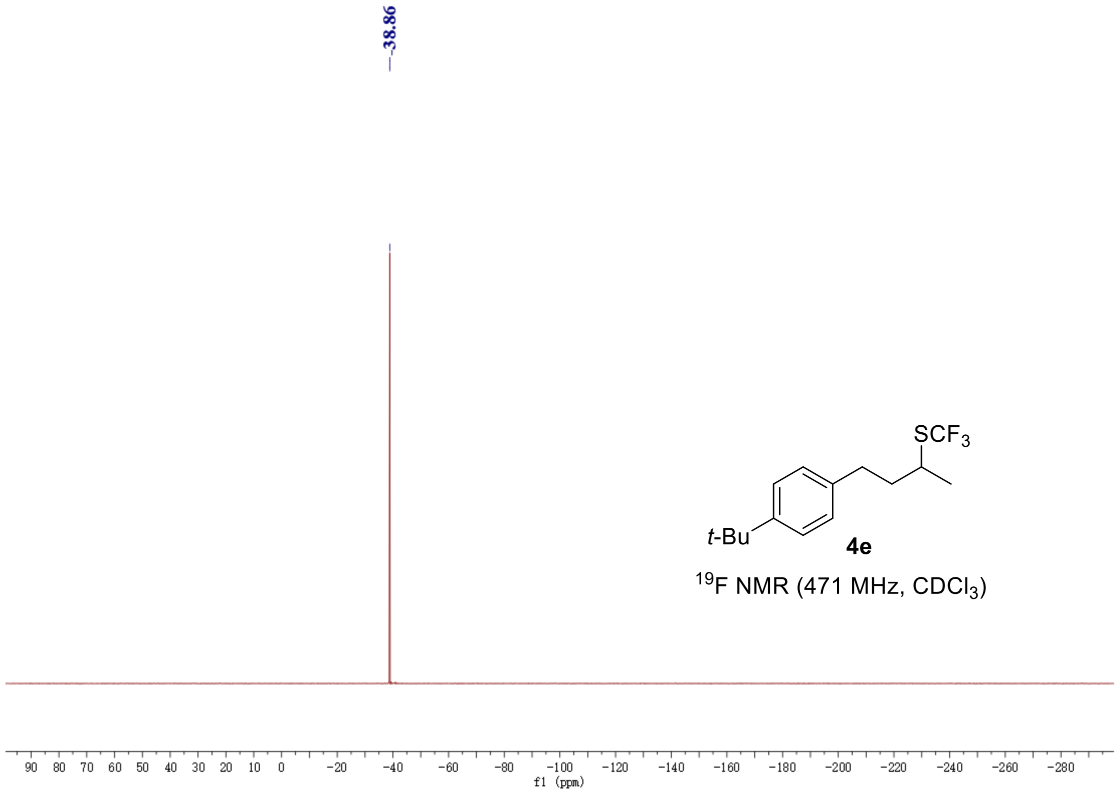
-38.86
SCF3
t-Bu
4e
19F NMR (471 MHz, CDCl3)
f1 (ppm)

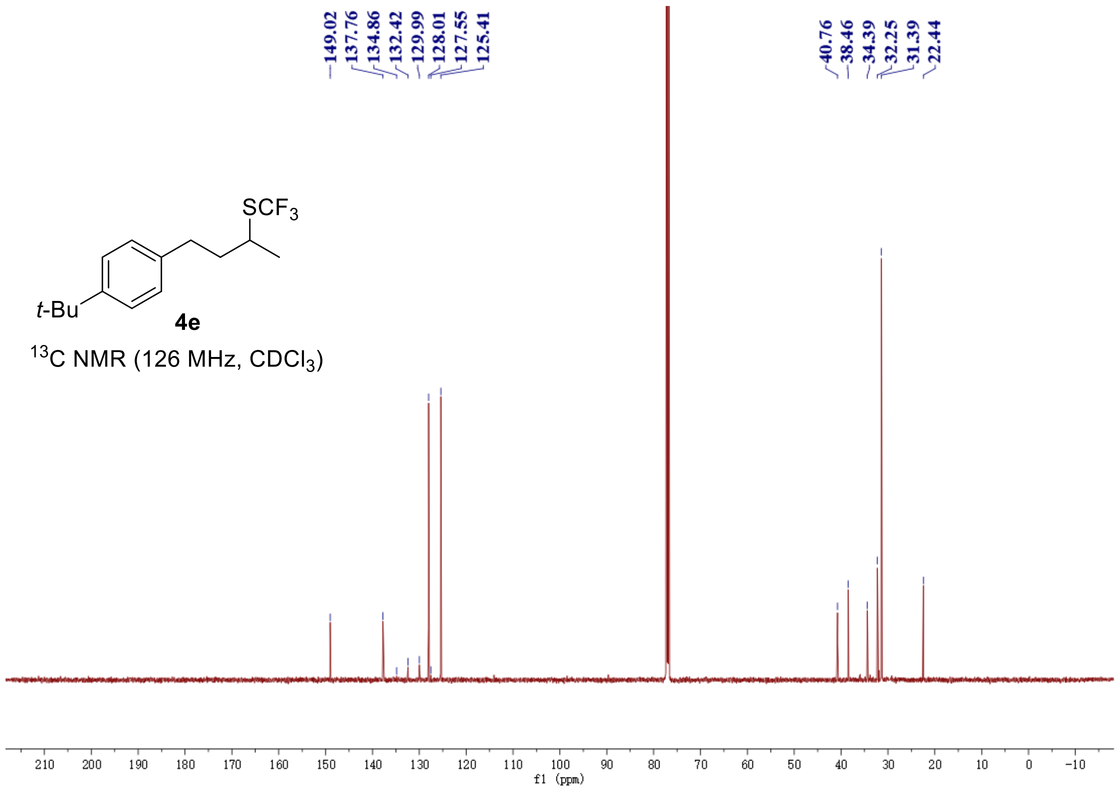

149.02
137.76
134.86
132.42
129.99
128.01
127.55
125.41
40.76
38.46
34.39
32.25
31.39
22.44
SCF3
t-Bu
4e
13C NMR (126 MHz, CDCl3)
210
200
190
180
170
160
150
140
130
120
110
100
90
80
70
60
50
40
30
20
10
0
-10
f1 (ppm)

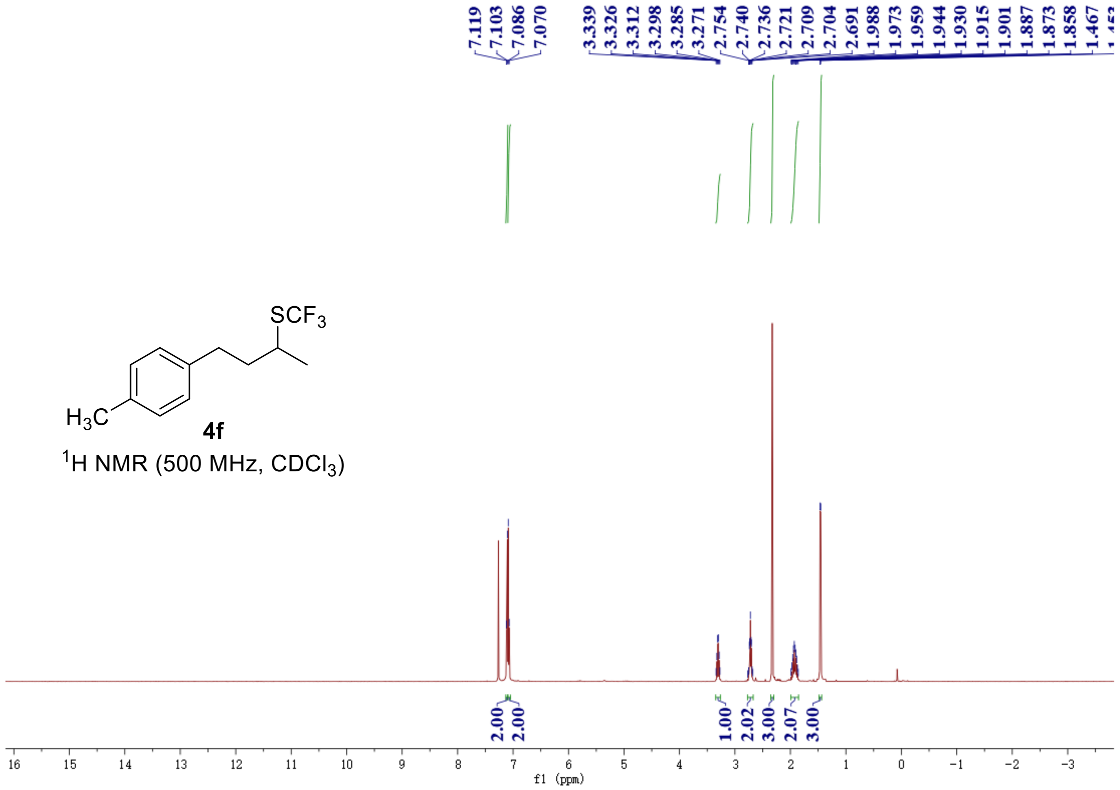

7.119
7.103
7.086
7.070
3.339
3.326
3.312
3.298
3.285
3.271
2.754
2.740
2.736
2.721
2.709
2.704
2.691
1.988
1.973
1.959
1.944
1.930
1.915
1.901
1.887
1.873
1.858
1.467
SCF3
H3C
4f
1H NMR (500 MHz, CDCl3)
2.00
2.00
1.00
2.02
3.00
2.07
3.00
16
15
14
13
12
11
10
9
8
7
6
5
4
3
2
1
0
-1
-2
-3
f1 (ppm)

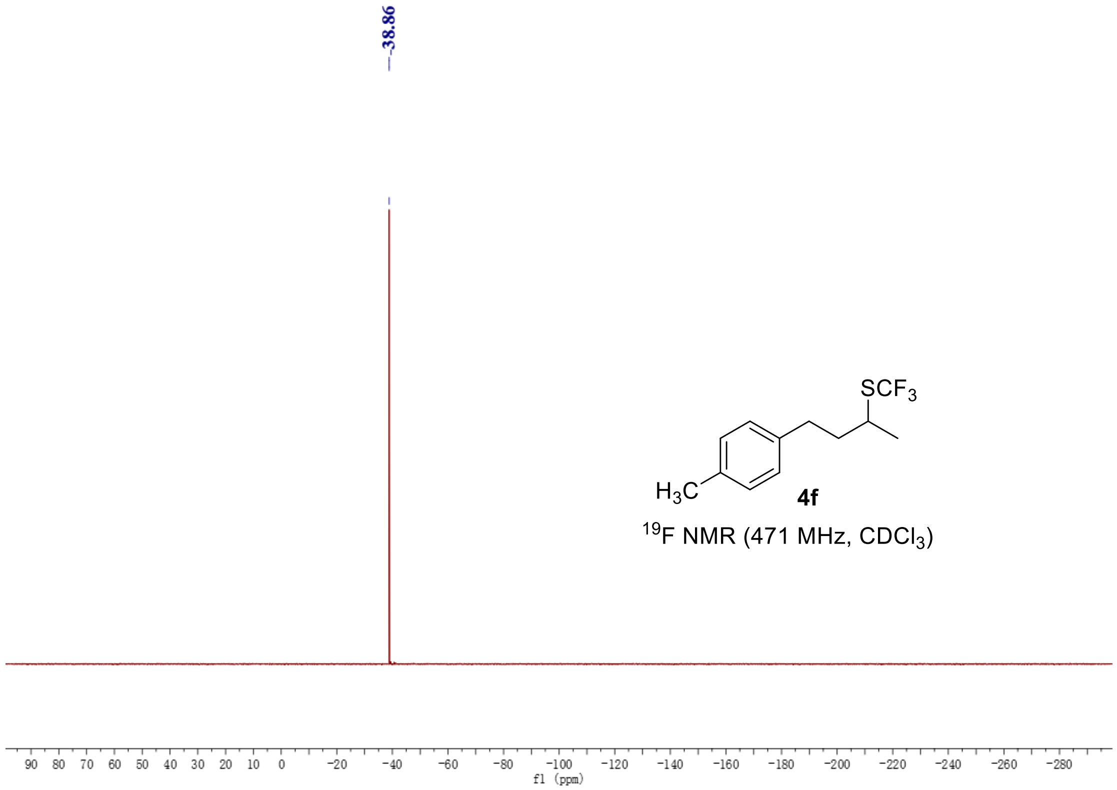
-38.86
SCF3
H3C
4f
19F NMR (471 MHz, CDCl3)
f1 (ppm)

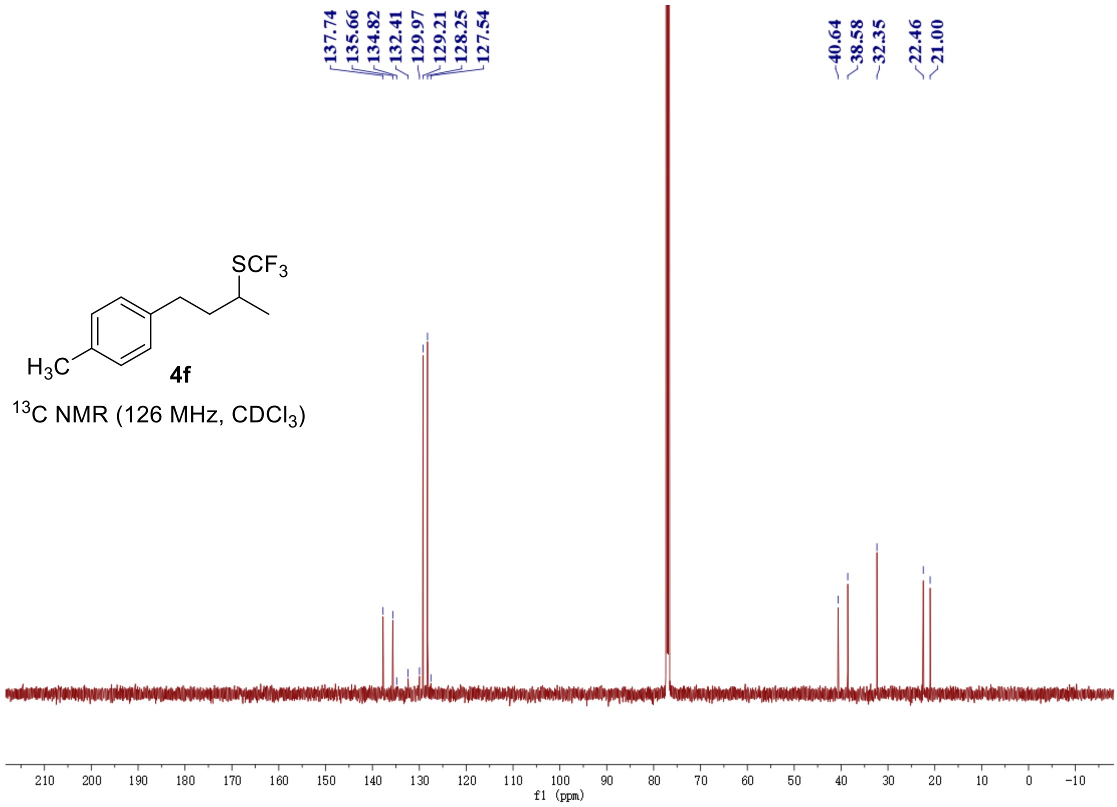
137.74
135.66
134.82
132.41
129.97
129.21
128.25
127.54
40.64
38.58
32.35
22.46
21.00
SCF3
H3C
4f
13C NMR (126 MHz, CDCl3)
f1 (ppm)

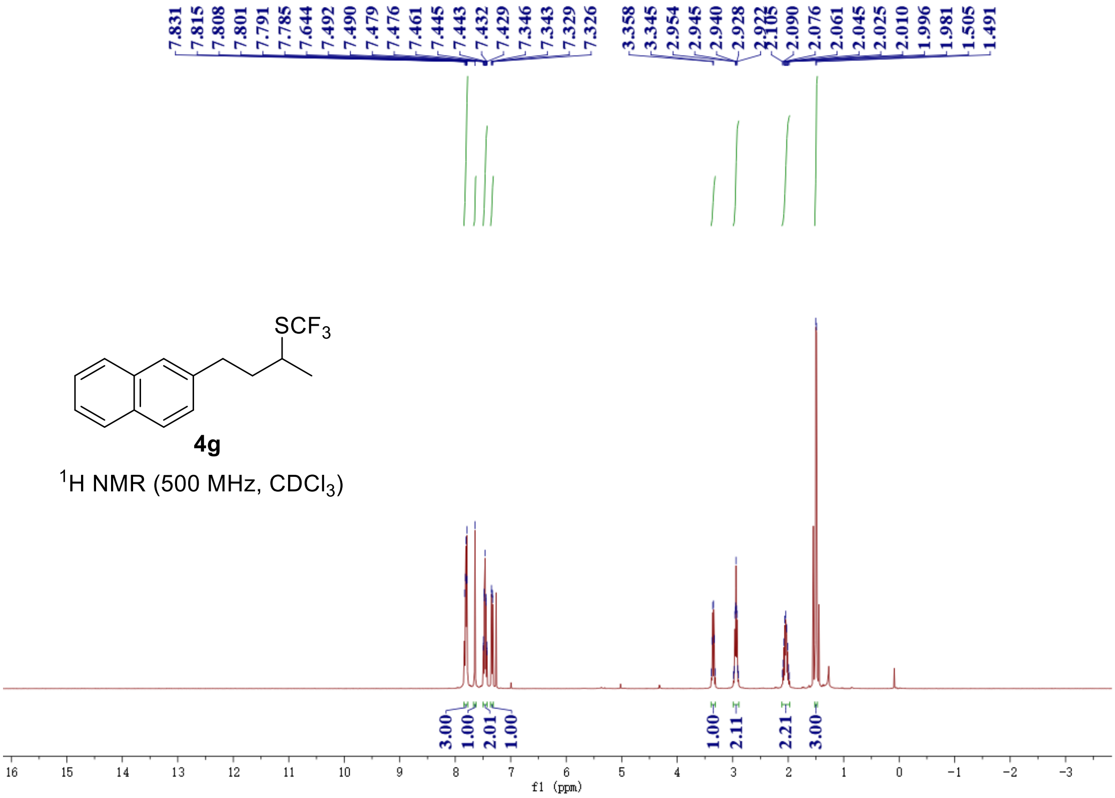

SCF3
4g
1H NMR (500 MHz, CDCl3)
f1 (ppm)

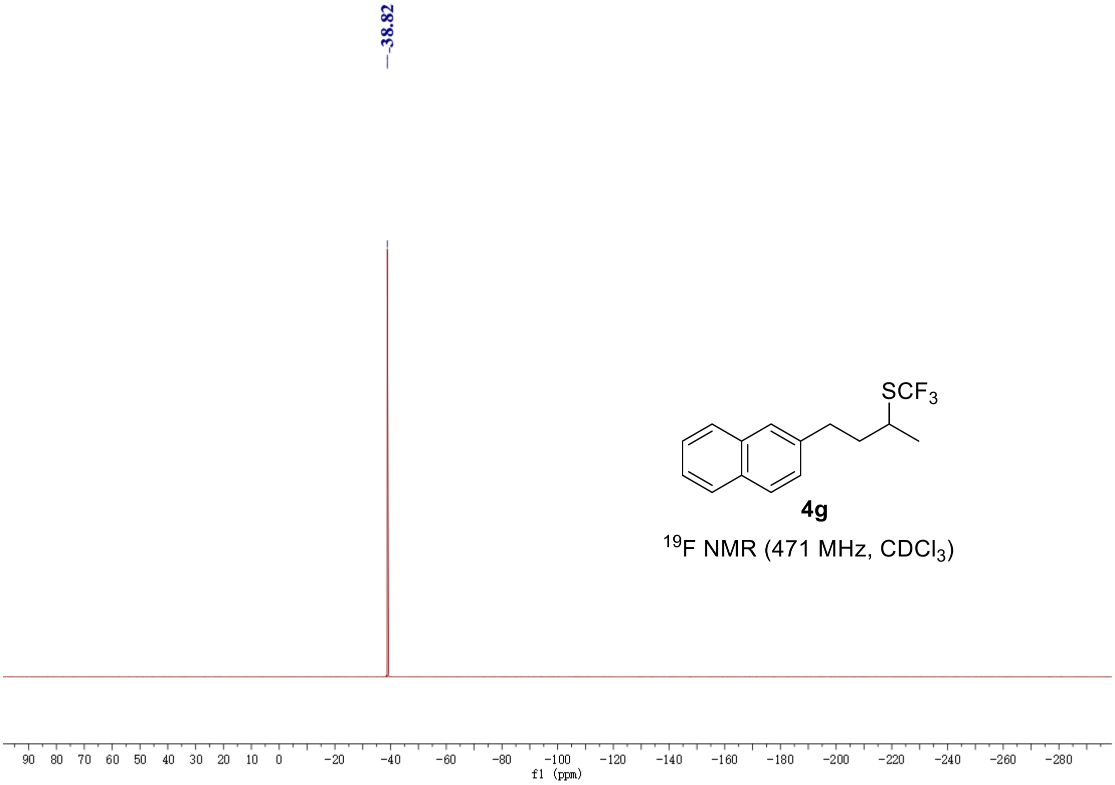

−38.82
SCF3
4g
19F NMR (471 MHz, CDCl3)
f1 (ppm)

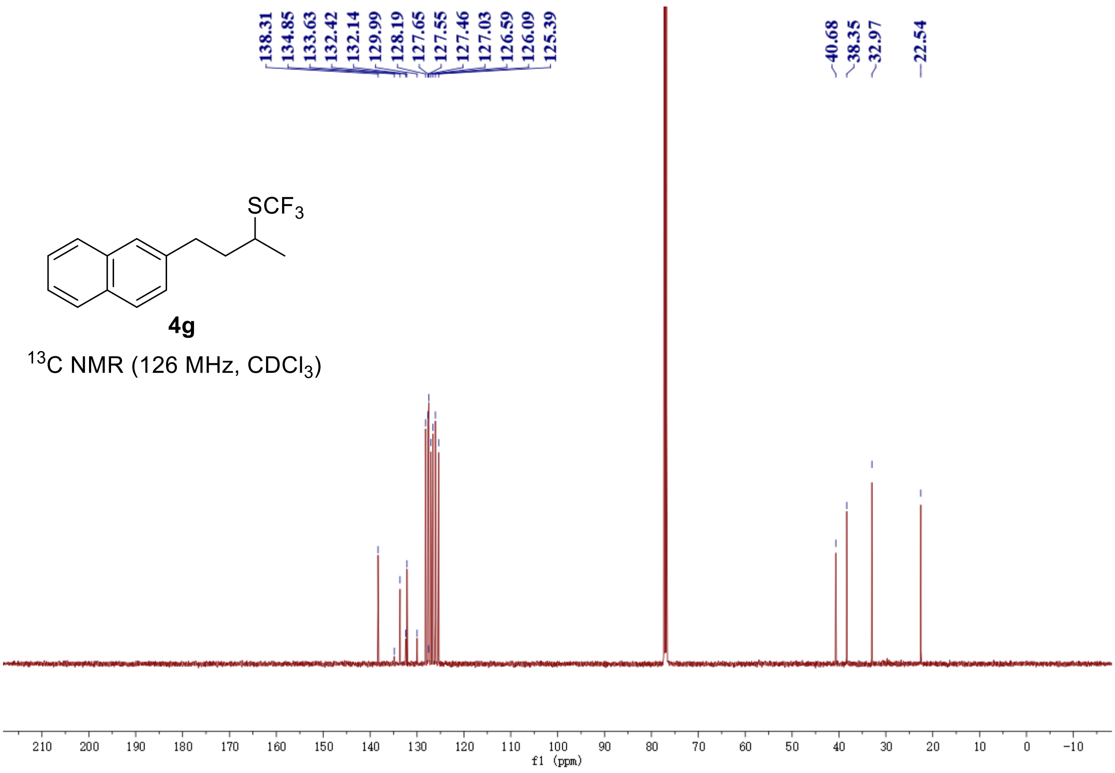

4g

^{13}C NMR (126 MHz, $CDCl_3$)
